# Synergy between a cytoplasmic vWFA/VIT protein and a WD40-repeat F-box protein controls development in *Dictyostelium*

**DOI:** 10.3389/fcell.2023.1259844

**Published:** 2023-09-14

**Authors:** Andrew W. Boland, Elisabet Gas-Pascual, Hanke van der Wel, Hyun W. Kim, Christopher M. West

**Affiliations:** ^1^ Department of Biochemistry and Molecular Biology, University of Georgia, Athens, GA, United States; ^2^ Complex Carbohydrate Research Center, University of Georgia, Athens, GA, United States; ^3^ Center for Tropical and Emerging Global Diseases, University of Georgia, Athens, GA, United States

**Keywords:** cellular slime mold, E3(SCF)ubiquitin-ligase, F-box protein, glycosylation, VIT, von willebrand domain A, RING

## Abstract

Like most eukaryotes, the pre-metazoan social amoeba *Dictyostelium* depends on the SCF (Skp1/cullin-1/F-box protein) family of E3 ubiquitin ligases to regulate its proteome. In *Dictyostelium*, starvation induces a transition from unicellular feeding to a multicellular slug that responds to external signals to culminate into a fruiting body containing terminally differentiated stalk and spore cells. These transitions are subject to regulation by F-box proteins and O_2_-dependent posttranslational modifications of Skp1. Here we examine in greater depth the essential role of FbxwD and Vwa1, an intracellular vault protein inter-alpha-trypsin (VIT) and von Willebrand factor-A (vWFA) domain containing protein that was found in the FbxwD interactome by co-immunoprecipitation. Reciprocal co-IPs using gene-tagged strains confirmed the interaction and similar changes in protein levels during multicellular development suggested co-functioning. FbxwD overexpression and proteasome inhibitors did not affect Vwa1 levels suggesting a non-substrate relationship. Forced FbxwD overexpression in slug tip cells where it is normally enriched interfered with terminal cell differentiation by a mechanism that depended on its F-box and RING domains, and on Vwa1 expression itself. Whereas *vwa1*-disruption alone did not affect development, overexpression of either of its three conserved domains arrested development but the effect depended on Vwa1 expression. Based on structure predictions, we propose that the Vwa1 domains exert their negative effect by artificially activating Vwa1 from an autoinhibited state, which in turn imbalances its synergistic function with FbxwD. Autoinhibition or homodimerization might be relevant to the poorly understood tumor suppressor role of the evolutionarily related VWA5A/BCSC-1 in humans.

## Introduction

As cells grow, replicate, and differentiate, their proteomes must be remodeled to meet their needs. The ubiquitin proteasome system helps address these needs using highly regulated assemblies of poly-ubiquitin ligases to target proteins for degradation by the 26S proteasome. Cullin-1 Ring Ub ligases, also known as Skp1/Cullin-1/F-box protein (SCF) complexes, comprise a major and highly evolutionarily conserved family. They utilize a cullin (Cul1) scaffold and Rbx1 to bind Ub-charged E2 subunits in proximity to the Skp1 adaptor and a substrate presenting F-box protein (FBP) (Willems et al., 2004; Harper and Schulman, 2021)). Canonical FBPs have a substrate binding domain located C-terminal to a ∼40 amino acid F-box domain that interfaces with Skp1. These substrate receptor (SR) domains, which include WD40 repeat propellers, Leucine-rich repeats (LRR), Armadillo-repeats, and others, confer specificity to the SCF (Skaar et al., 2013). While many FBPs and their substrates have been characterized in humans, yeast, and *Arabidopsis*, the majority of FBP substrates are yet to be elucidated. Furthermore, the evolutionary diversity of FBPs complicates their characterization in unrelated organisms. Thus the roles of the SCFs in the vast kingdom of protists, which includes many pathogens that seriously impact human and animal health, as well as agriculture, remain unknown.

The amoebozoa are a group of protists that lie near the base of the amorphea branch of eukaryotic evolution that gave rise to fungi, yeast, and animals (Schaap and Schilde, 2018). *Dictyostelium discoideum* is well-studied representative and prior work establishes that the SCF complex actively regulates its development (West and Blader, 2015; Kim et al., 2022). *D. discoideum* amoebae live freely in the soil as unicellular organisms when nutrients are plentiful. Upon nutrient depletion, the cells aggregate into motile slugs that differentiate into prespore cells and various subtypes of prestalk cells. These slugs will follow environmental cues and ultimately culminate at the soil surface into fruiting bodies consisting of terminally differentiated spore and stalk cells with cellulose-rich cell walls (Bonner and Lamont, 2005). This process is dependent on concerted transcriptional (Rosengarten et al., 2015; Santhanam et al., 2015) and proteomic (Bakthavatsalam and Gomer, 2010; Czarna et al., 2010; González-Velasco et al., 2019) changes. The significance of proteomic changes is emphasized by the effects on development of mutations in FBPs (e.g., Ennis et al., 2000; Mohanty et al., 2001) and inhibitors of the ubiquitin-proteasome system (Pergolizzi et al., 2019; Boland et al., 2022; Kim et al., 2022).

SCF assembly and activity is increasingly understood to be regulated by a number of factors including Cand1, chaperones, localization factors, post-translational modifications, etc. (Wang et al., 2020). A novel form of regulation of the SCF has been discovered in *Dictyostelium*. Its Skp1 subunit is post-translationally modified by hydroxylation of a proline in an oxygen dependent manner and subsequent glycosylation with a pentasaccharide (Teng-umnuay et al., 1998). Loss of the prolyl hydroxylase responsible for generating the anchor point for the Skp1-glycan modifies the O_2_-setpoint for permitting its transition from the motile slug stage of development to mature fruiting bodies (West et al., 2007), by a mechanism that involves the proteasome (Boland et al., 2022). The addition of the glycan is required for mediating the O_2_-dependence (Wang et al., 2009; Wang et al., 2011), and has been shown to increase Skp1’s level of interaction with several F-box proteins, including the WD40 domain containing FBP FbxwD (Sheikh et al., 2015; Boland et al., 2022). Building from findings in *Dictyostelium*, the Skp1 modification has been described in the human parasite *Toxoplasma gondii* (Xu et al., 2012a; Rahman et al., 2016) and the plant pathogen *Pythium ultimum* (van der Wel et al., 2019), and the modification genes are evident in many other unicellular organisms including pathogenic fungi (West and Blader, 2015).

FbxwD is of interest because its ectopic overexpression delays development (Sheikh et al., 2015) and, like many other FBPs, its level of interaction with Skp1 is promoted by the Skp1 modification. FbxwD appears to be a canonical WD40-repeat substrate receptor FBP, except for its unusual N-terminal RING domain-like sequence. RING domains are a subset of Zn-finger domains, and occur in other classes of Ub-ligases where they mediate direct interaction with Ub-charged E2 subunits and activate Ub transfer (Metzger et al., 2014). While not unprecedented (Cenciarelli et al., 1999; Kuroda et al., 2002; Zielke et al., 2006), RING domains are unusual in F-box proteins and a role in SCF activity is unknown. Here we find that the *fbxwD* gene resists deletion, and its overexpression in prestalk cells has inhibitory effects on terminal differentiation by a mechanism that depends on its RING and F-box domains.

Examination of the interactome of endogenously tagged FbxwD using co-immunoprecipitation combined with mass spectrometry yielded 2 uncharacterized proteins, Vwa1 and Vwa2, that are evolutionarily related to the nucleocytoplasmic VWA5A/BCSC-1 clade of VIT (Vault Inter-alpha Tryspin) and vWFA (von Willebrand Factor A) domain containing proteins (Whittaker and Hynes, 2002). The VIT-vWFA domain arrangement of Vwa1 is characteristic of a broad protein family that includes extracellular ITIH heavy chains (Himmelfarb et al., 2004; Zhuo et al., 2004), a specific intracellular poly ADP-ribose polymerase (PARP4) (Perina et al., 2014), and the intracellular tumor suppressing VWA5A/BCSC-1 (Martin et al., 2003; Anghel et al., 2012; Di et al., 2018). As reviewed here, examples of intracellular VIT-vWFA proteins are found throughout eukaryotic phylogeny and some prokaryotes. The functions of their domains are generally unknown but they have been implicated in mediating protein-protein interactions. *Dictyostelium* Vwa1 has highest homology to the intracellular nucleocytoplasmic protein VWA5A/BCSC-1, which is also downregulated in a schizophrenia model (Szabo et al., 2021). Our domain overexpression and gene disruption studies of Vwa1 reported here indicate a role for Vwa1 in development that is synergistic with FbxwD, and we propose a structure-based model to explain the genetic interaction.

## Materials and Methods

### Cell culture and development

*Dictyostelium discoideum* strains (Table 1) were typically grown by shaking in HL-5 (Ashworth and Watts, 1970) axenic media at 22°C. Vegetative stage cells were collected by centrifugation at 1,000×*g* for 1 min during logarithmic growth (2.5–7.5×10^6^ cells/ml). Development was typically induced by collecting cells at the same growth phase density and depositing in phosphate buffer on nitrocellulose filters (4×10^7^ cells per 5-cm filter) or non-nutrient agar as previously described (Xu et al., 2012b). Aggregation stage and slug cells were normally collected at 10 or 14 h, respectively, or at a time of equivalent morphology if indicated. As described, development was also induced by washing and resuspending cells at 10^7^/ml in Agg buffer (0.01 M NaPO_4_, pH 6.0, 0.01 M KCl, 0.005 M MgCl_2_) and shaking for the indicated times. Strains were cloned by spreading on nutrient agar plates at low density in association with *Klebsiella aerogenes* and isolation from cleared plaques, and development was also monitored under these conditions. To assay spore differentiation, cells were recovered by rinsing filters after 36–40 h of development with 0.2% (v/v) Triton X-100, dispersed by vortexing and mild probe sonication, and spores were counted on a hemocytometer.

**TABLE 1:** Strains used in this study.

Strains	parental	genotype	resistance	ref.
Ax3	NC-4	axenic	none	a
Ax4	Ax3	axenic	none	b
HW540	Ax3	FbxwD-FLAG_3_	blasticidin-S	c
HW542	Ax3	FbxwD- FLAG_3_UBA_2_	blasticidin-S	this pub.
HW541	Ax3	FbxwA-FLAG_3_	blasticidin-S	this pub.
HW543	Ax3	FbxwA- FLAG_3_UBA_2_	blasticidin-S	this pub.
HW548	Ax3	*cotB*:: FLAG_3_FbxwD	G418	d
HW552	Ax3	*cotB*:: FLAG_3_FbxwD(LP-AA)	G418	d
HW553	Ax3	*ecmA*::FLAG_3_FbxwD	G418	this pub.
HW554	Ax3	*ecmA*::FLAG_3_FbxwD(LP-AA)	G418	this pub.
HW556	Ax3	*ecmA*::FLAG_3_FbxwD(V9A)	G418	this pub.
HW557	Ax3	*ecmA*::FLAG_3_FbxwD(CH22/24AA)	G418	this pub.
HW558	Ax3	*ecmA*::FLAG_3_FbxwD(PF41/42AA)	G418	this pub.
HW601	Ax3	Vwa1-FLAG_3_ (endogenous)	blasticidin-S	this pub.
HW602	Ax3	Vwa2-FLAG_3_ (endogenous)	blasticidin-S	this pub.
HW603a	Ax3	Vwa1-N1-FLAG_3_, cl. 5	blasticidin-S	this pub.
HW603b	Ax3	Vwa1-N1-FLAG_3_, cl. 11	blasticidin-S	this pub.
GWDI_133_C_2	Ax4	Vwa1-N2, insertion at nucleotide 46	blasticidin-S	e
HW632	WDI_133_C_2	*ecmA*::His_6_FLAG_3_Vwa1	G418	this pub.
HW634	GWDI_133_C_2	*cotB*::His_6_FLAG_3_Vwa1	G418	this pub.
HW604	HW603a	*dscC*::His_6_FLAG_3_Vwa1-VIT/Vwa1-N1	blast/G418	this pub.
HW605	HW603a	*dscC*::His_6_FLAG_3_Vwa1-vWFA/Vwa1-N1	blast/G418	this pub.
HW606	HW603a	*dscC*::His_6_FLAG_3_Vwa1-C-T/Vwa1-N1	blast/G418	this pub.
HW609	HW603a	*ecmA*::His_6_FLAG_3_FbxwD/Vwa1-N1	blast/G418	this pub.
HW611	Ax3	*dscC*::His_6_FLAG_3_Vwa1 medium expr.	G418	this pub.
HW612	Ax3	*dscC*::His_6_FLAG_3_Vwa1 high expr.	G418	this pub.
HW622	Ax3	*dscC*::His_6_FLAG_3_Vwa2	G418	this pub.
HW624	Ax3	*dscC*::His_6_FLAG_3_Vwa1-VIT	G418	this pub.
HW626	Ax3	*dscC*::His_6_FLAG_3_Vwa1-vWFA	G418	this pub.
HW628	Ax3	*dscC*::His_6_FLAG_3_Vwa1-C-T	G418	this pub.

a, Loomis, 1971; b, Parikh et al., 2010; c, Boland et al., 2022; d, Sheikh et al., 2015; e, Gruenheit et al., 2021.

### Endogenous gene tagging

The C-termini of Vwa1 and Vwa2 were modified to include a FLAG_3_-tag as previously described for CulE (Sheikh et al., 2015). Targeting DNA fragments ranging from 620–1,032 bp were generated by PCR using oligonucleotide primers described in Supplementary Table S1, gDNA as a template, and proofreading polymerase (Q5, New England Biolabs). The targeting sequences were used to replace the *culE* targeting sequences in the pVS_CulE-FLAG_3_ plasmid (Sheikh et al., 2015) based on matching restriction sites in the PCR primers and pVSculE-5′3′, as described in Supplementary Figure S6. The modified plasmids, containing a sequence expected to encode FLAG_3_/EcoRI/BirA/NcoI/TEV protease site/BamHI/TAA at the C-terminus, were linearized with BssHII, gel purified, and electroporated into strain Ax3 cells essentially as described (Pang et al., 1999). After selection in the presence of 10 μg/ml Blasticidin S, clones were screened for expected modifications by western blotting using mouse mAb M2 anti-FLAG antibody (Sigma-Aldrich), and confirmed by PCR.

A strain in which the C-terminus of FbxwD (dictyBase DDB_G0292312) is similarly FLAG_3_-tagged was described previously (Boland et al., 2022). A related strain in which the C-terminus is tagged with FLAG_3_ and two ubiquitin-binding domains (UBA_2_) in tandem was produced as follows. A double stranded nucleotide sequence, which encodes a GS-rich linker, a FLAG_3_ epitope, a BirA biotinylation site, a linker, and 2 copies of a UBA domain (Supplementary Figure S13), was synthesized by DNA2.0. The FLAG_3_ epitope and BirA biotinylation site sequence were previously described in pVS3 (Sheikh et al., 2015), and the Uba_2_ sequence was derived from DDB_G0286357, which encodes RcbA, the predicted ortholog of Rad23 from *Saccharomyces cerevisiae* (Mark et al., 2016). For tagging the C-terminus of FbxwD, this insert was excised by digestions with BglII and BamH1, and cloned into the previously described FLAG_3_-tagging construct for FbxwD (Boland et al., 2022) after similar digestion. The resulting plasmid was cleaved with BssHII and PvuII, and the insert, which included the above FLAG_3_UBA_2_ coding sequence in tandem with *bsr*-resistance cassette, was used to edit the *fbxwD* locus of strain Ax3, based on double cross-over homologous recombination. Gene editing was confirmed by PCR and Western blotting, and detection of FbxwD, FbxwA, and RcbA peptide sequences in the anti-FLAG co-IPs (Supplementary Table S2). Strains are listed in Table 1.

The strategy for C-terminal tagging of FbxwA with FLAG_3_ was similar (Boland et al., 2022). The previously described pVSCulE5′3′-BsR (Sheikh et al., 2015) was modified to replace culE targeting sequences with *fbxwA* targeting sequences. The 5′-targeting sequence was amplified from strain Ax3 genomic DNA using primers FbxA5′-S and FbxA5′-AS (Supplementary Table S1). After cloning into pCR4-TOPO, the insert (1,042 nt) was excised with BssHII and BglII and cloned into similarly digested pVSCulE5′3′-BsR. Similarly, the 3′-targeting sequence (992 nt) was amplified using FbxA3′-S and FbxA5′-AS, and cloned into the PstI and PvuII sites of the plasmid. After excision with BssHII and PvuII as above, the tagging construct was electroporated into strain Ax3 cells and Blasticidin-S resistant cells cloned and analyzed by PCR to confirm the expected insertion, and by Western blotting to confirm expression of the tagged protein.

### Protein overexpression

Overexpression of epitope-tagged versions of Vwa1, Vwa2, and FbxwD was accomplished essentially as described (Sheikh et al., 2015). Strains are listed in Table 1. The entire ORF of *Vwa1* was amplified from genomic DNA of strain Ax3 via PCR and inserted into the pMiniT (New England Biolabs) vector, named pCR4vwa1, using oligonucleotides described in Supplementary Table S1 and Supplementary Figure S1. The amplified DNA fragments were trimmed by digestion with NcoI and BamHI, gel purified, and cloned into similarly digested and gel-purified pV3D (discoidin 1γ semi-constitutive promotor), pV3C (*cotB* pre-spore cell specific promotor), or pV3E (*ecmA* pre-stalk cell specific promotor) (West et al., 2007). Circular plasmids were electroporated into cells and transformants were selected for by growth in the presence of 20 μg/ml G418 as above. The sequence of the N-terminally tagged FLAG_3_Vwa1 is shown in Supplementary Figure S1B. Expression was validated via western blotting with murine mAb M2 anti-FLAG antibody (Sigma-Aldrich) (Supplementary Figures S7, S9).

Overexpression of discrete regions of Vwa1 was accomplished by PCR amplification of genomic DNA using oligonucleotides described in Supplementary Figure S1 and Supplementary Table S1, cloning of the amplicons into pV3D, and transformation of strain Ax3 or HW603a (Vwa1-N1).

To achieve overexpression of FLAG_3_FbxwD in prestalk cells, its full-length coding region was cloned into pV3E exactly as described for its cloning into pV3C (Sheikh et al., 2015). Point mutations were introduced by site-directed mutagenesis using the oligonucleotide pairs described in Supplementary Table S1. Sequences are described in Figure 3B, and protein expression shown in Figure 3D.

### Vwa1 gene editing

The intermediate plasmid pMiniT-Vwa1 was linearized with NsiI, located at the codon amino acid 195 of Vwa1. The previously mentioned FLAG_3_ and associated blasticidin resistance cassette fragment was amplified from pVS_CulE-FLAG_3_ with the primers VWA1_KO_FGBSR and VWA1_KO_BSR_R (Supplementary Table S1), which included homology arms flanking the NsiI digestion site. Ligation independent cloning using NEBuilder^®^ HiFi DNA Assembly Cloning Kit assembled the fragments as illustrated (Supplementary Figure S6). The disruption DNA was excised with PvuI, and used to edit the *vwa1* locus as described above. Blasticidin-resistant clones were screened using PCR and Western blotting with pAb UOK162 on vegetative cells grown in HL5 (Supplementary Figure S6).

### Preparation of cytosolic extracts

Before lysis, vegetative cells were rinsed twice in 17 mM potassium phosphate (pH 6.5) by centrifugation in the cold at 3,000 × *g* for 1 min. Slug cells were scraped from cellulose nitrate filters and dissociated in 20 mM sodium EDTA, 50 mM Tris-HCl (pH 7.4) by shearing through a 26-gauge syringe needle. Once dissociated, cells were centrifuged and resuspended twice in the potassium phosphate buffer, and finally rapidly re-suspended in ice cold 250 mM sucrose, 50 mM Tris-HCl (pH 7.4), 10 μg/ml leupeptin, 10 μg/ml aprotinin, 1 μM PMSF, and immediately forced through a 3- or 5-µm nuclepore filter to lyse the cells. Lysates were centrifuged at 100,000 × *g* for 1 h to produce a soluble S100 cytosolic supernatant. The S100 was used for Western blotting for soluble proteins, to minimize interference from particulate materials.

### Generation of Vwa1 anti-sera

7×10^10^ vegetative cells expressing FLAG_3_His_6_Vwa1 under control of the discoidin (*dscC*) promotor were filter lysed to generate a cytosolic S100 fraction after ultracentrifugation. The supernatant was loaded onto and eluted from 200-ml DEAE column using gradient of NaCl. Fractions containing intact Vwa1 based on Western blot analysis using anti-FLAG mAb M2 were subsequently purified on a 1-ml His-trap with stepwise imidazole elution. The purified Vwa1 was concentrated using spin columns and 1.2–1.5 mg of purified protein was run on a gradient slab gel. After Coomassie staining, the Vwa1 band was excised and detained. The destained gel slice was submitted to Cocalico Biologicals, Inc. where it was processed to immunize two rabbits with one initial dose and two boosters. Pre-bleeds, post-boost, and exsanguination bleeds were screened against lysates of Ax3, Vwa1-N1, Vwa1^oe^, Vwa2^oe^ cells by Western blotting to characterize their specificity. Antisera pAb UOK161 and UOK162 were used to visualize Vwa1 in slug and vegetative cells respectively due to the presence of interfering cross-reactive bands in the other cell type.

### Western blotting

Cell lysates or fractions were solubilized in Laemmli Sample Buffer containing 50 mM dithiothreitol and boiled for 5 min. Approximately 4×10^5^ vegetative cells and 8×10^5^ slug cells or cell equivalents were loaded in wells of a 1-mm 4%–12% Bis/Tris NuPage polyacrylamide SDS gel (Invitrogen), using MES or MOPS running buffer depending on the *M*_r_ range of target proteins. Gels were transferred to nitrocellulose using an iBlot 2 (Invitrogen) dry blotting system, and blots were blocked in 5% (w/v) nonfat dry milk in Tris-buffered saline (TBS, 100 mM NaCl, 50 mM Tris-HCl, pH 7.5), followed by overnight incubation with primary Abs diluted in the blocking solution, washed in TBS, and probed with Alexa Flour-680-conjugated secondary Abs. Primary Abs included anti-Vwa1 pAb UOK161, anti-Vwa1 pAb UOK162, anti-FLAG mAb M2 (Sigma), anti-FLAG mAb 12C6c (Developmental Studies Hybridoma Bank), 1:1,000 anti-fucose mAb 83.5 against *Dictyostelium* spore coat proteins (West, 2003). Fluorescence intensities were recorded on a LiCor Odyssey scanner and quantitated by densitometry using NIH ImageJ. Uncalibrated OD was used for the densitometry scale on gray-scale images of blots. Values were corrected by systematic subtraction of an adjacent blank area, and normalized to similar densitometric analysis of the same lane (80–100 kDa range) of the Coomassie blue stained gel after blotting.

### Co-immunoprecipitations

Protein interactomes were investigated using a co-IP approach as described (Boland et al., 2022). Briefly, cells were lysed for 15 min on ice in Lysis buffer (250 mM NaCl, 50 mM Tris-HCl (pH 8.0), 0.2% (v/v) NP-40, 10 μg/ml leupeptin, 10 μg/ml aprotinin) at a final concentration of 1.2–1.5×10^5^ cells/µl for vegetative cells and 2.4×10^5^ cells/µl for slugs. Lysates were spun at 21,000×*g* for 15 min to remove the insoluble material. 10^7^ cell equivalents of the supernatant (S21) were used to resuspend 5 µl packed volume of anti-FLAG mAb M2 magnetic Sepharose beads (Sigma-Aldrich). After rotation for 1 h at 4°C, the beads were collected magnetically, rinsed ×3 in 20 bead vol of Lysis buffer, 3× in detergent-free Lysis buffer, and once more in 20 vol of the unbuffered salt solution. For Western blot analysis, beads were boiled in 10 vol 2% SDS for 5 min. For mass spectrometric analysis, beads were eluted with 10 bead vol of 133 mM triethanolamine for 15 min and neutralized with acetic acid. Samples were dried by vacuum centrifugation and resolubilized in 8 M urea in 50 mM Tris-HCl (pH 8.0). The efficiency of IP capture, which was routinely evaluated by Western blot analysis of the S21, IP supernatant, and eluted fractions, was used to optimize the bead:extract ratio to achieve >80% capture.

### Mass spectrometric analysis

The proteomics work-flow was as previously described for the Skp1 interactome (Boland et al., 2022). Briefly, samples were solubilized in 8 M urea, reduced and alkylated with chloroacetamide, digested with endo Lys-C and trypsin, and recovered on a C18 Zip-Tip. The peptide solution was loaded onto a C18 trap column (Thermo Acclaim™ PepMap™ 100 C18 series) in a Thermo Fisher UltiMate 3,000 nano-HPLC, and eluted from the trap column onto a C18 nano-column (Thermo Acclaim™ PepMap™ 100 C18 series) in a 5%–90% acetonitrile gradient in 0.1% formic acid over 3 h. The eluent was directly introduced via a nano-electrospray source into a Thermo-Fischer Q-Exactive Plus and analyzed by MS and MS/MS. Full MS scans were acquired from *m*/*z* 350 to 2000 at 70,000 resolution. Peptides were selected for fragmentation in the C-trap via higher energy collision-induced dissociation for MS/MS analysis using a Top 10 method and a 30 s fragmentation exclusion window. Sequest HT search parameters were 10 ppm parent ion mass tolerance, 0.02 Da fragment ion tolerance, and up to 2 missed tryptic cleavages; variable modifications: oxidation of Met, formylation or acetylation of the protein N terminus; fixed modification: carbamidomethylation of Cys. False Discovery Rate (FDR) was determined via Target/Decoy in Proteome Discoverer 2.5. Peptide were assigned at a false discovery rate of <1% and protein identifications were classified by protein false discovery rate. Proteins were quantified from reconstructed ion chromatograms of all peptides assigned to a protein at the MS1 level. Proteins whose abundances were >4-fold higher in experimental vs. control samples with a Wilcoxon test *p*-value <0.01 (Wilson et al., 2020) and a t-test *p*-value <0.01 were classified as significant interactors. The mass spectrometry proteomics data are deposited in the ProteomeXchange Consortium via the PRIDE partner repository. Dataset identifiers for FbxwD-FLAG_3_ co-IP’s are PXD035633 and Project DOI: 10.6019/PXD035633, and for FLAG_3_Vwa1 co-IP’s are PXD035634 and Project DOI: 10.6019/PXD035634.

### Alignment and tree building

Domain boundaries for Vwa1 and Vwa2 (see Supplementary Figures S1, S2) were based on sequence conservation and correlation with folded domains of ITIH1 based on its crystal structure (Briggs et al., 2020). BLASTp was used to search for related Vwa1-vWFA and Vwa1-VIT domains in the non-redundant NCBI database (Supplementary Figure S5). Preliminary alignments were performed using Geneious Prime 2019.1.3 with a PAM250 cost matrix, Gap open penalty of 20, and Gap extension penalty of 10. The alignment was manually refined with priority given to hydrophobic regions and minimizing gaps. The evolutionary relationship of the sequences was explored using a UPGMA (unweighted pair group method with arithmetic mean) tree building method with 1,000 bootstrapping iterations and no %bootstrap cutoff. Tree files were exported as newick files and visualized in iTOL (Letunic and Bork, 2021).

### Structure and domain interface analysis

AlphaFold 2.2.1 (Jumper et al., 2021; Varadi et al., 2021) was accessed using NMRbox server (Maciejewski et al., 2017) and used to calculate and predict the structure of DdVwa1. The protein sequence was written as.FASTA file and used as input for AlphaFold using the following command: [alphafold proteinseq.FASTA]. A similar prediction is also found at https://alphafold.ebi.ac.uk/entry/Q54DV3. The previously calculated structure of HsVwa5a (Uniprot ID: O00534) used for comparison was retrieved from AlphaFold database webpage (alphafold.ebi.ac.uk). The top scoring modes were used for analysis and were visualized and imaged using PyMOL (PyMOL Molecular Graphics System, Version 2.2.0 Schrödinger, LLC). HsITIH1 crystal structure coordinates used for comparison were retrieved via PDB database using the accession code 6FPY.

The interface elements including hydrogen bonds and packing interactions between the vWFA and CTD domains of DdVwa1 were analyzed using PISA server (Krissinel, 2015) (www.ebi.ac.uk/pdbe/pisa/). The input coordinate file was curated by designating residues 1–615 as peptide chain A and residues 815–918 as peptide chain B.

## Results

### FbxwD interacts with VIT-vWFA domain containing proteins

**FIGURE 1:**
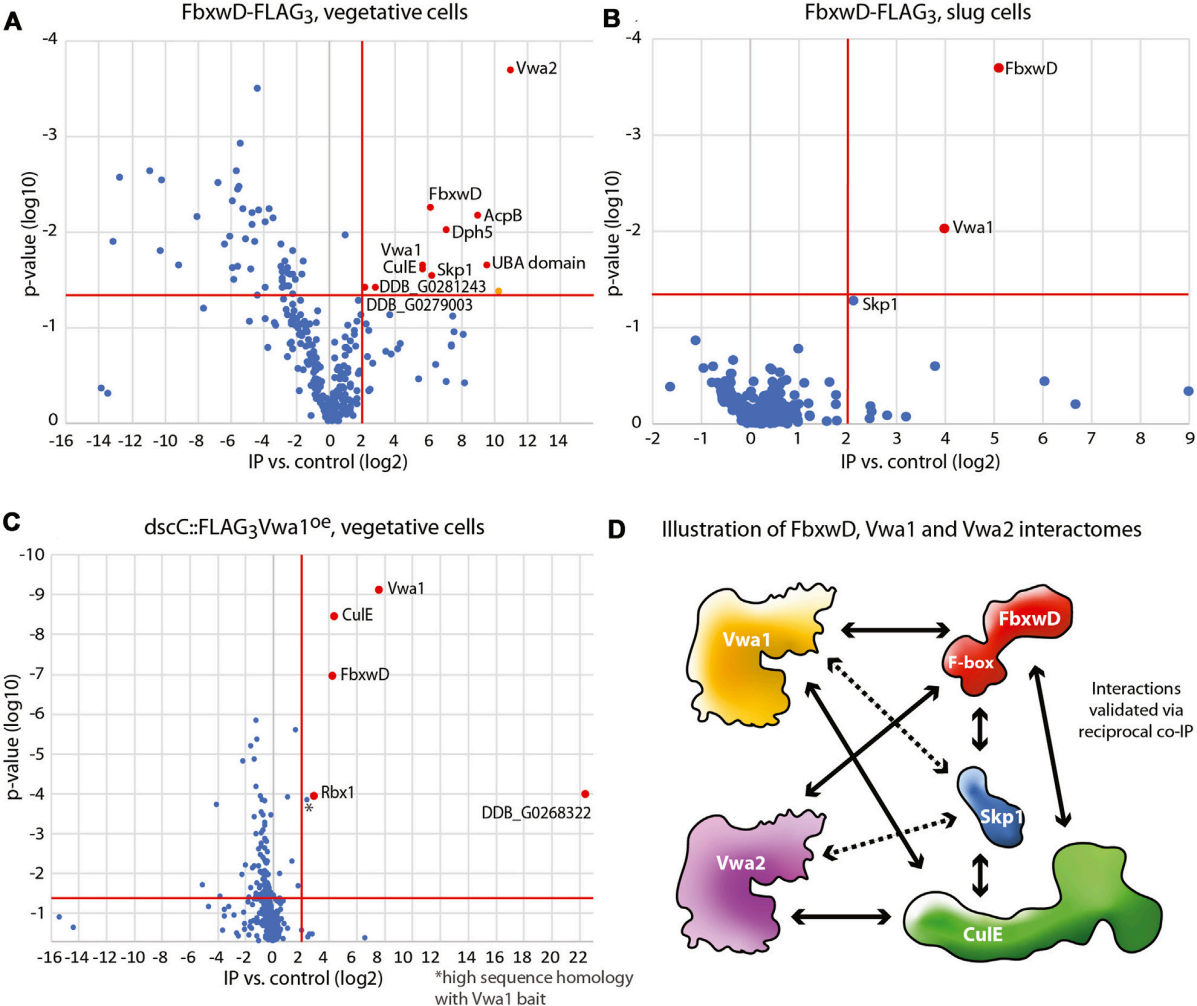
Interactomes of FbxwD-FLAG_3_ and FLAG_3_Vwa1. Immunoprecipitations of FLAG tagged targets using anti-FLAG mAb M2 from cells solubilized in non-ionic detergent (0.2% NP-40) were subjected to a proteomics work-flow that included generation of peptides with endo Lys-C and trypsin followed by detection by nLC MS/MS and quantitation by spectral counting. Data are represented as volcano plots of protein assignment confidence (ordinate, inferred from FDR) vs. enrichment in FbxwD-FLAG_3_ or FLAG_3_Vwa1 co-IPs relative to untagged Ax3 cells (abscissa). Proteins that were 4-fold higher in tagged vs. control strains with a *p* value of <0.05, as indicated by the red boundaries, were colored red and classified as interaction candidates. Data summarize results from 3 biological replicates, each consisting of 3 technical replicates. **(A)** Co-IP of endogenously tagged FbwxD-FLAG_3_ from vegetative stage (growing) cells. The orange-colored datum was excluded as a mitochondrial protein. **(B)** Co-IP of FbxwD-FLAG_3_ from slug cells. **(C)** Co-IP of vegetative cells overexpressing FLAG_3_Vwa1. **(D)** A summary of the FbxwD interactome as observed experimentally. Arrowheads designate the targets captured by the bait at the other end of the arrow. Dashed lines indicate interactions that did not satisfy all criteria. See Supplementary Table S2 for numerical data.

Previous studies identified several dozen FBP candidates in *Dictyostelium*, and many of these have predicted substrate receptor (SR) domains (Sheikh et al., 2015; Boland et al., 2022). However, their SR sequences are so unrelated to those of known FBPs that identifying their substrates is likely to require experimental approaches. FbxwD was chosen for further analysis because it is an abundant validated WD40 repeat FBP that interacts directly with Skp1 in a way that is enhanced when Skp1 is prolyl hydroxylated and glycosylated. A previously described strain in which the endogenous *fbxwD* locus was edited to modify the C-terminus of FbxwD with a FLAG_3_ tag (Boland et al., 2022) and a new strain tagged with FLAG_3_ in tandem with a pair of predicted Ub-binding (ubiquitin-associated, UBA) domains from the *Dictyostelium* ortholog of yeast Rad23 (see Methods), were used to probe the FbxwD interactome where FbxwD was presumably expressed at its native level and in its normal cell type specific pattern of expression. FbxwD-FLAG_3_ was IP’d from the non-ionic detergent solubilized cell lysates using bead-bound anti-FLAG mAb M2, eluted at high pH, and analyzed using nLC MS/MS. Proteins that satisfied the following criteria were examined further: present at a peptide FDR <1%, detected with ≥2 peptides, present at ≥4-fold higher levels in tagged strains vs. untagged strains, and predicted nucleocytoplasmic localization. Data from FbxwD-FLAG_3_ and FbxwD-FLAG_3_UBA_2_ strains were treated as biological replicates as their interactomes were indiscernible. Proteins detected in vegetative stage extracts included the SCF components Skp1 and CulE (Figure 1A), suggesting that a fraction of the FbxwD-FLAG_3_ was engaged in SCF complexes, as well as six additional proteins (Supplementary Table S2A). Two have been named Vwa1 and Vwa2 due to the presence of PFAM sequences predicting the presence of an N-terminal VIT (vault-vault protein inter-alpha- trypsin) domain followed by a vWFA (von Willebrand type-A) domain. These VIT/vWFA interactors are specific to FbxwD as they were not found in pilot studies of a similar analysis of the interactomes of FbxwA-FLAG_3_ and FbxwA-FLAG_3_UBA_2_ (unpublished data), a previously characterized WD40-type FBP that regulates development (Mohanty et al., 2001). A homolog of Vwa1, Vwa6, and Rbx1, a small protein associated with CulE, were also confidently detected but their enrichment was not consistent among trials and thus not statistically significant. During development, Vwa1 and Skp1 remained prominent in the FbxD-FLAG_3_ interactome of slug cells (Figure 1B). Vwa2, CulE, and Vwa3 were confidently detected and also enriched, but Vwa2 identification did not meet the 1%FDR test, and the others did not meet the 4-fold threshold. The remaining interactors seen in vegetative cells were detected near background levels (Supplementary Table S2A). Thus Vwa1 and Vwa2 were chosen for further analysis.

Vwa1 was also previously identified as an interactor of FbxwD in a strain that overexpressed FLAG_3_FbxwD under control of the *cotB* promoter (Sheikh et al., 2015), which drives FLAG_3_FbxwD expression in prespore cells, a cell type in which it is normally not well expressed (see below). Similarly, the interaction also occurred using a mutant form of FLAG_3_FbxwD that does not interact with Skp1, suggesting that the interaction does not involve the SCF complex *per se*. Vwa1, Vwa2 and Vwa3 (described in Supplementary Figure S3) were also previously detected in co-IPs from strains in which endogenous CulE was C-terminally FLAG-tagged, indicating that the interaction persists in SCF complexes (Sheikh et al., 2015). Furthermore, the 3 interactions were enhanced in wild-type vs. *phyA*-KO cells, as also observed with FbxwD, reinforcing the conclusion that the interactions are via FbxwD.

To explore these interactions further, the gene loci for Vwa1 and Vwa2 were separately endogenously tagged with a C-terminal FLAG_3_ tag (Supplementary Figure S6), or Vwa1’s coding sequence was over-expressed with an N-terminal FLAG_3_ tag under control of the semi-constitutive discoidin promotor. Co-IPs of vegetative cells overexpressing FLAG_3_Vwa1 confirmed its interaction with not only FbxwD but also CulE, Rbx1, and an unknown protein annotated as a Zn-dependent alcohol dehydrogenase (Figure 1C). Skp1 was also enriched but not above the 4-fold threshold (Supplementary Table S2B). After development to the slug stage, FbxwD and the alcohol dehydrogenase-like protein were still found, but CulE and Skp1, which were detected, did not meet threshold requirements (Supplementary Table S2B), possibly because FbxwD is naturally expressed at lower levels in slugs (see below). In co-IPs with FLAG_3_Vwa2, FbxwD and CulE were confident interactors, with Skp1 again being less significant (Supplementary Table S2C). In contrast, these interactions were not detected in anti-FLAG co-IPs from strains expressing native levels of Vwa1-FLAG_3_ or Vwa2-FLAG_3_, possibly due to reduced sensitivity at native expression levels. Figure 1D summarizes these observed interactions, and extend previous findings by showing that the FbxwD/Vwa1 and FbxwD/Vwa2 interactions are robust enough to be detected in co-IPs of FbxwD-FLAG_3_ expressed at its native levels, and in the reciprocal direction by pulling down FLAG_3_Vwa1 or FLAG_3_Vwa2. During development, these interactions presumably occur primarily in prestalk cells, the natural cell type of their expression in slugs (see below). The four other Vwa homologues predicted in the *Dictyostelium* genome (Supplementary Figure S3) were not detected, but their presence, or other unknown proteins, might have been below the level of sensitivity of the method.

### Developmental regulation of FbxwD, Vwa1 and Vwa2 expression

**FIGURE 2:**
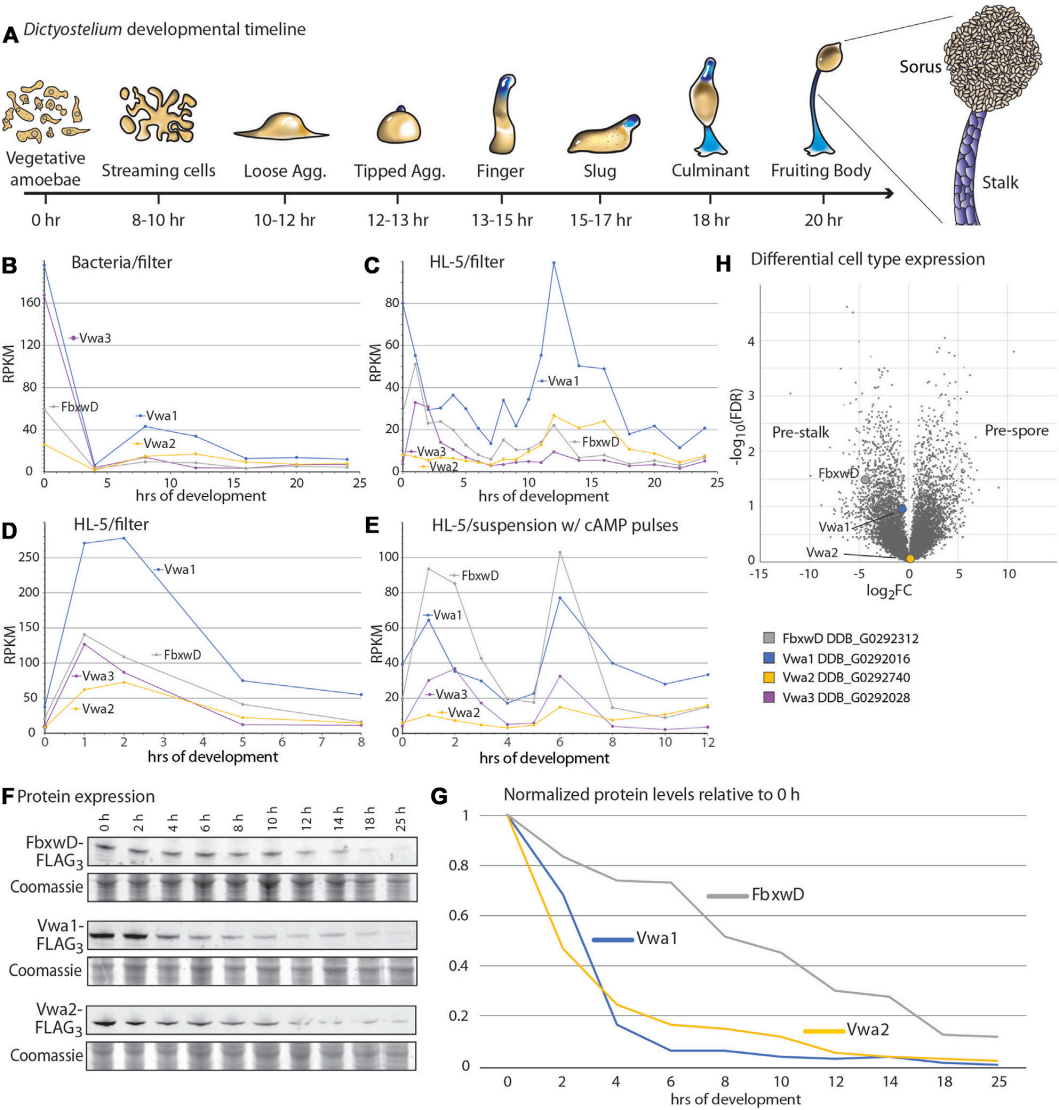
Developmental regulation of FbxwD, Vwa1, Vwa2 and Vwa3 expression. **(A)** Schematic illustration of the starvation-induced developmental cycle of *D. discoideum* at an air-water interface (e.g., filters). Pre-stalk cells and anterior-like cells are colored blue. The life cycle is renewed when spores from a fruiting body germinate to become feeding amoebae (Early et al., 1993). **(B–E)** mRNA expression levels of FbxwD, Vwa1, Vwa2, and Vwa3 during *D. discoideum* development, based on RNAseq studies (Parikh et al., 2010; Rosengarten et al., 2015). Conditions of growth (bacterial or axenic HL-5) and development (filter or suspension) are as indicated. **(F)** Strains whose FbxwD, Vwa1, or Vwa2 loci were C-terminally FLAG_3_-tagged were plated on filters, and whole cell samples were periodically collected for Western blot analysis using anti-FLAG mAb M2. **(G)** Protein expression levels were quantitated by densitometry, normalized to total protein based on Coomassie blue staining, and scaled to 0 h levels. Data are representative of results from two independent trials. **(H)** Volcano plot of slug cell type distribution of FbxwD, Vwa1 and Vwa2 mRNA based on RNAseq studies.

In response to starvation and when plated on a moist surface exposed to atmosphere, *Dictyostelium* cells together execute a developmental program over the course of 20–24 h that results in the formation of fruiting bodies consisting of tens of thousands of aerial spores at the top of stalks (Figure 2A). The significance of the interactions was first examined by assessing whether the proteins are coexpressed during development. An examination of previous transcriptomic data (Parikh et al., 2010; Rosengarten et al., 2015; Santhanam et al., 2015) suggested that, for bacterially grown cells, FbxwD, Vwa1, Vwa2, and Vwa3 were expressed in two waves (Figure 2B): one during growth and the other after starvation induced aggregation on filters (8–12 h) as the cells organize into slugs. Analysis of development of axenically grown cells on HL-5 medium also showed two waves, but the first wave seemed to reach maximal levels only shortly after starvation (Figure 2C). A separate trial confirmed that the first wave for axenically grown cells occurred only just after starvation, either when cells were developed on filters (Figure 2D) or shaken in suspension and pulsed with cAMP (Figure 2E). In contrast, analysis of protein expression, based on Western blot analysis of strains whose endogenous protein loci were FLAG_3_-tagged, confirmed that highest expression levels of the proteins occurred during axenic growth (Figure 2F). During starvation-induced development on filters, the levels of FbxwD, Vwa1 and Vwa2 decreased monotonically (Figure 2G). Vwa1 and Vwa2 declined rapidly over the first 6 h, plateaued for a few hr, possibly the result of the second wave of transcription, before declining further. In comparison, FbxwD protein levels declined more linearly. Significantly, the transcriptome analysis of separated prestalk and prespore cells indicated that FbxwD is approximately 16-fold enriched in prestalk compared to prespore cells (Figure 2H), whereas Vwa1 and Vwa2 appear to be expressed at similar levels between the two cell types. The general correlation in expression levels are consistent with the evidence for the physical interactions, and the differences in cell type expression suggest a possible role for FbxwD in developmental regulation in prestalk cells. Thus the lower expression level is nevertheless expected to be substantial considering that prestalk cells represent <20% of the total cell population.

### Vwa1 stability

**FIGURE 3:**
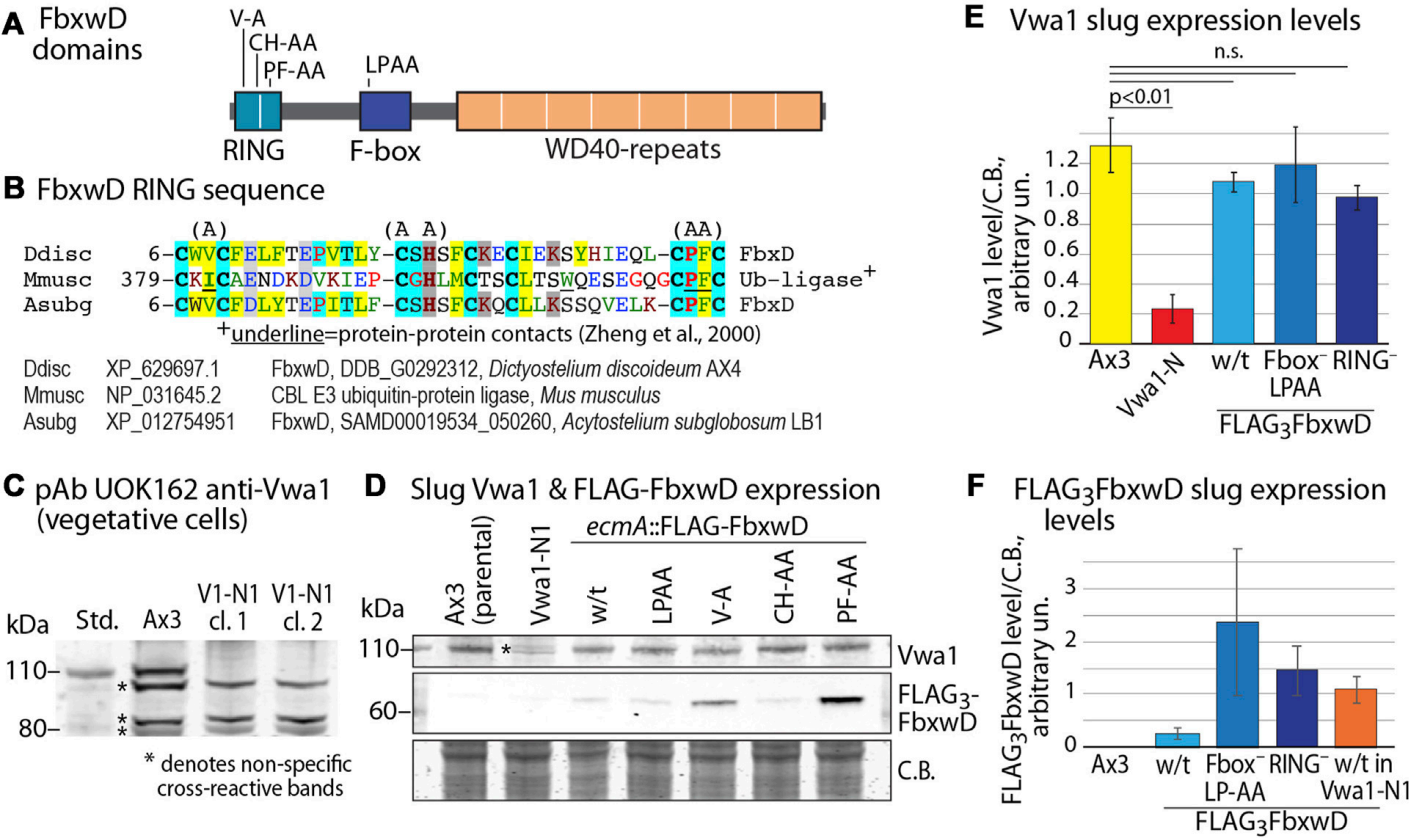
Influence of FbxwD on Vwa1 protein expression levels. **(A)** FbxwD domains and location of point mutations examined. **(B)** Alignment of the putative RING sequence with a homolog from a distantly related social amoeba (*Acytostelium subglobosum*) and from the Cbl E3 Ub ligase for which a structure is available to predict residues (underlined) critical for protein-protein interactions. See Supplementary Figure S9A for an expanded alignment. **(C)** Detection of endogenous Vwa1 in whole cell lysates of vegetative cells based on probing a Western blot with pAb UOK162 (1:200). Ax3 (normal parental) and clones 5 and 11 of strain Vwa1-N1 (V1-N1), whose *vwa1* gene is interrupted in the first (VIT) domain, are compared. **(D)** Slugs expressing native FLAG_3_FbxwD and mutant isoforms under the direction of the pre-stalk (*ecmA*) promotor were probed by Western blotting for expression of Vwa1 using pAb UOK161 and FLAG_3_FbxwD using anti-FLAG mAb M2 (see Supplementary Figures S9B, C for validation with anti-FLAG mAb 12C6c). **(E)**. Densitometric analysis of Vwa1 levels of results shown in panel D, normalized to total protein stained by Coomassie blue (C.B.) (average ± S.D., n = 3). **(F)** Similar analysis of FLAG_3_FbxwD levels (n = 3).

To address the possibility that Vwa1 is a substrate of FbxwD, its level was examined in strains expressing increased levels of native (wild-type) or mutant FbxwD. Since *fbxwD* has resisted disruption (data not shown) and therefore may be essential, a previously described strain in which FLAG_3_FbxD was overexpressed in prespore cells under the control of the *cotB* promoter, and a variant in which the F-box sequence was mutated (LPAA) to reduce interaction with Skp1 and therefore the SCF complex (Sheikh et al., 2015), were examined (see Figure 3A for domain diagram). In addition, 2 new corresponding strains in which FLAG_3_FbxwD was expressed under control of the *ecmA* promotor, which directs expression in prestalk cells, were created. Levels of Vwa1 were monitored using two antisera generated against recombinant full-length Vwa1, pAb UOK161 and pAb UOK162. In a Western blot of an SDS-PAGE gel of vegetative cells, UOK162 labeled a band at the expected *M*_r_ position of 112,000 but several other bands as well (Figure 3C). To verify the identity of this band, the *vwa1* locus was modified by an in-frame insertion of a cassette encoding a FLAG_3_ epitope tag, a stop codon, and a blasticidin resistance gene, in a NsiI site near the middle of the VIT domain (Supplementary Figure S8). The crystal structure of the VIT domain of a human homolog suggested that the VIT remnant would be incapable of folding (see below) and, indeed, the FLAG_3_-tag inserted at the C-terminus of the remnant was not detected. The *M*_r_ 112,000 band was absent in two clones, named Vwa1-N1, confirming its identity as Vwa1 (Figures 3C, E). Similar results were obtained with pAb UOK161 except that interference by non-specific binding was greater; however whereas UOK162 was not suitable for slug cells owing to cross-reacting bands, sufficient resolution was available with UOK161 to allow use in quantitating Vwa1 expression in slug cells (Figures 3D, E). Slugs over-expressing either FbxwD or FbxwD(LPAA) under control of the *ecmA* promotor showed similar levels of Vwa1 as in parental cells (Figure 3F), which did not support the idea that Vwa1 is a substrate.

In additional experiments, vegetative cells in nutrient-free aggregation buffer were incubated in the presence of concentrations of the proteasome inhibitor bortezomib or the protein synthesis inhibitor cycloheximide that were previously shown to be effective (Wang et al., 2011; Boland et al., 2022), but no reproducible differences in accumulation of Vwa1 or Vwa2 were observed (Supplementary Figure S10). However, the possibility that FbxwD mediates Vwa1 turnover in concert with another signal not present under these conditions cannot be excluded.

### FbxwD overexpression inhibits development in a RING and F-box dependent manner

**FIGURE 4:**
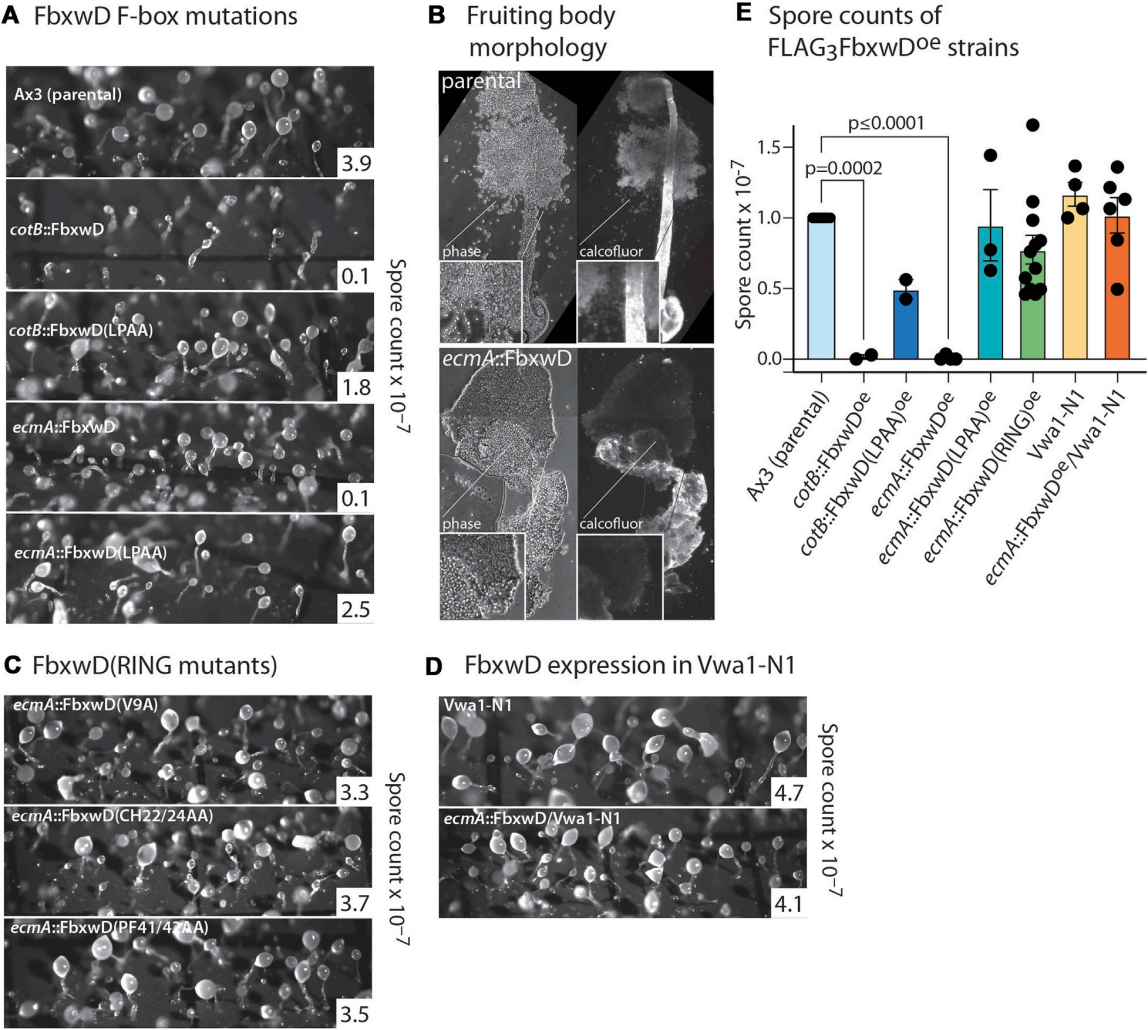
Effects of native and mutant FbxwD on development. **(A)** Cells overexpressing native FLAG_3_FbxwD or its F-box-mutant (LPAA) isoform under the *cotB* or *ecmA* promotors were developed on filters and imaged at their terminal state of development. Representative spore counts are shown. **(B)** Typical fruiting bodies were squashed under a coverslip in Calcofluor White ST and visualized under phase contrast and UV illumination to show stalk cellulose structure and spores of parental and mutant cells. **(C)** Morphology of terminally developed cells expressing RING mutant FbxwD isoforms under the *ecmA* promotor. **(D)** Representative terminal morphologies of Vwa1-N1 cells expressing *ecmA*::FLAG_3_FbxwD. **(E)** Dot plot of average spore counts of *ecmA*::FLAG_3_FbxwD or *cotB*::FLAG_3_FbxwD expression strains, ratioed against counts for the control strain Ax3 included in each trial, from these and other independent experiments (±S.E.M.). RING mutants were pooled together. Average Ax3 spore counts across all experiments were 3.74 × 10^7^ ± 4.22 × 10^6^ (S.E.M.). Differences in mean values were not significant unless indicated, based on a one-way ANOVA analysis of all data.

FbwxD overexpression was previously reported to inhibit developmental timing and prevent successful sporulation when induced under control of the prespore cell-specific *cotB* promotor (Sheikh et al., 2015). Inhibition was dependent on an intact F-box domain as the F-box-mutant (LPAA) expressors sporulated similarly to the parental strain. Since FbxwD expression is highly prestalk cell enriched based on transcript analysis (Figure 2H), cells were instead programmed to over-express FLAG_3_FbxwD or FLAG_3_FbxwD(LPAA) under the control of the *ecmA* promoter to direct expression in this cell type (Figure 4A). Expression was confirmed in slugs (Figure 3F). The FLAG_3_FbxwD^oe^ strain produced fruiting bodies, but its sori contained immature pre-spore cells that lacked the elongated shape and cellulose staining of mature spores. Furthermore, the stalks were enlarged and lacked the regular cylindrical structure (Figure 4B). In comparison, *cotB*:: FLAG_3_FbxwD^oe^ cells exhibited a disruption of terminal morphogenesis (Figure 4A), as well as a block of terminal spore differentiation as previously reported (Sheikh et al., 2015). These results suggest that FbxwD activity level in prestalk cells is critical for orderly terminal differentiation of both stalk and spore cells, and that ectopic expression in prespore cells was even more disruptive.

In contrast, the *ecmA*::FLAG_3_FbxwD(LPAA) mutant expressing cells, which expressed the protein at a similar or higher level than observed for native *ecmA*::FLAG_3_FbxwD (Figure 3F), showed no observable effect on morphogenesis (Figure 4A), and evidence for mild inhibition of terminal spore differentiation was statistically insignificant (Figure 4E). Also, the *cotB*::FLAG_3_FbxwD(LPAA) expressing cells differentiated normally with only a minor sporulation deficit that was also not statistically significant (Figures 4A, E), as previously reported.

To probe the role of the RING-like domain, its sequence was aligned with a list of the most similar sequences available in GenBank (December 2021) (Supplementary Figure S7A). Three sets of point mutations were chosen based on positions that are conserved with related sequences (Figure 3B). The V9A and PF41/42AA substitutions were designed to abrogate the protein-protein contact function of the RING, based on an X-ray crystal structure of a complex of the RING domain of the murine Cbl E3 Ub ligase with an E2 protein (Zheng et al., 2000), and the CH22/24AA mutation was expected to be more disruptive by eliminating Zn^+2^-binding (Figure 3B) (Deshaies and Joazeiro, 2009; Kosztyu et al., 2019; Garcia-Barcena et al., 2020). Cells expressing these FbxwD-RING mutants under control of the *ecmA* promoter were confirmed to express the mutant protein in slugs by Western blotting (Figures 3D, F; Supplementary Figures S9B, C). In preliminary studies, the interactome of all three overexpressed mutants still included Skp1 and Vwa1 (data not shown). As for native FbxwD, overexpression did not affect Vwa1 expression levels (Figures 3D, E).

Overexpression of either of the three RING mutants in prestalk cells failed to obviously affect morphogenesis (Figure 4C), despite their equal or higher expression levels compared to overexpressed native FLAG_3_FbxwD (Figures 3D, F). Spore production was not statistically significantly inhibited (Figure 4E). Given the likely roles of the F-box, RING and WD40-repeat domains in Ub-mediated protein degradation, these findings implicate FbxwD in selective regulation of protein turnover critical for morphogenesis.

### Vwa1 contributes to development

**FIGURE 5:**
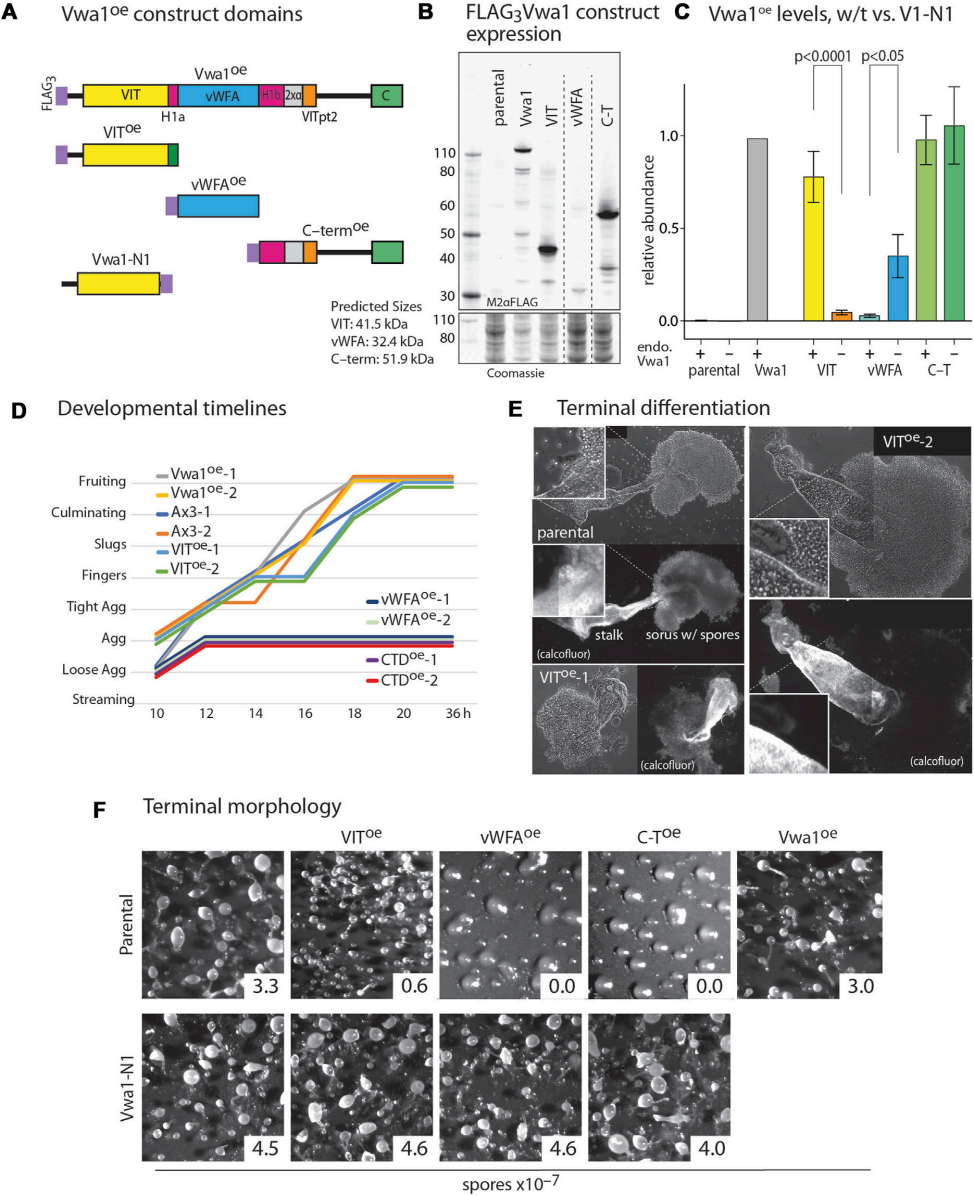
Dominant negative effects of constitutive expression of Vwa1 domains. **(A)** Domain diagram of Vwa1 and its FLAG_3_-tagged constructs, expressed under control of the semi-constitutive discoidin 1γ (*dscC*) promoter. **(B)** Vwa1 and Vwa1 fragment expression was examined in vegetative Ax3 (normal) cells by Western blotting and probing with anti-FLAG mAb M2. Predicted *M*_*r*_ values are listed. **(C)** Summary of expression levels of Vwa1 and Vwa1 fragments in vegetative Ax3 or Vwa1-N1 parental strains, as determined by densitometry of Western blots, after normalization to total protein based on Coomassie blue staining, and scaling to FLAG_3_Vwa1 levels in Ax3 cells. Differences in mean values (±S.E.M.) between pairs (Ax3 vs. Vwa1-N1 parents) were not significant unless indicated, based on a one-way ANOVA analysis of all data (n = 2–15). **(D)** Time course analysis of overexpression strains on filters. **(E)** Representative phase contrast images of fruiting bodies from Ax3 and 2 independent VIT^oe^ strains that developed in plaques on bacterial lawns. Fluorescent imaging with Calcofluor White ST shows cellulose surrounding mature stalk cells and spores. **(F)** Representative terminal morphologies of strains over-expressing truncated or full-length Vwa1 constructs in Ax3 or Vwa1-N1 backgrounds and developed on filters, together with representative spore counts.

To examine the role of Vwa1 in development, a strain from a genome-wide screen that was reported to have an insertion at codon-15 of its *vwa1* ORF (Gruenheit et al., 2021), was analyzed. This strain, referred to as Vwa1-N2, was sporulation-negative. However, this defect was not rescued by complementation of the gene under either the *ecmA* or *cotB* promoter, despite confirmation of expression by Western blotting with pAb UOK161 (Supplementary Figure S7). In comparison, the aforementioned Vwa1-N1 strain, which contains an interruption within the VIT domain of its Vwa1 and is a potential null, appeared to develop normally (Figure 5F; Figure 4E). Since it is unlikely that a residual N-terminal fragment, which was not detected by Western blotting with anti-FLAG mAb M2, would fold into an active domain, we speculate that the GWDI clone possesses additional genetic changes.

The Vwa1 sequence includes a central region predicted to fold into a PFAM vWFA-like Rossman fold, which is found in a wide variety of proteins. The region N-terminal to the predicted vWFA-like domain includes the PFAM VIT region, and C-terminal to the vWFA region lies a less conserved stretch that includes a well conserved CTD sequence (Figure 5A). The schematic diagrams of Figure 5A include subregions that are more fully analyzed below. We reasoned that these N-, middle- and C-terminal domains might fold into separate domains that might have separate functions that could be probed by their independent overexpression. Based on information available at the time, the VIT, vWFA, and C-terminal (C-T) sequences (Supplementary Figures S1B–D) were separately over-expressed with N-terminal FLAG_3_-tags under control of the semi-constitutive discoidin 1γ promoter (Figure 5B). The VIT and C-T regions expressed robustly but the vWFA domain expressed at a lower level similar to that of the full-length construct (Figure 5C). The truncated constructs were immunoprecipitated as for full length Vwa1. No statistically significant interactors were detected for the VIT or vWFA fragments in vegetative and aggregation stage cells (Supplementary Figure S11). The C-terminal Vwa1 fragment co-IPed several heat-shock proteins suggesting partial misfolding (Supplementary Figure S11A). These data indicate that the full-length of Vwa1 is necessary to bind to FbxwD and other SCF proteins.

Full-length Vwa1 over-expression under control of the discoidin 1γ promoter had no significant effect on the timing or morphology of development on filters (Figures 5D, F). VIT overexpression also did not obviously affect morphogenesis, but did result in an 80% reduction in spore differentiation (Figure 5F). Closer inspection of fruiting bodies formed in plaques after consumption of bacteria on nutrient agar surfaces revealed a range of fruiting body morphologies with bloated stalks, predominantly immature prespore cells, and spherical rather than oblong spores (Figure 5E). More dramatically, the vWFA- and C-T overexpressors were not only initially delayed relative to parental cells during development (Figure 5D), but they failed to develop beyond the tight aggregate stage (Figure 5F). A similar block occurred in cleared areas of bacterial plates. To address whether gene expression was similarly blocked, extracts were probed by Western blotting with mAb 83.5, which recognizes a fucose-dependent epitope on several spore coat protein precursors, including SP96 and SP75, that are biomarkers for prespore cell differentiation (West, 2003). All strains expressed similar levels of these prespore markers at 10–12 h (Supplementary Figure S12), indicating molecular progression of this aspect of the prespore development program. In contrast, these overexpression constructs had no apparent effect on growth rate of cells in axenic media (data not shown).

To determine if these trans-dominant negative phenotypes were dependent on full-length Vwa1, the 3 domain constructs were expressed in the Vwa1-N1 strain. Strikingly, development was normal in these strains (Figure 5F), despite the higher or similar levels of expression of the vWFA and C-T fragments. However, the VIT domain was expressed at a much lower level, which could explain why it did not inhibit sporulation.

To further explore a functional interaction between FbxwD and Vwa1, the *ecmA*::FbxwD construct was transfected into Vwa1-N1 cells. Unlike its effect in normal (Ax3) cells, overexpression of FbxwD, as documented by Western blotting (Supplementary Figure S9), failed to inhibit terminal spore differentiation (Figure 4E). Thus, the detrimental effects of both FbxwD overexpression and overexpression of Vwa1 fragments depend on the presence of endogenous full-length Vwa1, even though Vwa1 itself is dispensable for development.

In an effort to detect a possible direct interaction between FbxwD and Vwa1 or Vwa2, expression of FbxwD cDNA sequences optimized for expression in *E. coli* was attempted. However, FbxwD was found only in inclusion bodies, as frequently occurs for FBPs. Similar results when co-expressed with Skp1, or for constructs N-terminally deleted of the RING domain or of the C-terminal WD40-repeat domain (data not shown).

### Evolution of VIT/vWFA containing proteins

Homologs of Vwa1 are present throughout eukaryotic phylogeny. The recent crystal structure of an N- and C-terminally truncated recombinant version of the heavy chain of human ITIH1, a secretory protein that is associated with chondroitin sulfate and hyaluronate during inflammation of mammalian tissues, describes how the sequence is organized into a series of interacting domains (Briggs et al., 2020), as illustrated in Figure 7A. In HsITIH1, the region that includes the PFAM-defined VIT domain belongs to a larger β-sheet sandwich, and also includes a β-strand from a C-terminal region of the protein referred to as VITpt2, The previously annotated PFAM-vWFA domain is inserted within an integrin-associated domain referred to as hybrid-1, with its N-terminal and C-terminal parts referred to as Hyb-1a and Hyb-1b (Figure 7A). Downstream of the Hyb-1b is a triple α-helix bundle that connects to VITpt2. Despite only 16% sequence identity and 35% sequence similarity between these regions of DdVwa1 and HsITIH1 (Supplementary Figure S4), AlphaFold-2 predicts a homologous structural organization of DdVwa1 with some local differences (Figure 7B). A greater sequence similarity is observed with HsVWA5a, which like DdVwa1 is an intracellular protein that is therefore more likely to have related functions: 26% sequence identity and 43% similarity. Furthermore, HsVWA5a has a C-terminal region (CTD) of 101 amino acids that is homologous with a corresponding sequence at the C-terminus of DdVwa1 that is absent from HsITIH1 based both on sequence and AlphaFold predictions (Supplementary Figure S4). Inclusion of this region together with that of DdVwa1 yields an almost identical sequence identity of 24% and similarity of 42%, indicating similar conservation of this region.

**FIGURE 6:**
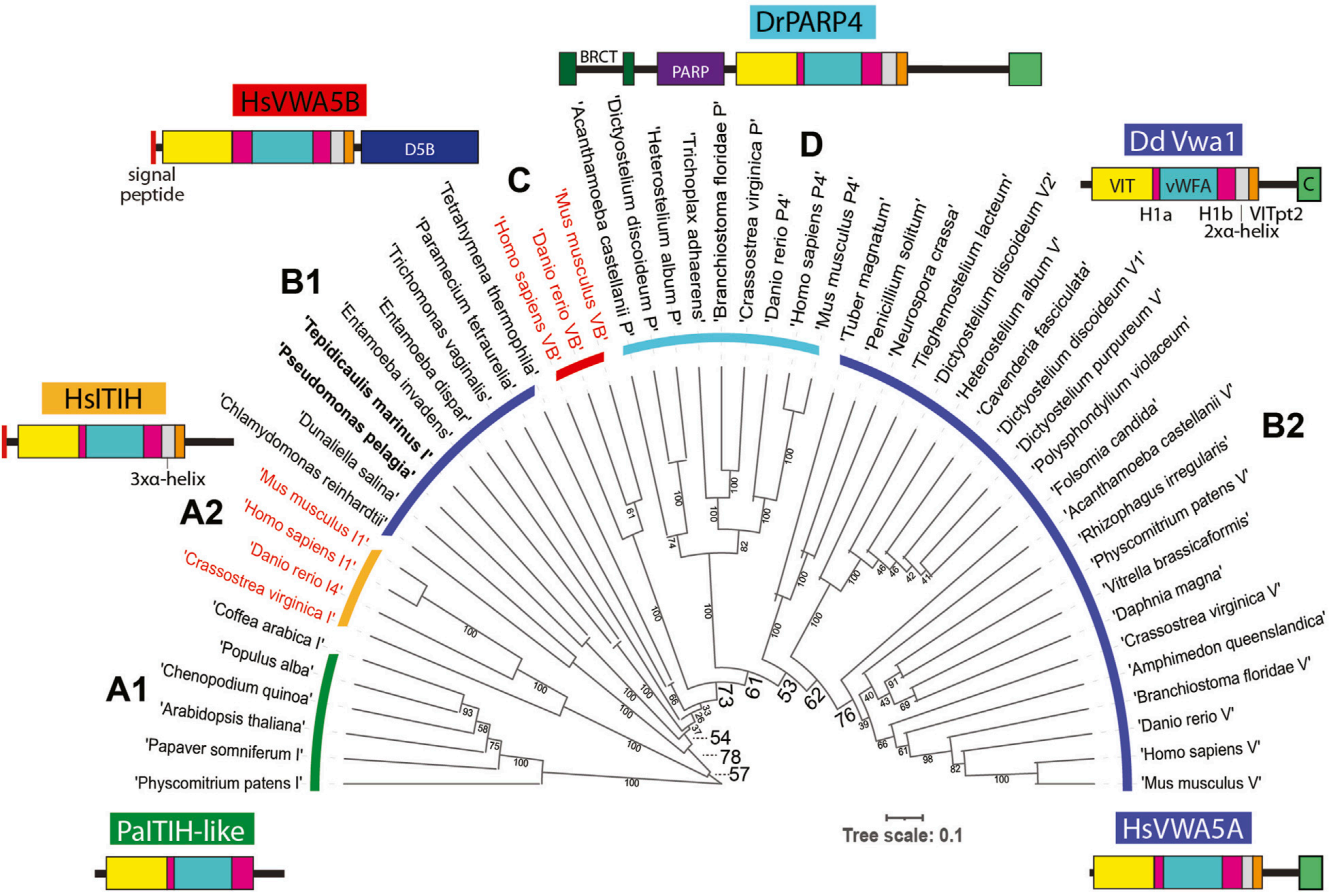
Evolution of Vwa1-like proteins. An alignment of the VIT, Hyb-1a (H1a), vWFA and Hyb-1b (H1b) region amino acid sequences of representative Vwa1-like proteins from throughout phylogeny (Supplementary Figure S5) was subjected to an evolutionary analysis using a maximum likelihood algorithm. Clades are designated based on group similarities (see text). Clades of proteins predicted to have an intracellular localization are marked with a dark blue, cyan or green arc, and those predicted to be extracellular are marked with red or orange arcs; their labels **(A1, A2, B1, C, D, and B2)** are referenced in the text. Bacterial sequences in Clade B1 are in bold. Representative examples of domain architectures are shown for each group.

The *D. discoideum* genome (Fey et al., 2019) harbors 6 additional genes with this sequence organization, each predicted to be intracellular. To assess the evolutionary conservation of the 7 *Dictyostelium* predicted proteins, the vWFA sequence of Vwa1 was used to seed a BLASTp search for similar sequences with Expect values < 0.05. The most similar sequences also contained an upstream VIT region (Whittaker and Hynes, 2002), and the vWFA domain was flanked by Hyb-1a- and Hyb-1b-like sequences. The C-terminal region was more heterogeneous, but a stretch corresponding to the 3xαhelix and VITpt2 regions could be recognized. Many but not all also possessed a CTD-like sequence at the very C-terminus. A preliminary analysis of the evolution of vWFA-like sequences that included a broad range of representatives from throughout eukaryotes, including protist, fungi, plants and animals, using a maximum likelihood method, indicated that VIT-like domains are associated with a monophyletic subset of vWFA domains, as previously suggested using the narrower set of sequences available in 2002 (Whittaker and Hynes, 2002). In addition, an independent BLASTp-based search of VIT-like sequences did not reveal any that lacked an associated vWFA-like sequence. Though separate evolutionary analyses of the VIT- and vWFA-like sequences resolved subgroups of proteins, their evolutionary relationships were ambiguous owing to weak bootstrap values (data not shown). Thus an alignment of the overall VIT-Hyb1a-vWFA-Hyb1b region (Supplementary Figure S5), absent the less conserved 3xαhelix and VITpt2 regions which were difficult to align, was subjected to evolutionary analysis as shown in the unrooted tree in Figure 6.

The sequences grouped into 4 major clades. Clades A1 and A2 are most similar to one another and thus referred to as ITIH like owing to HsITIH1 in clade A2. Interestingly, plant and moss sequences reside in Clade A1 and are likely intracellular proteins (owing to lack of a signal peptide), whereas animal sequences reside in Clade A2 and are extracellular. The combination of Clades B1 and B2 approximates a phylogenetic continuum of related, all intracellular proteins, with bacteria and select protists represented in Clade B1 and other protists, amoebozoa, fungi and animals represented in Clade B2. Emanating from this continuum is Clade C, which comprise a group of animal extracellular proteins, and Clade D, the family of intracellular poly-ADP ribosyl transferases found in amorpheae (inclusive of amoebozoa and animals). The *D. discoideum* Vwa1-5 homologs are more similar to one another than to the separate homologs in other organisms, suggesting lineage specific amplification. A possible scenario that is consistent with this tree is that a homolog was present in the last eukaryotic ancestor, and a gene duplication allowed for diversification of the ITIH- and Vwa-like sequences. The ITIH sequences were perhaps primordial because, in a separate evolutionary analysis (not shown), their vWFA sequences were more similar to vWFA sequences from proteins lacking a VIT domain that probably existed before the group analyzed here acquired the VIT and other domains. However, this interpretation would require a loss of ITIH sequences in protists. Continuing this scenario, a gene duplication in the B-lineage prior to separation of amorpheae allowed for the addition of a PARP domain, and a second later gene duplication allowed for the independent appearance of Vwa5B-like proteins in the extracellular space of animals. However, the relatively weak bootstrap values at critical nodes allow for other histories as well. In any case, proteins of the primary intracellular lineage persist to this day in many protist groups, algae, plants, fungi (though not model yeasts), and animals, and can be predicted to share functions. According to our analysis, DdVwa1 is arguably orthologous to HsVWA5A, and all 5 *D. discoideum* paralogs resulted from lineage specific gene amplifications.

## Discussion

Vwa1 is a highly conserved intracellular multi-domain protein that is broadly expressed across eukaryotes, but whose cellular functions are poorly understood. The findings here implicate a role for Vwa1 in regulating the function of FbxwD, a canonical F-box protein that presumably serves as a substrate receptor in an SCF Ub ligase but also, as suggested by its RING domain, may have a second function possibly in another Ub ligase. The initial clue for this role was the consistent presence of Vwa1 in the interactome of FbxwD, even for mutants of FbxwD that do not bind Skp1, and FbxwD in the interactome of Vwa1 as detected by proteomics analyses of Vwa1 co-IPs. In addition, Vwa1 was an abundant interactor in co-IPs of CulE (a Cul1 equivalent) (Sheikh et al., 2015), and CulE was found in Vwa1 co-IPs, indicating that Vwa1 was associated with SCF complexes themselves and implicating a role in protein polyubiquitination and degradation. Similar findings are shown here for the paralog Vwa2, and evidence exists for involvement of Vwa3 with FbxwD as well. Although we do not exclude the possibility that Vwa1 and paralogs are FbxwD substrates, this does not appear to be a primary relationship owing to no observed effects on Vwa1 abundance when FbxwD was overexpressed, or when cells were treated with proteasome inhibitors (Supplementary Figure S10).

*Dictyostelium* amoebae undergo a complex developmental program when deprived of nutrients, ultimately forming a multicellular fruiting body consisting of thousands of spores perched on top of a cellular stalk (Figure 2A). The sophisticated morphogenetic movements and multiple cell differentiation pathways provide many useful readouts that can help detect and understand the roles of proteins.

Although Vwa1 was found to be dispensable for development, assuming that the Vwa1-N1 strain is a Vwa1 null, expression of its component domains revealed critical involvement in the transition from tight aggregate to the slug stage on through terminal morphogenesis and spore differentiation. When the middle vWFA domain, or the complete C-terminal region downstream from the vWFA domain, were constitutively expressed, development was blocked at the tight aggregate stage (Figure 5) which correlates with an uptick in Vwa1 expression (Figure 2). However, the prespore cell differentiation program was not blocked based on the expression of SP75 and SP96 (Supplementary Figure S12). Similar overexpression of the VIT domain allowed progression to form fruiting bodies, but the stalks cells were disorganized, and prespore cells were markedly reduced in their ability to differentiate into mature spores based on morphology and failure to deposit cellulose into their walls. Remarkably, the vWFA and C-terminal fragment expression constructs were inactive when expressed in Vwa1-N1 cells, and so was the VIT domain with the caveat that it did not express well in our analysis.

Evidence from other proteins implicates specialized roles for discrete Vwa1 domains. Variants of the vWFA domain in other proteins are thought to mediate protein-protein interactions, such as targeting a kinase to its protein substrate (Betapudi and Egelhoff, 2009). The serine and aspartate residues of the Mg^2+^ coordinating metal ion-dependent adhesion site (MIDAS) motif (Whittaker and Hynes, 2002; Springer, 2006) in HsITIH1 are conserved in DdVwa1 and Vwa2 (Supplementary Figures S1, S2), raising the possibility that they too function in homo- or hetero-interactions. HsCLCA1 is an example of a vWFA protein that uses its MIDAS to bind to a specific ligand (Berry and Brett, 2020). In addition, studies on HsITIH1 indicate that it homodimerizes in a metal-dependent manner involving its MIDAS. HsITIH1’s hybrid domain binds the integrin substrate vitronectin a MIDAS independent manner (Briggs et al., 2020). Similarly, the VIT domains of other proteins mediate specific interactions (van Zon et al., 2002; Briggs et al., 2020). In its natural context the C-terminus of HsITIH1 and paralogs are covalently attached to extracellular matrix glycosaminoglycans, but this is itself likely irrelevant to DdVwa1. Thus if DdVwa1 is interpreted to similarly serve as a binding hub connecting multiple proteins together, then selective disruption of one of these interactions while leaving the other(s) intact might be more disruptive than the absence of Vwa1 itself, especially if the Vwa2 or other paralogs might substitute. There is adequate precedent for trans-dominant negative effects of protein fragments for other proteins (Rine et al., 1983; Herskowitz, 1987). Indeed, a recent report showed that expression of the truncated VIT domain of HsITIH5 suppressed tumor growth (Rose et al., 2022). Though we have not obtained evidence for a stable stoichiometric complex, the interactions may be transient or become so upon extract preparation, or Vwa1 may be transiently post-translationally modified (Salier et al., 1996). Though FbxwD lacks a canonical RGD motif or any of the functional derivatives (Mateu et al., 1996) expected to interface with the MIDAS ion, some MIDAS domains exhibit more promiscuous binding by coordinating with an aspartic or glutamic acid on the ligand protein (Luo et al., 2007). Interestingly, biophysical studies have also shown that in integrins, ligand binding allosterically affects the conformation and binding affinity of the MIDAS subregion of its vWFA/hybrid domain. Thus individual domains might also modulate with allosteric regulation.

**FIGURE 7:**
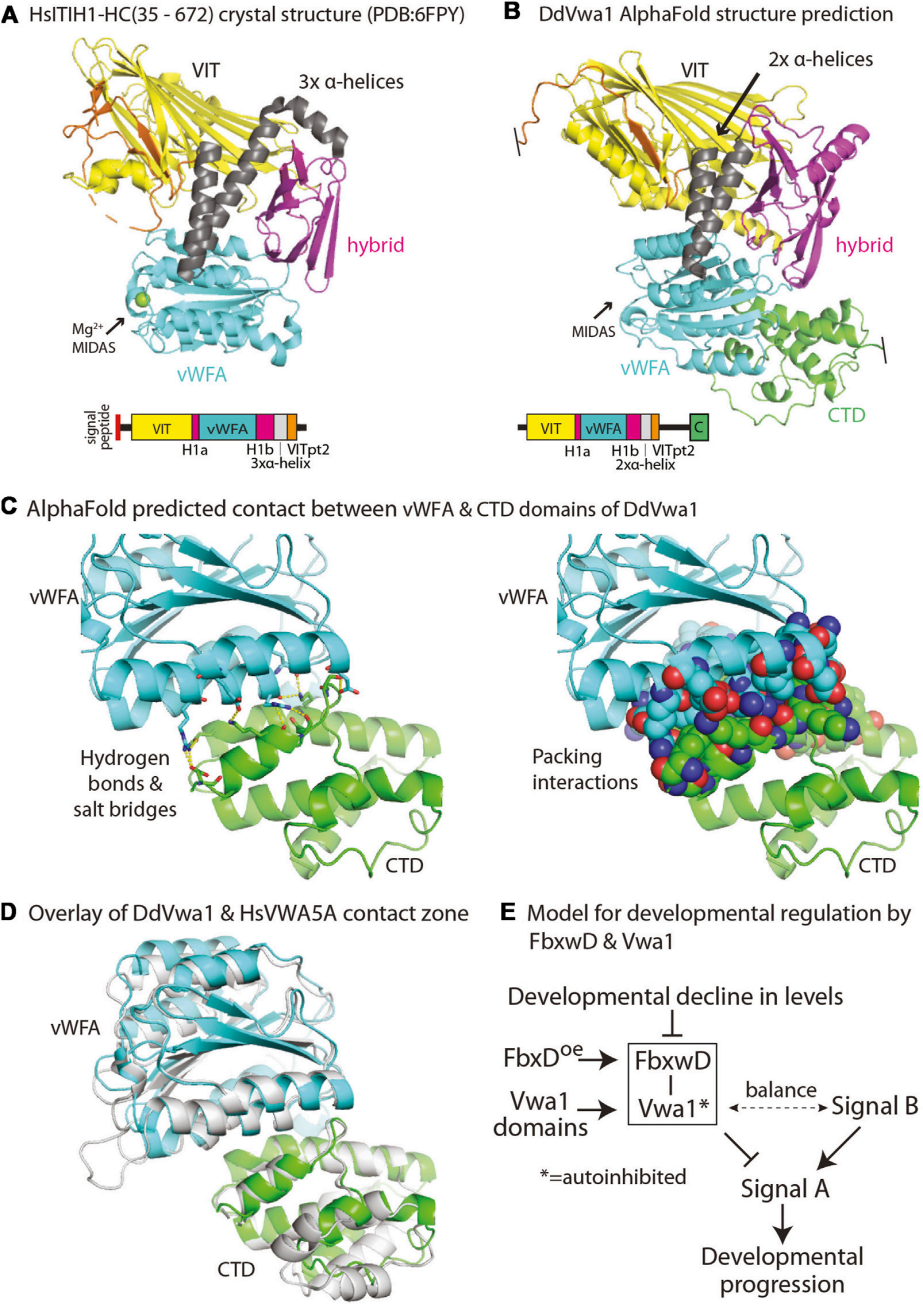
Models for DdVwa1 structure, autoinhibition, and functional interaction with FbxwD. **(A)** Crystal structure of amino acids 35–672 of HsITIH1 (PDB: 6FPY), and **(B)** AlphaFold structure of DdVwa1 are shown as cartoons. The disordered loop connecting VITpt2 and the CTD is not rendered. Their linear peptide domain diagrams are shown below. The structures and the sequence diagrams are colored based on domains described for HsITIH1, except for the CTD which is based on the AlphaFold model. **(C)** Zoom-in of the predicted vWFA (cyan) and CTD (green) domain interaction. (left) Side chains that contribute hydrogen bonds and salt bridges between the two domains are shown in sticks with yellow dashes. (right) Side chains that contribute to packing interactions are shown as spheres. **(D)** Overlay of vWFA and CTD domains of DdVwa1 (cyan and green, respectively) and HsVwa5a (gray). **(E)** Schematic of model for control of developmental progression by the proposed FbxwD/Vwa1 module. The Vwa1 fragments are proposed to relieve a hypothesized autoinhibited state mediated by the CTD. See text (Discussion) for further explanation.

Other possible mechanisms involve perturbation of intramolecular interactions. An interesting mechanism is suggested by modeling of full-length DdVwa1 using AlphaFold2.2. The CTD sequence is linked via long disordered loop and predicted to fold as an α-helical bundle that binds to the vWFA domain, as represented by the structure prediction in Figure 7B. The interface consists of about 905 A^2^ of solvent inaccessible surface area separate from the MIDAS region, and includes seven predicted hydrogen bonds or ionic interactions (Figures 7C, D). This interface appears to be highly conserved, as it is also observed in HsVWA5A (Figure 7E), despite its limited sequence homology (Supplementary Figure S4). This finding is reminiscent of findings in other proteins where interdomain contacts mediate auto-inhibition through domain masking (e.g., Niggli and Rossy, 2008; Antal et al., 2015). Intriguingly, the non-homologous C-terminal of HsITIH1 (and paralogs) must be cleaved prior to covalent linkage to hyaluronate (Salier et al., 1996). Based on these findings and precedents, we suggest that the DdVwa1 fragments expressed in this study compete with specific molecular interactions of Vwa1 and that, since their effects depend on the presence of Vwa1, they act via a binding mechanism that directly or allosterically perturbs homodimerization, or autoinhibition or autoactivation by the CTD.

FbxwD is a high-ranking target for Vwa1 action. Both proteins are highly expressed at the transcriptional level in vegetative cells and decline approximately in parallel during development except for a plateau or peak around the tipped aggregate stage (Figure 2). FbxwD exhibits a strong preference for expression in prestalk cells of the slug, where it has the potential to control terminal differentiation owing to the organizing potential of cells in this group. Vwa1 appears to be more evenly distributed between cell types, so may have other functions in prespore cells. Since FbxwD has not been successfully deleted using several targeting approaches, overexpression was tested to probe its functions. Overexpression in either prestalk or prespore cells strongly interferes with spore differentiation, and ectopic prespore cell expression further interferes with terminal morphogenesis. These effects are reminiscent of effects of VIT-domain overexpression, and do not exclude an effect earlier in development before induction of the cell type specific prespore and prestalk cell promoters tested. Each of these effects depends on the F-box and RING domains as interpreted from effects of their inactivating point mutations. RING domains frequently directly bind Ub-charged E2 proteins in a manner that facilitates transfer of their Ub to a substrate bound to the RING-domain protein (Deshaies and Joazeiro, 2009). In comparison, the F-box domain indirectly binds to the Rbx1 RING-domain via a cullin to activate its own Ub-E2. This duality suggests the possibility of two Ub ligase activities in FbxwD. There are examples of FBPs that depend on protein co-factors for substrate selection (e.g., Hao et al., 2005; Correia et al., 2019). As precedent for this case, human Emi1/Fbx05 possesses a RING-like Zn-finger domain which has been implicated in controlling the rate of polyubiquitination via the F-box domain (Wang and Kirschner, 2013). The dependence of inhibition by FbxwD overexpression on both its RING and F-box domains (Figure 4E), at least within the sensitivity of our assay, suggests that they act synergistically rather than independently.

Strikingly, the negative effect of overexpression in prestalk cells depends on the presence of Vwa1, providing functional evidence for the significance of a direct or indirect physical interaction as detected by co-IPs. The non-essentiality of Vwa1 contrasts with the possible necessity of FbxwD which leads to the hypothesis that Vwa1 primarily acts via modulation of FbxwD.

This mechanism must allow for normal FbxwD activity in the absence of Vwa1 but abnormal activity in the presence of Vwa1 whose function is perturbed by the domain constructs. We suggest that the Vwa1 fragments generate an overactive positive activity or underactive inhibitory activity that balances an unknown opposite regulatory effect on FbxwD, as frequently occurs during developmental or physiological regulation. A model for how this might be coupled to developmental regulation is schematized in Figure 7E. In this scheme, an unknown activating Signal B and an inhibitory FbxwD/Vwa1 module are hypothesized to exert opposing influences on Signal A, which authorizes a given developmental transition. Overexpression of FbxwD forces a stronger inhibitory effect, whereas loss of Vwa1, whose role is natively autoinhibited, has a minimal effect. The minor increase of spore numbers in Vwa1-N1 (Figure 4E) is consistent with leaky autoinhibition of native Vwa1. However, release from autoinhibition as exerted by overexpression of Vwa1 fragments, which disrupt intramolecular domain interactions or possibly homodimerization that mediate autoinhibition, activates the module also inhibiting Signal A. However, the fragments have no effect in the absence of Vwa1. Reduced inhibition owing to the monotonic decline of FbxwD and Vwa1 levels during development allows for developmental progression, but remaining levels are sufficient for implied roles in protein turnover. Interestingly, FbxwD was inhibitory when overexpressed in either prestalk or prespore cells, by interfering with the terminal differentiation of both stalk and spore cells. This is not surprising given the inter-coordination of these two processes during fruiting body formation (e.g., Anjard et al., 2011). It is expected that selective protein turnover would be required to coordinate the numerous parallel processes required for terminal differentiation.

HsVWA5/BCSC-1, which sequence alignments suggest is orthologous to Vwa1 (Supplementary Figure S4), is tumor suppressing and schizophrenia-associated as summarized above. Interestingly, VWA5A has been noted to interact with the SPRY domain containing protein SPSB1, a SOCS-box containing substrate receptor that is analogous to FBPs (Linossi and Nicholson, 2012) in a related class of E3 Cullin-Ring-ligases (Kamura et al., 2004; Nishiya et al., 2011). This and DdVwa1’s physical and functional interactions with DdFbxwD as reported here reinforce the proposed model for this class of proteins in protein degradation.

## Data Availability

The datasets presented in this study can be found in online repositories. The names of the repository/repositories and accession number(s) can be found below: http://www.proteomexchange.org/, XD035633 http://www.proteomexchange.org/, PXD035634.

## Abbreviations

bsr, blasticidin resistance cassette; co-IP, co-immunoprecipitation; CTD, C-terminal domain; FBP, F-box protein; Hyb-1a, Hyb-1b, integrin-related hybrid domain segments; ITIH, inter-alpha-trypsin inhibitor; mAb, monoclonal antibody; pAb, polyclonal antibody; PARP4, Poly(ADP-Ribose) 4; SCF, Skp1/Cul1/F-box protein/Rbx1 complex; SR, substrate receptor; VIT, vault protein inter-alpha- trypsin domain; vWFA, von Willebrand factor-A domain.
