# Supplementary material for: Synergy between a cytoplasmic vWFA/VIT protein and a WD40-repeat F-box protein controls development in Dictyostelium
Source: Front Cell Dev Biol. 2023 Sep 14;11:1259844. doi: 10.3389/fcell.2023.1259844 (PMC10539598; doi:10.3389/fcell.2023.1259844)
Supplement: Supplementary file 1 [file DataSheet1.PDF]

Synergy between a cytoplasmic vWFA/VIT protein and a WD40-repeat F-box protein controls development in *Dictyostelium*

Andrew W. Boland[1,2], Elisabet Gas-Pascual[1,2,3], Hanke van der Wel[1], Hyun W. Kim[1], Christopher M. West[1,2,3]

[1]Dept. of Biochemistry & Molecular Biology, [2]Complex Carbohydrate Research Center, [3]Center for Tropical and Emerging Global Diseases, University of Georgia, Athens, GA 30602

Table of Contents

Table S1. Oligonucleotides employed in this study.

Table.S2. DdFbxwD, DdVwa1, and DdVwa2 interactomes.

Fig. S1. DdVwa1 DNA and protein sequence.

Fig. S2. DdVwa2 DNA and protein sequence.

Fig. S3. Vwa1-like proteins in *D. discoideum*.

Fig. S4. Alignment of DdVwa1, HsITIH1, and HsVwa5 sequences.

Fig. S5. Alignment of Vwa1-like sequences throughout phylogeny.

Fig. S6. Epitope tagging of Dd*vwa1* and Dd*vwa2* loci and Western blot validation.

Fig. S7. Complementation of the GWDI Dd*vwa1*-disruption strain.

Fig. S8. Dd*vwa1* disruption strategy and validation.

Fig. S9. Expression of DdFbxwD constructs.

Fig. S10. DdVwa1 and DdVwa2 stability.

Fig. S11. Interactomes of over-expressed DdVwa1 domains.

Fig. S12. Development of DdVwa1 mutant strains.

Fig. S13. Sequence of the $FLAG_3UBA_2$ cassette.

Table S1. Oligonucleotides employed in this study

Primer Name	Sequence
**FbxwD-V9A RING point mutations**	
DdFbxD-V9A-S	5'-GG*GCATGC*TTTGAATTGTTCACTGAACCAG *Sph1* substitution in red
DdFbxD-V9A-AS	5'-A*GCATGC*CCAACAGTTATAGTCATATGACAT *Sph1*
**FbxwD-CH22/24AA RING point mutations**	
DdFbxD-CH22/24AA-S	5'-T*ATGCAT*CAGCTTCATTTTGTAAAGAATGTATAGAGAAATCATATCATATCG *NsiI*
DdFbxD-CH22/24AA-AS	5'-TGAAGCTG*ATGCAT*ATAATGTCACTGGTTCAGTGAACAATTCAAAG *NsiI*
**FbxwD-PF41/42AA RING point mutations**	
DdFbx-DPF41/42AA-S	5'-TA*TGCGCA*GCTTGCAGAAAAGAAATTCAAATGCCACTAC *Fsp1*
DdFbxD-PF41/42AA-AS	5'-CAAGC*TGCGCA*TAATTGTTCGATATGATATGATTTCTCTATAC *Fsp1*
**Vwa1 endogenous tagging**	
endo_pVS 3' (2)_	5'-TTATTGTTGTTGTTGTTGTTTTAAAATTTGTTGTTGAGTATATTCCCATATTACAATTGAACCAGATACATGAACATTTAAAC
endo_pVS c_term (1)	5'-GAGAGTTTCAGCATCCTCACTTGCAG
5'VWF1_c_t 2 (4)	5'-tgcaagtgaggatgctgaaactctcGTGTTGAAAGTTATGAAAGTGACG
3'VWF1_c_t 2 (3)	5'-GCTGCCGCTGCCAACTAATGATTTAGCGTTTGATAAAAATTT*ATCGAT*AC
VWF_FLAG_BSR (5)	5'-taaatcattagttGGCAGCGGCAGCAGATCT
3'BSR_VWF_non (6)	5'-aaactgaattattCTGCAGGAATTAACCATGCG
5'VWF non_code_b (7)	5'-ttaattcctgcagAATAATTCAGTTTATGAAAAAGAAAAGG
5'VWF n_c_v (8)	5'-tttaaaacaacaacaacaacaataaGGAAGTGGTATAATTTCAAAAG
**Vwa1 endogenous tagging confirmation**	
VWA1 3.UTR con_sq (14)	5'-CAAGGTTTATAGAATTACCAAAATAATTTGTACATTTACCGA
pVS tag non-c seq F V2 (9)	5'-CCGAAAGCTCGGATCTGATATCATAACTTC
pVS tag C-t seq R V2 (11)	5′-aaattttgttgttgttgttgttattCCTTCACCATATTAAAGTTTTC
VWA1 c_t conf_seq (13)	5'-CATCTTCATCCCAAAAGAAGAAAAAAGAAGTTAGTAG
3xFLAG_C-term (10)	5′-GTGCTTCAAAAATATCATTTAAACCACCACCG
BSR_3'targeting (12)	5′-AAGATAAAGCTGACCCGAAAGCTC
**Vwa1-disruption**	
VWA1_KO_PvuI_F (1)	5'-ata*cgatcg*TTTAGTAGTATAACAAGTAAATTATCAGC *PvuI*
VWA1_KO_PvuI_R (2)	5'-tt*cgatcg*TTAAACTAATGATTTAGCGTTTG *PvuI*
VWA1_KO_FGBSR (3)	5'-atatgttcccatcttatgcaGGCAGCGGCAGCAGATCT
VWA1_KO_BSR_R (4)	5'-tatagttgaaattgaatgcaCTGCAGGAATTAACCATGCGGC
**Vwa1-disruption screening**	
pMini sequencing S (5)	5'-ACCTGCCAACCAAAGCGAGAAC
pMiniT sequencing AS (6)	5'-TCAGGGTTATTGTCTCATGAGCG
pVS tag C-t seq R V2 (7)	5'-GTGCTTCAAAAATATCATTTAAACCACCACCG
VWA1_FLAG_KO_val1_F (8)	5'-GAGCTCGAAATTTATTTCAAGCATTTTCTTTTTTTTTTCCC
**Vwa1 expression**	
VWA1_OE_NcoI	5'-TA*CCATGG*aTTTAGTAGTATAACAAGTAAATTATCAGC *NcoI*
VWA1_OE_BamHI	5'-TA*GGATCC*TTAAACTAATGATTTAGCGTTTG *BamHI*
VWA1_vitOE_F	5'-tt*GGATCC*TTTAGTAGTATAACAAGTAAATTATCAGCAATTTCAGG *BamHI*
VWA1_vitOE_R	5'ACTA*GGATCC*ACTCTTTTGATAAACTTCATCTGGATTAACATTCTTG *BamHI*
VWA1_vwaOE_F	5'-AGG*GGATCC*GAATTTATTTTCTTAATTGATTGTTCTGGTTCAATGAGTGG *BamHI*
VWA1_vwaOE_R	5'-GAA*GGATCC*ATTTGATGGTATCATTGAATAGATCATCATTCTCTCTTG *BamHI*
VWA1_ctermOE_F	5'-GCAA*GGATCC*CAAACCAATCAAGATATCATCAATGCC *BamHI*
**Vwa1 expression plasmid sequencing**	
pV3 sequencing primer S	5'-GGTACCAAAAAAATGTCTGATCA
pV3 sequencing primer AS	5'-GAGCTCTGATCATTAGGATCC
Vwa1_OE_seq_F	5'-CAATTACATTATTGTCTTCATCGTTATATGTTCCC
VWA1_OE_seq_R	5'-CGTTTAGTAGCAGTAGATGGGGAGG
VWA1_OE_seq_R2	5'-CACCAATACCATAGGTGAAGATACGAGTTG
VWA1 c_t conf_seq	5'-CATCTTCATCCCAAAAGAAGAAAAAAGAAGTTAGTAG
pVS_bamHI_Val	5'-GGTTTAAATGATATTTTTGAAGCACAAAAAATTGAATGG
pVS_V1vit_conR	5'-CGATGAAGACAATAATGTAATTGACCATCGG
**Vwa2 expression**	
VWA2_OE_NcoI	5'-AC*CCATGG*aATAAAAAATTTAATTTCAGCATTTTCACAAGG *NcoI*
VWA2_OE_BamHI	5'-AT*GGATCC*TTATACATTTGATTTTGCAAGTTCTAAAAATTGATC *BamHI*
**Vwa2 expression plasmid sequencing**	
pV3 sequencing primer S	5'-GGTACCAAAAAAATGTCTGATCA

pV3 sequencing primer AS	5'-GAGCTCTGATCATTAGGATCC
VWA2_OE_seq_F	5'-CTATTACATCGTTACTGTTTTCCACAATCGTCA
VWA2_OE_seq_R2	5'-CAGAGACAGCACCATCAGTAAGAATG
VWA2 c_term conf_seq V2	5'-GAACCAACACTTTCAAATGTATCTTTTGATTGGAGTC
VWA2_OE_seq_R	5'-CTGAAGTGGTGAATTAAAATTAACAGCAAAAG

Vwa2 endogenous tagging

endo_pVS 3 (2)_	5'-TTATTGTTGTTGTTGTTGTTTTAAAATTTGTTGTTGAGTATATTCCCATATTACAATTGAACCAGATACATGAACATTTAAAC
endo_pVS c_term (1)	5'-GAGAGTTTCAGCATCCTCACTTGCAG
VWF2 3_c_t (3)	5'-ataatcagatctTACATTTGATTTTGCAAGTTCTA
VWF2 5_c_t (4)	5'-gaggatgctgaaactctcGAACTCGATAATGATATTTCTAATAAC
V2_FG_BSR (5)	5'-cttgcaaaatcaaatgtaAGATCTGATTATAAGGATGATGA
3_BSR V2_non (6)	5'-cttattctcaaaccCTGCAGGAATTAACCATG
5_V2 non_code_b (7)	5'-gttaattcctgcagGGTTTGAGAATAAGAAATATTATTTATC
3_V2 n_c_v (8)	5'-taaaacaacaacaacaacaataaCCAATACCATTACCTGCTG

Vwa2 endogenous tagging confirmation and sequencing

pVS tag C-term seq F (9)	5'-CTTTACGGATGATCCCTGTAATCCGGG
pVS tag C-term seq R (11)	5'-GCTCTGATCATTAGGATCC
VWA2 c_term conf (13)	5'-GCAATGGTAAAAGTACAACTATAATTCAATCACCTACTC
VWA2 3.UTR conf (14)	5'-CATCCAATAATATATTTACTGCAGCTTCAGCCATATC
3xFLAG_C-term (10)	5′-GTGCTTCAAAAATATCATTTAAACCACCACCG
BSR_3'targeting (12)	5′-AAGATAAAGCTGACCCGAAAGCTC

FbxwA endogenous tagging

FbxA5'-S	5′-AA*GCGCGC*CAAAGCTGTAAAGAGTGAGGGTAATTCAG *BssHII*
FbxA5'-AS	5′-AA*AGATCT*AAAGTCCCATCTCAAAACCAATCCA *BglII*
FbxA3'-S	5′-AT*CTGCAG*TACCCCAAACCTCAACAACATAAGG *PstI*
FbxA3'-AS	5′-TT*CAGCTG*TTTAATGGAAGAGGAAGAAGAAATGACAG *PvuII*

Table S2. Summary of proteomic analyses of co-IPs from strains expressing epitope tagged FbxwD, Vwa1, or Vwa2.

Proteins were identified by nLC-MS/MS in an Orbitrap mass spectrometer, and identified and quantitated based on peptide spectral counts. **(A)** Cells whose FbxwD locus was C-terminally $FLAG_3$-tagged. **(B)** Cells overexpressing $FLAG_3$Vwa1 under control of a semi-constitutive discoidin promoter. **(C)** Similarly for $FLAG_3$Vwa2. Protein features, FDR for identification, and enrichment in anti-FLAG co-IPs from tagged relative to the untagged parental strain are indicated. Proteins identified at an FDR of <1%, and at an enrichment ratio of greater than 4 at a Wilcoxon p-value of <0.05, and are classified as significant interactors. Common background proteins such as mitochondrial and ribosomal proteins, actin and actin binding proteins, secreted proteins, and discoidins were excluded from the list. Proteins that were identified as putative candidates in one condition that did not meet the criteria threshold are highlighted in gray.

Table S2A. FbxwD-FLAG3

Accession (dictybase.org)	MW [kDa]	Veg. Score Sequest HT	Slug Score Sequest HT	Description (dictybase.org)	FbxwD-FLAG interactome, endogenous tag: Vegetative (proliferating stage): Protein FDR Confidence	Specificity anti-myc	p-value	Wilcoxon p-value	Slug stage: FDR Confidence	Specificity anti-myc	p-value	Wilcoxon p-value	
DDB0306754	60.5	688	1486	FbxwD	<0.01	75.7	5.65E-03	0.014	<0.01	34.2	2.00E-04	2.39E-01	F-box protein
DDB0306928	87.8	525	1538	DDB_G0279003	<0.01	4.6	3.96E-02	0.100	<0.01	1.1	9.31E-01	3.62E-01	RING/Retinoblastoma-binding protein 6
DDB0191107	18.7	487	184	Skp1	<0.01	79.5	2.93E-02	0.014	<0.01	4.3	5.25E-02	1.55E-03	SCF member
DDB0266744	87.1	369	424	CulE	<0.01	54.2	2.52E-02	0.014	<0.01	2.0	1.66E-01	1.48E-02	SCF member
DDB0306747	102.3	316	490	Vwa1	<0.01	54.0	2.29E-02	0.014	<0.01	15.8	9.32E-03	7.77E-04	
DDB0238133	101.8	60	45	Vwa2	<0.01	>200	2.05E-04	0.014	0.01-0.05	70.3	8.13E-02	9.32E-03	
DDB0304493	45.4	54	600	UBA domain	<0.01	>200	2.29E-02	0.171	<0.01	1.1	9.20E-01	4.17E-01	FbxwD tag from Rad23
DDB0191243	31.1	49	ND	AcpB	<0.01	>200	6.88E-03	0.029	ND				actin capping protein B
DDB0238863	31	47	ND	Dph5	<0.01	143	9.57E-03	0.100	ND				diphthamide methyltransferase
DDB0349338	16.2	26	775	DDB_G0281243	<0.01	7.4	3.97E-02	0.171	<0.01	0.7	3.47E-01	4.66E-02	RNA binding motif

ND=not detected

Indicates identification below interactor threshold

Table S2B. FLAG3-Vwa1(oe)

FLAG-Vwa1 interactome, overexpressed consructs — Vegetative (proliferating stage)

Accession (dictybase.org)	MW [kDa]	Veg. Score Sequest HT	Description (dictybase.org)	Full-length: Protein FDR Confidence	Specificity anti-myc	p-value	Wilcoxon p-value	VIT-only: Specificity anti-myc	p-value	Wilcoxon p-value	vWFA-only: Specificity anti-myc	p-value	Wilcoxon p-value	C-terminus-only: Specificity anti-myc	p-value	Wilcoxon p-value
DDB0306747	102.3	92640	Vwa1	<0.01	83.5	5.84E-11	2.06E-05	26.1	8.18E-08	2.06E-05	1.5	1.37E-01	5.68E-02	158.0	2.30E-11	2.06E-05
DDB0306754	60.5	391	FbxwD	0.01-0.05	43.0	1.44E-06	2.06E-05	1.5	4.92E-01	1.29E-01	1.6	2.23E-01	8.08E-02	2.3	6.89E-02	3.13E-02
DDB0266744	87.1	461	CulE	0.01-0.05	94.9	4.74E-05	2.06E-05	0.6	3.42E-01	2.52E-02	0.6	3.28E-01	2.00E-02	0.6	1.77E-01	9.51E-02
DDB0191107	18.7	306	Skp1	<0.01	2.6	5.50E-02	5.10E-03	0.43	0.18	NC	0.46	0.15	NC	0.4	0.27	NC
DDB0231276	12.1	97	Rbx1	0.01-0.05	7.9	1.30E-02	1.20E-04	ND			ND			ND		
DDB0238199	36.2	102	DDB_G0268322*	0.01-0.05	>200	2.22E-16	2.06E-05	1.0	4.43E-01	9.51E-02	1.0	8.03E-01	2.73E-01	>200	0.00E+00	2.06E-05
DDB0191168	70.4	26865	HspB	<0.01	3.4	1.05E-04	2.06E-05	3.9	2.44E-03	2.06E-05	1.1	5.87E-01	3.33E-01	9.3	1.96E-07	2.06E-05
DDB0185047	69.7	23557	HspE-1	<0.01	1.5	4.19E-02	4.70E-02	2.2	1.23E-02	1.22E-02	0.9	7.75E-01	4.66E-01	5.5	9.92E-07	2.47E-04
DDB0233663	72.5	5243	Grp78	<0.01	2.5	4.85E-05	2.06E-05	1.4	4.00E-02	2.82E-03	0.9	9.06E-01	3.33E-01	6.8	7.58E-10	2.06E-05
DDB0191276	86	662	HspH	<0.01	1.4	3.63E-01	6.80E-02	5.8	3.36E-01	3.33E-01	1.3	9.51E-01	4.66E-01	35.2	8.78E-06	2.06E-05

Aggregation/Slug stage

Accession (dictybase.org)	MW [kDa]	Agg Score Sequest HT	Description (dictybase.org)	Full-length: Protein FDR Confidence	Specificity anti-myc	p-value	Wilcoxon p-value	VIT-only: Specificity anti-myc	p-value	Wilcoxon p-value	vWFA-only: Specificity anti-myc	p-value	Wilcoxon p-value	C-terminus-only: Specificity anti-myc	p-value	Wilcoxon p-value
DDB0306747	102.3	14865	Vwa1	<0.01	143.2	2.60E-04	1.08E-03	15.8	1.14E-03	2.96E-02	0.9	6.52E-01	2.94E-01	32.8	5.03E-03	5.83E-04
DDB0306754	60.5	130	FbxwD	0.01-0.05	24.7	7.05E-03	1.08E-03	0.2	3.50E-01	9.99E-03	1.4	4.85E-01	1.97E-01	0.6	6.61E-01	1.83E-01
DDB0266744	87.1	36	CulE	0.01-0.05	>200	6.97E-02	8.98E-02	>200	2.27E-01	2.96E-02	>200	2.41E-01	4.65E-02	>200	5.34E-01	2.67E-01
DDB0191107	18.7	57	Skp1	0.01-0.05	1.35	0.4	NC	0.27	0.14	NC	2	0.31	NC	0.45	0.49	NC
DDB0231276	12.1	ND	Rbx1	ND				ND			ND			ND		
DDB0238199	36.2	20	DDB_G0268322*	0.01-0.05	>200	8.90E-03	NC	ND			ND			ND		
DDB0191168	70.4	4481	HspB	<0.01	2.9	1.53E-01	8.98E-02	2.7	5.72E-02	2.96E-02	1.6	3.73E-01	8.98E-02	2.5	1.90E-01	1.17E-01
DDB0185047	69.7	3317	HspE-1	<0.01	1.4	4.12E-01	1.97E-01	1.7	1.43E-01	9.99E-03	0.9	9.10E-01	3.50E-01	1.6	5.48E-01	2.23E-01
DDB0233663	72.5	1789	Grp78	<0.01	2.0	2.10E-01	8.98E-02	1.0	9.20E-01	9.99E-03	1.1	8.60E-01	5.31E-01	1.3	6.59E-01	4.18E-01
DDB0191276	86	ND	HspH	ND				ND			ND			ND		

* annotated as Zn-alcohol dehydrogenase   ND = not detected   NC= not calculated   Indicates identification below interactor threshold

Table S2C. FLAG3-Vwa2(oe)

FLAG-Vwa2 interactome, overexpressed — Vegetative (proliferating stage)

Accession (dictybase.org)		MW [kDa]	Veg. Score Sequest HT	Description	Protein FDR Confidence	Specificity anti-myc	p-value	Wilcoxon p-value	
DDB0238133	DDB_G0292740	101.8	2094	Vwa2	<0.01	>200	1.50E-05	<0.05	
DDB0306754	DDB_G0292312	60.5	18	FbxwD	0.01-0.05	>200	5.50E-07	<0.05	
DDB0266744	DDB_G0278991	87.1	8	CulE	0.01-0.05	85.30	6.80E-02	<0.05	
DDB0191107	DDB_G0269230	18.7	13	Skp1	<0.01	1.31	3.34E-01	<0.05	
DDB0232369	DDB_G0282997	103.8	4	Rad54	0.01-0.05	>200	1.08E-01	<0.05	Snf2 helicase-like
DDB0304711	DDB_G0282997	213	4	Rnf160	0.01-0.05	>200	8.70E-02	<0.05	Ring finger containing
DDB0348542	DDB_G0275163	36.2	100	unk.	<0.01	2.75	1.70E-04	<.0.05	unknown
DDB0191168	DDB_G0269144	70.4	298	HspB	<0.01	2.66	1.05E-04	<0.05	

Slug stage

Accession (dictybase.org)		MW [kDa]	Agg Score Sequest HT	Description (dictybase.org)	Protein FDR Confidence	Specificity anti-myc	p-value	Wilcoxon p-value	
DDB0238133	DDB_G0292740	101.8	1090	Vwa2	<0.01	>200	1.60E-03	<0.05	
DDB0306754	DDB_G0292312	60.5	3	FbxwD	0.01-0.05	>200	2.30E-04	<0.05	
DDB0266744	DDB_G0278991	87.1	ND	CulE		ND			
DDB0191107	DDB_G0269230	18.7	2	Skp1	0.01-0.05	2.24	0.31	0.35	
DDB0235357	DDB_G0276341	60.7	12	PccB	<0.01	4.67	2.40E-03	<0.05	propionyl-CoA carboxylase
DDB0348542	DDB_G0275163	36.2	7	unk	0.01-0.05	>200	8.90E-03	<0.05	
DDB0191168	DDB_G0269144	70.4	298	HspB	<0.01	5.77	2.30E-05	<0.05	
DDB0185040	DDB_G0276141	39.9	12	Pdl1	<0.01	4.56	3.60E-03	<0.05	Protein disulfide isomerase

ND = not detected   Indicates identification below interactor threshold

Figure S1. DdVwa1 nucleotide and amino acid sequences.

(A) The sequence information for DdVwa1 is from dictyBase DDB_G0292016 (chromosome: 6 position 1027245-1030001) and Uniprot Q54DV3, and is consistent with all DNA and amino acid sequence data obtained in this study. Untranslated DNA of this intronless gene is in gray. Domains are named and highlighted according to sequence homology to that of HsITIH1 heavy chain (**Figure S4**), and structure homology based on comparison of the AlphaFold-2 predicted structure (**Figure 7**) to that of HsITIH1 (PDB 6FPY), as indicated. Oligonucleotides and relevant restriction enzyme sites are included; primer nt in red denote non-homologous sequences; primer nt in italics denote restriction enzyme sites; potential nuclear localization sequence is in blue font.

(B) Vwa1 overexpression construct sequence

(C) Vwa1 VIT-domain overexpression construct sequence

(D) Vwa1 vWFA-domain overexpression construct sequence

(E) Vwa1 C-terminal region overexpression construct sequence

(F) Vwa1-N1 open reading frame

A. Vwa1 locus

```
TTATTTAAAATCAAAAATAATTTTTTTTTTTTTTTTTTTATAATCTCATTTTTTTTTATT
TTTTTTTAAAAACAAATAAAAAACAAATATATAAATATAAAAAAAAAAAAAAAAAAAAAA
                                                  5'-taccat   VWA1_OE_NcoI
                                                   5'-ttgga   VWA1_vitOE_F

M  F  S  S  I  T  S  K  L  S  A  I  S  G  G  S  S  K  N  D   20
ATGTTTAGTAGTATAACAAGTAAATTATCAGCAATTTCAGGTGGATCAAGTAAAAATGAT
ggaTTTAGTAGTATAACAAGTAAATTATCAGC             TGA     GWDI_133_C_2 insert stop codon
tccTTTAGTAGTATAACAAGTAAATTATCAGCAATTTCAGG

Y  K  E  A  N  Y  Y  N  Y  F  R  I  L  E  K  K  Q  T  E  E   40
TATAAAGAAGCAAATTATTATAATTATTTTAGAATTTTAGAAAAGAAACAAACTGAAGAG

I  A  R  K  R  C  G  L  Y  S  L  K  N  H  N  N  V  F  V  L   60 VIT
ATTGCAAGAAAAAGATGTGGTTTATATAGTTTAAAAAATCATAATAATGTATTTGTTTTA

K  E  F  S  I  E  T  E  I  N  D  C  S  S  T  S  I  W  T  Q   80
AAAGAGTTTTCAATTGAAACTGAAATTAATGATTGTAGTTCAACATCAATTTGGACACAA

S  Y  S  N  D  S  N  T  P  V  E  A  K  Y  Q  L  P  L  H  P   100
AGCTATTCAAATGATTCAAATACACCAGTCGAGGCAAAGTATCAATTACCATTACATCCA

T  S  V  V  S  N  F  Q  I  E  Y  Q  G  K  V  I  Q  G  K  I   120
ACATCGGTTGTATCAAATTTTCAAATTGAATATCAAGGTAAAGTAATTCAAGGTAAAATC

K  E  K  E  K  A  L  E  K  Y  N  D  A  I  A  S  G  G  Q  A   140
AAAGAGAAAGAGAAAGCATTAGAGAAATACAATGATGCAATTGCAAGTGGTGGTCAAGCA

F  M  A  T  K  S  D  D  G  Y  F  N  L  T  L  G  N  L  P  P   160
TTTATGGCAACAAAATCAGATGACGGTTATTTCAATTTAACACTTGGTAATTTACCACCA

K  E  N  V  K  V  R  V  V  I  S  S  E  L  G  T  H  T  D  G   180
AAAGAGAATGTTAAAGTTAGAGTTGTAATCTCATCAGAGTTGGGTACACATACCGATGGT

Q  L  H  Y  C  L  H  R  Y  M  F  P  S  Y  A  F  N  F  N  Y   200
CAATTACATTATTGTCTTCATCGTTATATGTTCCCATCTTATGCATTCAATTTCAACTAT    NsiI insertion site
                         5'-atatgttcccatcttatgcaGGCAGCGGCAGCAGATCT   VWA1_KO_FGBSR
                   3'-CGGCGTACCAATTAAGGACGTCacgtaagttaaagttgatat     VWA1_KO_BSR_R

N  V  V  L  K  F  S  I  P  I  K  S  I  D  C  D  G  F  D  V   220
AATGTAGTTTTAAAATTTTCAATTCCAATTAAATCGATTGATTGTGATGGTTTTGATGTA

N  V  N  Y  K  E  N  S  S  K  K  E  A  K  I  T  S  K  S  Q   240
AATGTAAATTATAAAGAGAATTCAAGTAAAAAAGAAGCAAAAATCACATCAAAATCACAG

H  T  S  G  V  K  K  N  I  I  L  I  I  Q  P  V  E  L  N  E   260
```

```
CATACAAGTGGTGTTAAAAAGAATATAATTTTAATTATTCAACCTGTTGAGCTAAATGAA

P  K  S  M  I  E  Y  I  G  G  G  D  D  K  S  Y  A  T  A  I    280  Hybrid-1a
CCAAAATCCATGATTGAATATATTGGTGGTGGCGATGATAAATCTTATGCAACAGCAATT

N  F  Y  P  S  F  K  N  V  N  P  D  E  V  Y  Q  K  S  E  F    300  start VWFA-OE
AATTTTTATCCATCATTCAAGAATGTTAATCCAGATGAAGTTTATCAAAAGAGTGAATTT
                3’-GTTCTTACAATTAGGTCTACTTCAAATAGTTTTCTCACctaggatca  VWA1_vitOE_R
                                             5'-aggggatccGAATTT     VWA1_vwaOE_F

I  F  L  I  D  C  S  G  S  M  S  G  Q  S  I  N  K  A  R  R    320  vWFA MIDAS
ATTTTCTTAATTGATTGTTCTGGTTCAATGAGTGGTCAATCGATTAATAAAGCTAGACGT
ATTTTCTTAATTGATTGTTCTGGTTCAATGAGTGG

A  M  E  I  I  I  R  S  L  N  E  Q  H  K  V  N  I  Y  C  F    340
GCTATGGAAATTATTATTAGATCATTGAATGAACAACATAAAGTAAATATCTATTGTTTT

G  S  S  F  N  K  V  F  D  K  S  R  V  Y  N  D  E  T  L  E    360
GGTTCATCTTTTAATAAGGTATTCGATAAATCAAGAGTTTACAATGATGAAACCTTAGAG

I  A  G  S  F  V  E  K  I  S  A  N  L  G  G  T  E  L  L  P    380
ATTGCAGGTTCATTTGTTGAGAAAATTTCAGCAAATTTGGGTGGCACAGAATTATTACCA

P  M  V  D  I  L  S  S  P  N  D  P  E  Y  P  R  Q  V  F  I    400
CCAATGGTTGATATTCTTTCCTCTCCAAATGATCCAGAGTATCCAAGACAAGTTTTCATT

L  T  D  G  E  I  S  E  R  D  K  L  I  D  Y  V  A  K  E  A    420
CTAACCGACGGTGAAATCTCTGAAAGGGATAAATTAATTGATTATGTAGCAAAGGAAGCA

N  T  T  R  I  F  T  Y  G  I  G  A  S  V  D  Q  E  L  V  I    440
AATACAACTCGTATCTTCACCTATGGTATTGGTGCTAGTGTCGATCAAGAATTGGTTATT

G  L  S  K  A  C  K  G  Y  Y  E  M  I  K  E  T  T  N  M  E    460
GGTCTTTCAAAAGCATGTAAAGGTTATTATGAAATGATCAAAGAGACTACCAATATGGAG

K  Q  V  M  K  L  L  N  V  A  F  E  P  M  L  S  N  I  K  L    480  hybrid-1b
AAACAAGTAATGAAGTTATTAAATGTTGCATTTGAACCAATGCTTTCAAATATTAAATTG

D  W  S  S  C  G  L  V  D  V  I  Q  A  P  S  H  I  R  P  L    500
GATTGGTCATCATGTGGTCTTGTTGATGTCATTCAAGCACCTTCTCACATTAGACCACTT

F  N  Q  E  R  M  M  I  Y  S  M  I  P  S  N  Q  T  N  Q  D    520
TTCAATCAAGAGAGAATGATGATCTATTCAATGATACCATCAAATCAAACCAATCAAGAT
   3’-GTTCTCTCTTACTACTAGATAAGTTACTATGGTAGTTTAcctaggaag-5’            VWA1_vwaOE_R
                                   5’-gcaaggatccCAAACCAATCAAGAT      VWA1_ctermOE_F

I  I  N  A  S  I  E  T  S  K  P  L  I  I  T  L  T  G  D  G    540
ATCATCAATGCCTCTATTGAGACTTCAAAACCATTAATAATCACTTTAACTGGTGATGGT
ATCATCAATGCC

P  K  G  N  V  L  S  F  P  I  T  L  D  F  K  N  D  L  S  T    560
CCAAAAGGTAATGTTTTATCATTCCCAATCACTTTAGATTTTAAAAATGATCTTTCTACA

N  S  N  Q  I  H  T  L  A  A  F  K  H  I  Q  D  L  E  E  S    580  2xαHelices
AATTCAAATCAAATTCATACTCTAGCTGCTTTTAAACATATTCAAGATCTTGAAGAAAGT

E  R  K  E  K  K  D  N  K  D  K  I  V  E  L  G  K  K  Y  G    600
GAAAGAAAAGAAAAGAAGGATAATAAAGATAAAATCGTTGAATTGGGTAAGAAATATGGT

L  V  S  K  H  T  S  Y  I  V  T  A  D  S  D  N  V  T  E  E    620  VITpt2
TTAGTTTCAAAACATACCTCTTATATTGTTACCGCTGATTCTGATAATGTCACTGAAGAA
```

```
T  M  K  T  V  D  I  M  N  Q  S  P  P  I  R  P  G  G  R  I   640
ACAATGAAAACTGTTGACATTATGAATCAATCACCTCCTATTCGTCCTGGTGGTCGTATT

V  S  R  G  G  G  R  S  G  A  S  G  A  L  S  S  S  I  L  S   660
GTTTCTAGAGGTGGTGGTAGAAGTGGTGCTAGTGGTGCTTTATCTTCAAGTATTTTGTCA

R  K  R  S  S  S  P  S  T  A  T  K  R  S  S  S  S  S  F  S   680 potential NLS
AGAAAACGTTCATCCTCCCCATCTACTGCTACTAAACGATCTTCTTCATCTTCATTTTCA

S  S  Y  L  S  L  S  S  S  S  Q  K  K  K  K  E  V  S  R  S   700
TCTTCATATTTATCTTTATCATCTTCATCCCAAAAGAAGAAAAAAGAAGTTAGTAGAAGT

D  D  D  D  D  D  E  K  I  E  N  C  V  E  S  Y  E  S  D  G   720
GATGATGATGACGATGATGAAAAAAATAGAAAATTGTGTTGAAAGTTATGAAAGTGACGGA
      5'-tgcaagtgaggatgctgaaactctcGTGTTGAAAGTTATGAAAGTGACG         5_VWF1_c_t 2

G  D  Q  S  S  E  Q  D  E  E  E  E  D  D  C  D  D  F  H  E   740
GGTGATCAATCAAGTGAACAAGATGAAGAAGAAGAAGACGATTGTGATGATTTCCATGAA

D  L  D  E  D  L  G  A  T  A  M  D  V  D  K  K  E  C  E  K   760
GATCTAGACGAAGATTTAGGTGCTACCGCAATGGATGTTGATAAAAAAGAATGTGAAAAA

E  C  K  K  K  D  S  S  K  V  D  L  K  V  K  P  S  K  V  P   780
GAATGTAAAAAGAAAGATTCTTCAAAAGTTGACCTTAAAGTCAAACCTTCAAAAGTACCT

L  P  S  R  S  P  S  V  S  K  P  T  T  T  S  L  L  S  P  S   800
CTACCATCAAGATCACCTTCAGTTTCAAAACCAACAACAACATCACTACTTTCTCCATCT

P  K  S  A  P  S  A  P  S  Q  Q  K  S  V  K  S  T  G  D  L   820
CCAAAATCAGCACCATCAGCACCATCACAACAAAAATCAGTTAAAAGTACTGGTGATTTA

L  I  D  L  L  K  I  Q  K  S  N  G  S  W  T  K  S  S  I  D   840 CTD
TTAATTGATTTATTAAAAATTCAAAAATCAAATGGTTCTTGGACAAAATCTTCTATTGAT

Q  L  K  I  P  T  D  K  A  P  A  E  L  S  T  T  E  L  N  D   860
CAATTGAAAATCCCAACTGACAAAGCACCTGCCGAATTATCAACTACTGAATTAAATGAC

I  W  V  T  I  I  V  I  A  K  I  V  K  F  F  S  S  E  K  A   861
ATTTGGGTAACCATTATCGTTATTGCAAAGATTGTAAAATTCTTCTCATCTGAAAAAGCT

Q  Y  E  L  A  I  Q  K  S  T  R  W  V  K  L  Q  L  S  K  L   881
CAATATGAACTTGCAATTCAAAAATCAACAAGATGGGTAAAACTTCAATTATCGAAATTA

N  L  P  E  N  T  F  D  K  F  L  S  N  A  K  S  L  V  *     901
AATCTTCCTGAAAATACTTTTGATAAATTTTTATCAAACGCTAAATCATTAGTTTAA
                               3'-GTTTGCGATTTAGTAATCAAATTcctaggat VWA1_OE_BamHI
                  3'-CTATTTAAAAATAGTTTGCGATTTAGTAATCAACCGTCGCCGTCG 3_VWF1_c_t 2

AAAAAAAAATTAATAAAAAAAAAAAAAATTTTGTTAAAAAAAAAAAAAAAAAAAAAAAAA
AAAAAAAAAATATAAAAAGATTAAATAAAAAATTAATATGTAACAAGACTCTATTCTTTT
ATTAATGAGAAAGATTGAATTTTGAAAAACATGTGTCATTTTTATTTTATTAATAATTAA
ATTAATGTTTTTTTTTTTTTTTATTTTTTAAATTTTTATTTTTAAACTTTAATTCTAATA
TAAAATCTTTTTTTTTTTTTTTTTTTTTATTATTATTATTGTAATTTTATTTGTTTTCTT
TTTAATATTTATTATAAATAATAATTCAGTTTATGAAAAAGAAAAGGTAGAAACAGATTT
   5'-ttaattcctgcagAATAATTCAGTTTATGAAAAAGAAAAGG                  5_VWF non_code_b

AGATAATAGAATTCCTTCACTTGATGTTTTTAATATGGATGGTTCAAGATATGTTGATAT
TTCTAAAATGAGTTCCAATGAAGAGAAATTAGCTTTTATTAGAAATGTAAGTCGTTGTCG
GTGTTGTTGTTCACTTGAAAATATTTTTAATTTTAATCATAATTTTTAAAATAATTGGTA
ATAATTAAATCTCTCTATAATTTTTAATAATTATTTTTATATATTTAAAATATTGAACTT
```

```
TTAACCAATGGTTATAGTGGATTCGTAATTGAAAAGTGAACTCTAAATCATTTGTAAATG
ATTATAGTTATAATAAAAATGAATTCTTATTAAATGGATTGGTTATCATTAGTGAATAAA
GATTTAATGGAACAGTTGTAATGGAAATGAATAGAATAATTTTAAATTGTTTCAATACAT
ATGGTTATATTGATTCTTCAGGTAGTTCAGATGTTATTAATGAGAATACAGTTTAAACGA
GGTTCTTTTAAAATTTTACCACCAATTTTTACCTAATTAATACCATAACAACCCGAGCAT
TTATAGAATTCTTTTTTTTTACTTTATTATTTATAATTTACTTCATATATTAATAATACA
CGTGTTGGAAGATGGTTCTTTTGAAATTATACCACTTCCA
              3'-GAAAACTTTAATATGGTGAAGGaataacaacaacaacaacaaaattt   5_VWF n_c_v
5'-aaattttgttgttgttgttgttattccttcaccatattaaagttttc
```

B. Vwa1 overexpresssion

```
ATGTCTGATCATCATCATCATCATCATAGATCTGATTATAAGGATGATGATGATAAGGAT BclI BglII
M  S  D  H  H  H  H  H  H  R  S  D  Y  K  D  D  D  D  K  D
            HIS6tag
TATAAGGATGATGATGATAAGGATTATAAGGATGATGATGATAAGGAATTCGGTGGTGGT EcoR1
Y  K  D  D  D  D  K  D  Y  K  D  D  D  D  K  E  F  G  G  G
                FLAG3
TTAAATGATATTTTTGAAGCACAAAAAATTGAATGGCATATCCATGGATTTAGTAGTATA NcoI Vwa1-start
L  N  D  I  F  E  A  Q  K  I  E  W  H  I  H  G  F  S  S  I
      BirA recogn site
......AAATCATTAGTTTAA                                        Vwa1-end
......K  S  L  V  *
```

C. Vwa1 VIT-domain overexpression

```
ATGTCTGATCATCATCATCATCATCATAGATCTGATTATAAGGATGATGATGATAAGGAT BclI BglII
M  S  D  H  H  H  H  H  H  R  S  D  Y  K  D  D  D  D  K  D
            HIS6tag
TATAAGGATGATGATGATAAGGATTATAAGGATGATGATGATAAGGAATTCGGTGGTGGT EcoR1
Y  K  D  D  D  D  K  D  Y  K  D  D  D  D  K  E  F  G  G  G
                FLAG3
TTAAATGATATTTTTGAAGCACAAAAAATTGAATGGCATATCCATGGATTTAGTAGTATA NcoI
L  N  D  I  F  E  A  Q  K  I  E  W  H  I  H  G  F  S  S  I
      BirA recogn site                            Vwa1-start
...... GTTTATCAAAAGAGTGGATCCTAA
...... V  Y  Q  K  S  G  S  *
       VIT/Hyb-1a end
```

D. Vwa1 vWFA-domain overexpression

```
ATGTCTGATCATCATCATCATCATCATAGATCTGATTATAAGGATGATGATGATAAGGAT BclI BglII
M  S  D  H  H  H  H  H  H  R  S  D  Y  K  D  D  D  D  K  D
            HIS6tag
TATAAGGATGATGATGATAAGGATTATAAGGATGATGATGATAAGGAATTCGGTGGTGGT EcoR1
Y  K  D  D  D  D  K  D  Y  K  D  D  D  D  K  E  F  G  G  G
                FLAG3
TTAAATGATATTTTTGAAGCACAAAAAATTGAATGGCATATCCATGGTTCCGAATTTATT NcoI
L  N  D  I  F  E  A  Q  K  I  E  W  H  I  H  G  S  E  F  I
      BirA recogn site                            vWFA-start
TTCTTA...... ATGATACCATCAAATGGATCCTAA
F  L  ...... M  I  P  S  N  G  S  *
             vWFA/Hyb-1b end
```

E. Vwa1 C-terminal region overexpression

```
ATGTCTGATCATCATCATCATCATCATAGATCTGATTATAAGGATGATGATGATAAGGAT BclI BglII
M  S  D  H  H  H  H  H  H  R  S  D  Y  K  D  D  D  D  K  D
```

```
        HIS6tag
TATAAGGATGATGATGATAAGGATTATAAGGATGATGATGATAAGGAATTCGGTGGTGGT EcoR1
Y  K  D  D  D  D  K  D  Y  K  D  D  D  D  K  E  F  G  G  G
                  FLAG3
TTAAATGATATTTTTGAAGCACAAAAAATTGAATGGCATATCCATGGTTCCCAAACCAAT NcoI
L  N  D  I  F  E  A  Q  K  I  E  W  H  I  H  G  S  Q  T  N
     BirA recogn site                                Hyb-1b start
CAAGAT......AAATCATTAGTTTAA
Q  D  ......K  S  L  V  *
            C-term end
```

F. Vwa1-N1 open reading frame

```
ATGTTTAGTAGTATAACAAGTAAATTATCAGCAATTTCAGGTGGATCAAGTAAAAATGAT
M  F  S  S  I  T  S  K  L  S  A  I  S  G  G  S  S  K  N  D   1
TATAAAGAAGCAAATTATTATAATTATTTTAGAATTTTAGAAAAGAAACAAACTGAAGAG
Y  K  E  A  N  Y  Y  N  Y  F  R  I  L  E  K  K  Q  T  E  E   21
ATTGCAAGAAAAAGATGTGGTTTATATAGTTTAAAAAATCATAATAATGTATTTGTTTTA
I  A  R  K  R  C  G  L  Y  S  L  K  N  H  N  N  V  F  V  L   41 VIT
AAAGAGTTTTCAATTGAAACTGAAATTAATGATTGTAGTTCAACATCAATTTGGACACAA
K  E  F  S  I  E  T  E  I  N  D  C  S  S  T  S  I  W  T  Q   61
AGCTATTCAAATGATTCAAATACACCAGTCGAGGCAAAGTATCAATTACCATTACATCCA
S  Y  S  N  D  S  N  T  P  V  E  A  K  Y  Q  L  P  L  H  P   81
ACATCGGTTGTATCAAATTTTCAAATTGAATATCAAGGTAAAGTAATTCAAGGTAAAATC
T  S  V  V  S  N  F  Q  I  E  Y  Q  G  K  V  I  Q  G  K  I   101
AAAGAGAAAGAGAAAGCATTAGAGAAATACAATGATGCAATTGCAAGTGGTGGTCAAGCA
K  E  K  E  K  A  L  E  K  Y  N  D  A  I  A  S  G  G  Q  A   121
TTTATGGCAACAAAATCAGATGACGGTTATTTCAATTTAACACTTGGTAATTTACCACCA
F  M  A  T  K  S  D  D  G  Y  F  N  L  T  L  G  N  L  P  P   141
AAAGAGAATGTTAAAGTTAGAGTTGTAATCTCATCAGAGTTGGGTACACATACCGATGGT
K  E  N  V  K  V  R  V  V  I  S  S  E  L  G  T  H  T  D  G   161
CAATTACATTATTGTCTTCATCGTTATATGTTCCCATCTTATGCAGGCTCTGATCATCAT
Q  L  H  Y  C  L  H  R  Y  M  F  P  S  Y  A  G  S  D  H  H   181
CATCATCATCATAGATCTGATTATAAGGATGATGATGATAAGGATTATAAGGATGATGAT     BglII
H  H  H  H  R  S  D  Y  K  D  D  D  D  K  D  Y  K  D  D  D   201
HIS6tag                                 FLAG3
GATAAGGATTATAAGGATGATGATGATAAGGAATTCGGTGGTGGTTTAAATGATATTTTT     EcoR1
D  K  D  Y  K  D  D  D  D  K  E  F  G  G  G  L  N  D  I  F   221
GAAGCACAAAAAATTGAATGGCATATCCATGGTGAAAACTTGTATTTCCAAGGTGGATCC     NcoI BamH1
E  A  Q  K  I  E  W  H  I  H  G  E  N  L  Y  F  Q  G  G  S   241
   BirA recogn site                 TEV protease site
TAA
*
```

Figure S2. Vwa2 nucleotide and amino acid sequences.

The sequence information for DdVwa2 is derived from dictyBase DDB_G0292740 (Chromosome 6 coordinates 2078010 to 2080742) and Uniprot Q54CQ8, and is consistent with all DNA and amino acid sequence data obtained in this study. See **Figure S1** legend for formatting notes.

```
AAACTCTTTATATTTTTAAAAAAACATAATATATTATTTATTTATTTATTTTTTTATTTT
                                    M  I  K  N  L  I  S   VIT
TTTATAAATATATTATATATATATATATATAAATTTAAAATGATAAAAAATTTAATTTCA
                             ‘5-acccatggaATAAAAAATTTAATTTCA VWA2_OE_NcoI

A  F  S  Q  G  V  G  I  Q  K  K  E  L  P  S  T  I  I  L  N
GCATTTTCACAAGGTGTTGGAATTCAAAAAAAGGAATTACCATCAACAATAATTTTAAAT
GCATTTTCACAAGG-3’

K  D  D  Y  K  E  A  N  Y  Y  N  Y  Y  R  N  L  E  L  N  K
AAAGATGATTATAAAGAAGCCAATTATTATAATTATTATAGAAATTTAGAATTAAATAAA

P  D  E  V  L  A  R  R  I  A  G  L  A  P  I  A  Y  Q  Y  Y
CCAGATGAAGTATTAGCAAGAAGAATTGCAGGTTTAGCACCAATTGCATATCAATATTAT

N  V  S  S  F  D  N  N  Q  F  K  I  V  D  F  S  I  D  S  K
AATGTTTCAAGTTTTGATAATAATCAATTTAAAATTGTAGATTTTTCAATTGATTCAAAA

L  N  D  T  C  L  T  C  V  W  T  Q  T  Y  K  N  E  S  K  T
TTGAATGATACATGTTTAACATGTGTTTGGACACAAACCTATAAGAATGAATCAAAAACA

P  V  E  A  V  Y  R  I  P  L  S  P  L  S  T  V  S  A  F  S
CCGGTTGAAGCAGTTTATAGAATTCCATTATCACCATTATCAACGGTATCAGCATTTTCA

V  Q  F  N  G  K  T  L  H  G  K  I  K  D  S  T  K  A  Q  E
GTACAATTCAATGGTAAAACATTACATGGTAAAATAAAAGATTCAACCAAGGCACAAGAG

K  Y  D  D  A  I  A  S  G  G  Q  A  F  L  A  E  K  S  K  D
AAATACGATGATGCAATTGCAAGTGGTGGTCAAGCATTCCTTGCAGAGAAATCAAAAGAT

D  D  N  Y  F  N  F  K  L  G  N  I  P  P  T  E  S  S  I  T
GATGATAACTATTTCAATTTTAAATTAGGTAATATACCACCAACTGAATCATCAATTACA

I  H  I  T  M  I  S  E  I  G  S  H  L  N  S  L  H  Y  L  L
ATTCATATCACTATGATCTCTGAAATTGGTAGTCATTTAAATTCATTACATTATCTATTA

H  R  Y  C  F  P  Q  S  S  N  Y  N  F  S  L  S  L  S  V  N
CATCGTTACTGTTTTCCACAATCGTCAAATTATAATTTTTCATTATCATTATCAGTGAAT

L  S  N  S  I  K  S  I  F  F  D  G  D  K  S  H  S  L  Q  Y
TTAAGTAATTCAATTAAATCAATTTTTTTTGATGGTGATAAATCACATTCACTTCAATAT

E  N  K  E  K  T  K  C  I  I  Q  Y  K  K  S  L  G  F  N  T
GAAAATAAAGAGAAAACAAAATGTATAATTCAATATAAAAAATCACTTGGTTTTAATACT

Q  P  N  I  L  I  V  F  E  L  D  D  L  N  K  P  Q  S  F  I
CAACCAAATATTCTAATTGTATTTGAATTGGATGATTTAAATAAACCACAATCATTTATT

E  K  L  S  I  N  K  E  D  I  K  N  N  P  H  S  D  S  D  S
GAAAAATTATCAATTAATAAAGAAGATATTAAAAACAATCCACATTCAGATTCAGATTCA

D  S  D  D  E  E  N  K  K  E  N  E  K  S  S  Y  A  I  A  L   Hyb-1a
GATTCAGATGATGAAGAAAATAAAAAAGAAAATGAAAAATCATCATATGCAATTGCATTA

N  F  F  P  K  F  E  S  I  N  K  E  D  I  Y  Q  K  G  E  F
AATTTTTTCCCAAAATTTGAATCAATTAATAAAGAAGATATTTATCAAAAAGGTGAATTT
```

I F L I **D** C **S** G **S** M S G N P I D S A R R vWFA **MIDAS**

ATATTTTTAATTGATTGTTCTGGTAGTATGAGTGGTAATCCAATTGATTCAGCAAGAAGA

A L E I I I R S L N E Q C K F N I Y C F
GCATTGGAAATTATTATTAGAAGTTTAAATGAACAATGTAAATTTAATATTTATTGTTTT

G S G F N K A F Q E G S R K Y D D D S L
GGTTCAGGTTTTAATAAAGCTTTTCAAGAGGGATCTAGAAAGTATGATGATGATTCTTTG

A V V N R Y V S N I S A N L G G T E L L
GCCGTGGTTAATAGATATGTTTCAAATATTAGTGCAAATTTAGGTGGAACTGAATTATTA

Q P I K D I L S K E I D P E Y P R Q I F
CAACCAATTAAAGATATCTTATCAAAAGAGATTGATCCAGAATATCCAAGACAAATTTTC

I L T **D** G A V S D R S K L I E F V S K E
ATTCTTACTGATGGTGCTGTCTCTGATAGATCAAAATTAATTGAATTTGTTTCAAAAGAA

S K T T R I F T Y G I G S S V D V E L V
TCAAAAACAACTCGTATCTTTACCTATGGTATTGGTAGTAGTGTAGATGTTGAATTGGTA

V G L S K A C K G Y Y T L I R N S S D M
GTTGGTTTAAGTAAAGCTTGTAAAGGTTATTATACACTAATTAGAAATAGTTCAGATATG

E T E V M K L L S I A F E P T L S N V S Hyb-1b
GAAACTGAAGTAATGAAATTATTATCTATCGCTTTTGAACCAACACTTTCAAATGTATCT

F D W S Q L L D L S N G K S T T I I Q S
TTTGATTGGAGTCAATTATTAGATCTAAGCAATGGTAAAAGTACAACTATAATTCAATCA

P T Q I R P I F N N E R M M V Y A T I E
CCTACTCAAATTAGACCAATTTTTAATAATGAAAGAATGATGGTTTATGCTACTATTGAA
5’-gaggatgctgaaactctcGAA VWF2 5_c_t

L D N D I S N N I E N H G Q P V I V T M
CTCGATAATGATATTTCTAATAACATTGAAAATCATGGTCAACCAGTAATTGTAACAATG
CTCGATAATGATATTTCTAATAAC-3’

N A D G P L G D R L S Y H V E L D F K N 2x-αhelices
AATGCTGATGGTCCATTAGGTGATAGATTATCTTATCATGTCGAATTGGATTTTAAAAAT

Y S Q S N S I H T L A A F K R I Q D L E
TATTCACAATCTAATAGTATTCATACATTGGCTGCTTTTAAAAGAATTCAAGATTTAGAA

E I E R K S S K E T E K L E I I K L G K
GAAATTGAAAGAAAATCTTCAAAAGAAACTGAAAAACTTGAAATCATCAAATTGGGTAAG

K Y N L V S K H T S L V V T S D S D S P VIT pt2
AAATATAATTTGGTTTCAAAACATACATCTTTAGTTGTAACTTCAGATTCTGATTCACCA

T E D T M K V I N I L P N N S Q H P I I
ACTGAAGATACAATGAAAGTTATAAATATTCTTCCAAATAATTCTCAACATCCAATTATT

V D R C H T F A V N F N S P L Q Y Q Q Q
GTTGACAGATGTCATACTTTTGCTGTTAATTTTAATTCACCACTTCAGTACCAACAACAA

Q Q Q Q Q Q N F N S G F A P P P P P M M
CAACAACAACAACAACAAAATTTCAATAGTGGATTTGCACCACCACCACCACCAATGATG

S S G P P P P P G S S F G A P P P P P P
TCATCAGGACCACCACCACCCCCAGGTTCATCTTTTGGTGCACCTCCACCTCCACCCCCA

```
G  G  A  F  P  T  S  S  I  S  E  K  K  S  S  S  Q  S  S  S
GGTGGAGCTTTCCCAACATCATCAATATCAGAAAAAAAATCATCATCACAATCATCATCA

S  Y  L  P  P  T  M  S  L  S  R  K  S  S  L  S  P  S  S  P
TCATATTTACCACCAACAATGTCATTGTCAAGAAAATCATCATTGTCACCATCATCACCA

S  K  N  Y  P  S  P  K  L  S  S  P  S  L  S  Y  G  S  T  Q
TCAAAAAATTATCCAAGCCCTAAACTCTCAAGTCCTTCTTTATCGTATGGTTCAACTCAA

S  E  S  T  P  S  N  D  P  L  I  S  L  L  A  K  Q  K  A  N   CTD
TCAGAAAGCACTCCATCAAATGATCCATTAATTTCTTTATTAGCTAAACAAAAAGCAAAT

G  S  W  S  K  S  S  I  Q  D  Q  F  S  S  A  I  S  K  I  P
GGTTCTTGGAGTAAATCATCTATTCAAGATCAATTCTCTTCTGCTATTAGTAAAATACCA

N  E  L  S  A  V  E  D  V  W  A  T  L  L  V  I  S  K  I  M
AATGAACTTTCTGCTGTTGAAGACGTTTGGGCAACTTTATTAGTTATCTCAAAAATTATG

K  T  F  A  S  Q  K  S  K  W  E  L  S  V  Q  K  S  N  K  W
AAAACATTTGCTTCTCAAAAATCAAAATGGGAACTTAGTGTTCAAAAATCAAATAAATGG

V  K  Q  Q  L  L  K  L  N  L  S  F  D  Q  F  L  E  L  A  K
GTTAAACAACAATTATTAAAATTAAATCTTTCTTTTGATCAATTTTTAGAACTTGCAAAA
                                 3'-CTAGTTAAAAATCTTGAACGTTTT  VWA2_OE_BamHI
                                           3'-ATCTTGAACGTTTT  VWF2 3_c_t

S  N  V  *
TCAAATGTATAAAATAAAATTTAAAAAATTAATTAAAATAATAAAATAATAAATATATTT
AGTTTACATtctagactaata-5'
AGTTTACATATTcctaggta-5'

AAAACAATTCAATTGATTGTAACATAAAATATCTTATTTTATTTAAAATTTTTAAAATTT
ATTGATGAGTCAATATTTAAAAAATAATTTTTAAGATTTGCATTTAGATATTTTTTAATT
TTTAATATCTTGATTTTTTTTTTTTTCTTTTTTTTATTTCTCGAAGCCCCATTTTATTTT

TAATATATATTTTTTTTATTTAGAATATGATTTGAGAATATGGTTTGAGAATAAGAAATA
                        5'-gttaattcctgcagGGTTTGAGAATAAGAAATA 5_V2 non_code_b

TTATTTATCTTTTTCTAATTATTATTAAAATTTTTTTTATATTTTTTTTTTTTTTAAAAA
TTATTTATC-3'

AAAAAAAAAAAAAATATAAAAAGAAAATTTTTTTTCTATTTTTAATTATAAAATAAATA
TTAATAAAAATTTAAAAACATAAAATTTATAAAATATTTTAAATAAAAAAAAAAATCAAT
AAAAGAAAAATGTCAATTTTAATTACTGGTAGTACTGGCAAATTTGGAAAAAATTCAATC
GAATTTTTATTATCAAAAGGTGTTGACCCAAAATCAATAATTGCATTGGTTCGTGAAGAA
TCAAAAGGTAAACAATTCCAAGAAAAAGGTTTAAATGTTAGAATTGGTGATTATACAAAT
GAAGAATCTTTAGTAAAGGCTTTCCAAGGTATTGAAAAATTATTATTCATTTCATCAAGT
GATATTGAAAATAGAATTGATCATCATAATAATGTAATTAAAGCAGCTAAACAAGTTGGT
ACAATTAAACACTTGGTTTATACAAGTTTCTTTAGAAAAGAAGAGGTTGAAAAAAGTGGT
AATTCTTCAATTGGTTTTGTTGGTAAACCACATATTATTGCTGAAGATTTGATTGAAAAA
TCTGGAATAAATTATACAATTCTTCAAAATATTCTTTATACCGATTTCTTAACAGAATTC

TTTTTTGGTGAAAAGGTATTAGAGAATGGTATTTTTTTCCCAGCAGGTAATGGTATTGGT
                                     3'-GTCGTCCATTACCATAACCa 3_V2 n_c_v

AACTATGCCATTAGAAAAGATATGGCTGAAGCTGCAGTAAATATATTATTGGATGAAAAT
ataacaacaacaacaacaaaat

CAACAAAAACATCTAAATAAAAAGTATATCATTTCTAATGAAGAAAGTTCATCATTTGAA
```

Fig. S3. Vwa1-like proteins in *D. discoideum*.

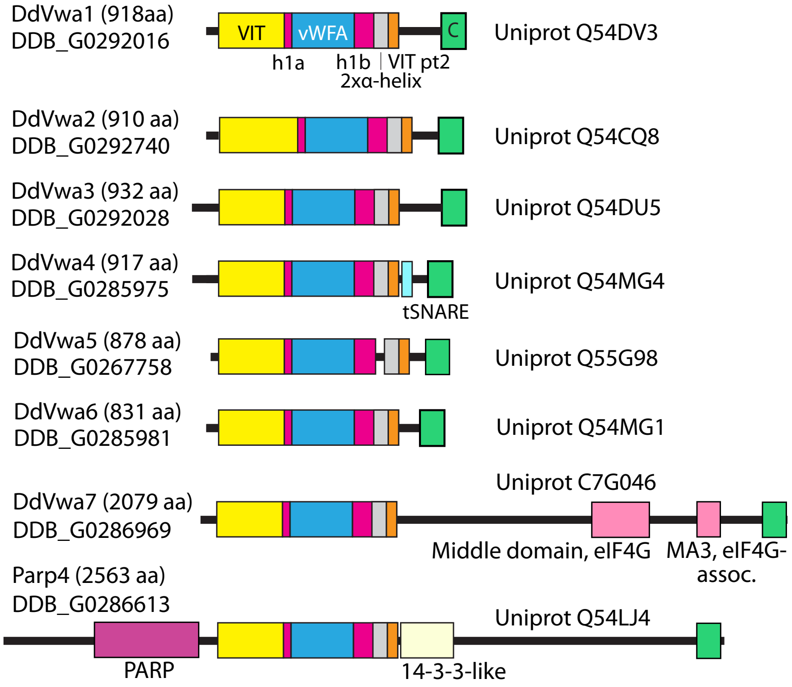

Figure S4. Amino acid alignment of HsITIH1, DdVwa1 and HsVWA5 proteins. Amino acids are color-coded based on chemical properties, and positions are highlighted according to conservation of a DdVwa1 residue with either HsITIH1 or HsVWA5 at the corresponding position, as previously described (West et al., 2002). Predicted conserved domains are differentially underlined or italicized according to the key at the right. Amino acids predicted to coordinate Mg^{+2} in the MIDAS domain are bold italicized. Percent identity and similarity values among the 3 sequences are summarized at the end.
HsITIH1 is from UniProtKB P19827, ITIH1, Inter-alpha-trypsin inhibitor heavy chain H1, isoform 1, length: 911 a.a.
DdVwa1 is from dictyBase DDB_G0292016 (chromosome: 6 position 1027245-1030001) and Uniprot Q54DV3, length: 901 a.a.
HsVWA5A is from NP_001123614.1, von Willebrand factor A domain-containing protein 5A isoform 1 [Homo sapiens], length: 786 a.a.

```
HsITIH1         MDGAMGPRGLLLCMYLVSLLILQAMPALGSATGRSKSSEKRQAVDTAVDGVFI 53 SigPep VIT
DdVWA1   MFSSITSKLSAISGGSSKNDYKEANYYNYFRILEKKQTEEIARKRCGLYSLKNHNNVFVL
HsVWA5A                                                     MVHFCGLLTLHREPVPL

HsITIH1  RSLKVNCKVTSRFAHYVVTSQVVNTANEAREVAFDLEIPKTAFISDFAVTADGNAFIGDI 113
DdVWA1   KEFSIETEINDCSSTSIWTQSYSNDSNTPVEAKYQLPLHPTSVVSNFQIEYQGKVIQGKI
HsVWA5A  KSISVSVNIYEFVAGVSATLNYENEEKVPLEAFFVFPMDEDSAVYSFEALVDGKKIVAEL

HsITIH1  KDKVTAWKQYRKAAISGENAGLVRAS--GRTMEQFTIHLTVNPQSKVTFQLTYEEVLKRNHM 173
DdVWA1   KEKEKALEKYNDAIASGGQAFMATKS--DDGYFNLTLGNLPPKENVKVRVVISSELGTHTDG
HsVWA5A  QDKMKARTNYEKAISQGHQAFLLEGDSSSRDVFSCNVGNLQPGSKAAVTLKYVQELPLEADG

HsITIH1  QYEIVI-KVKPKQLVHHFEIDVDIFEPQGISKLDAQASFLPKELAAQTIKKSFSGKKGHV 232
DdVWA1   QLHYCLHRYMFPSYAFNFNYNVVLKFSIPIKSIDCDGFDVNVNYKENSSKKEAKITSKSQ
HsVWA5A  ALRFVLPAVLNPRY[i]LSMVATIDSQHGIEKVQSNC-PLSPTEYLGEDKTSAQVSLAAG
                        i=QFSGSSKDSCLNVKTPIVPVEDLPYT

HsITIH1  LFRPTVSQQQSCPTCSTSLLNGHFKVTYDVSRDKICDLLVANNHFAHFFA--PQNLTNM  289 HC-Hyb1a
DdVWA1   HTSGVKKNIILIIQP-VELNEPKSMIEYIGGG-DDKSYATAINFYPSFKNVNPDEVYQ-
HsVWA5A  HKFDRDVELLIYYN---EVHTPSVVLEMGM[i]LMGDPSAMVSFYPNIPEDQPSNT---
                                        i=PNMKPGH

                                     H      H H
                                     *  **  *****
HsITIH1  -----NKNVVFVIDISGSMRGQKVKQTKEALLKILGDMQPGDYFDLVLFGTRVQSWKGSL 344 vWFA MIDAS
DdVWA1   -----KSEFIFLIDCSGSMSGQSINKARRAMEIIIRSLNEQHKVNIYCFGSSFNKVFDKS
HsVWA5A  -----CGEFIFLMDRSGSM[i]RIQAAKETLILLLKSLPIGCYFNIYGFGSSYEACFPES
                             i=QSPMSSQDTSQL

             H          H
           ***  *       *
HsITIH1  VQASEANLQAAQDFVRGFSLD-EATNLNGGLLRGIEILNQVQESLPELSNHASILIMLTD 403 MIDAS
DdVWA1   RVYNDETLEIAGSFVEKISANLGGTELLPPMVDIL--------SSPNDPEYPRQVFILTD
HsVWA5A  VKYTQQTMEEALGRVKLMQADLGGTEILAPLQNIY--------RGPSIPGHPLQLFVFTD

HsITIH1  GDPTEGVTDRSQILKNVRNAIRGRFPLYNLGFGHNVDFNFLEVMSMENNGRAQRIYEDHD 463
DdVWA1   GEISERDKLIDYVAKEANT-----TRIFTYGIGASVDQELVIGLSKACKGYYEMIKETTN
HsVWA5A  GEVTDTFSVIKEVRINRQK-----HRCFSFGIGEGTSTSLIKGIARASGGTSEFITGKDR

          **  *  **  *
HsITIH1  ATQQLQGFYSQVAKPLLVDVDLQY--PQDAVLALTQNHHKQYYEGSEIVVAGRIADNKQS 521 HC-Hyb1b
DdVWA1   MEKQVMKLLNVAFEPMLSNIKLDWSSCGLVDVIQAPSHIRPLFNQERMMIYSMI-PSNQT
HsVWA5A  MQSKALRTLKRSLQPVVEDVSLSWHLPPGLSAKMLSPEQTVIFRGQRLISYAQL------

HsITIH1  SFKADVQ----------AHGEGQEFSITCLVDEEEMKKLLRERGHMLENHVERLWAYLTI 571
DdVWA1   NQDIINASIETSKPLIITLTGDGPKGNVLSFPITLDFKNDLSTNSNQ-------IHTLAA
HsVWA5A  --TGRMPAAETTGEVCLKYTLQG-----KTFEDKVTFPLQPKPDVNL------TIHRLAA

HsITIH1  QELLAKRMKVDREERANLSSQALQMSLDYGFVTPLTSMSIRGMADQDGLKPTIDKPS--E 629 3x-α-helix
DdVWA1   FKHIQDLEESERKEKKDNKDKIVELGKKYGLVSKHTSYIVTADSDNVTEET-MKTVDIMN
HsVWA5A  KSLLQTKDMGLRETPASDKKDALNLSLESGVISSFTAFIAINKELNKPVQGPLAHRDVPR

HsITIH1  DSPPLEMLGPRRTFVLSALQPSPTHSSSNTQRLPDRVTGVDTDPHFIIHVPQKEDTLCFN 689 VITpt2 rHC1 end
DdVWA1   QSPPIR--PGGRIVSRGGGRSGASGALSSSILSRKRSSSPSTATKRSSSSSFSSSYLSLS
HsVWA5A  PILLGA--SAPLKIKCQSGFRKALHSDRPPSASQPRG

HsITIH1  INEEPGVILSLVQDPNTGFSVNGQLIGNKARSPGQHDGTYFGRLGIANPATDFQLEVTPQ 749
```

```
HsITIH1  NITLNPGFGGPVFSWRDQAVLRQDGVVVTINKKRNLVVSVDDGGTFEVVLHRVWKGSSVH 809
DdVWA1   SSSQKKKKEVSRSDDDDDDEKIENCVESYESDGGDQSSEQDEEEEDDCDDFHEDLDEDLG

HsITIH1  QDFLGFYVLDSHRMSARTHGLLGQFFHPIGFEVSDIHPGS                     849
DdVWA1   ATAMDVDKKECEKECKKKDSSKVDLKVKPSKVPLPSRSPS

                                           H      H H H
                                           * **  ** ****
HsITIH1  DPTKPDATMVVRNRRLTVTRGLQKDYSKDPWHGAEVSCWFIHNNGAGLIDGAYTDYIVPDIF* 911
DdVWA1   V-SKPTTTSLLSPSPK--SAPSAPSQQKSVKSTGDLLIDLLKIQKSNGSWTKSSIDQLKIPT  C-term bundle
HsVWA5A  -ELMCYKAKTFQMDDYSLCGLISHKDQHSPGFGENHLVQLIYHQNANGSWDLNEDLAKILGM

                                                    HH  H
                                             ** *** **  *
DdVWA1   ---DKAPAELSTTELNDIWVTIIVIAKIVKFFSSEKAQYELAIQKSTRWVKLQLSKLNLPEN--TFDKFLSNAKSLV* 901
HsVWA5A  SLEEIMAAQPAELVDSSGWATILAVIWLHSNGKDLKCEWELLERKAVAWMRAHAG-STMPSVVKAAITFLKSSVDPAIFAF* 786
```

Identity and Similarity Values for region corresponding to residues 44-650 of DdVwa1, up to the end of VITpt2 region, after trimming of non-conserved inserts

HsITIH1 vs DdVwa1:
Alignment length: 529
Percent identity: 15.7
Percent similarity: 34.6

HsITIH1 vs HsVWA5:
Alignment length: 531
Percent identity: 14.7
Percent similarity: 31.6

DdVwa1 vs HsVWA5:
Alignment length: 509
Percent identity: 25.7
Percent similarity: 43.2

Identity and Similarity Values for region corresponding to residues 44-650 and 818-901 of DdVwa1, to also include the C-terminal region

Results for DdVwa1 vs HsVWA5:
Alignment length: 615
Percent identity: 24.4
Percent similarity: 42.3

Similarity groups: GAVLI, FYW, CM, ST, KRH, DENQ, P

Figure S5. Alignment of representative Vwa1-like proteins from throughout phylogeny.
(A) Regions corresponding to the VIT, Hyb-1a, vWFA, and Hyb-1b domains of HsITIH1-HC (heavy chain) (Fig. 7), corresponding to amino acid residues 60-538 of DdVwa1, were aligned in Geneious and manually refined. Representatives were taken from across eukaryotic phylogeny and several bacteria, and all *D. discoideum* and human homologs are included. Sequences positions are color coded and highlighted as in **Figure S4**. Predicted conserved domains are labeled at the top. I=ITIH-like; P=PARP-like; V=HsVwa5A-like; VB=HsVwa5B-like. For analysis of evolutionary relationships in **Figure 6**, poorly conserved sequences present in only some proteins were deleted. **(B)** Gene identifiers.

```
                                 .                            VIT                           .

Tepidicaulis marinus I        86 MVATDVDIDVAG-PIARAKVTQRFENPSDG----------WVEG----------------  118
Halopseudomonas pelagia       31 LNSWLNHSETGT-LMVRHDAESDWHP-ALLLGT-------EVDIQISGPVARITLQQHFS   81
Dunaliella salina             38 LKSRDVQAHVYA-DARFSAVEERLNFIPDK----------DAQV----------------   70
Papaver somniferum I             ------------------------------------------------------------
Coffea arabica I              89 GVSLEVDCFLDT-AFVTVSGSWRVHCVMGSR---------ACDC----------------  122
Populus alba                  94 AVAMEIDCCFDH-ANVCFSGAWRVHCIKASR---------KCDV----------------  127
Physcomitrium patens I        95 KSKLNVECCIST-GFVTSKCTWNLNCVRSQA---------SCDC----------------  128
Arabidopsis thaliana          85 GIEMNIDCWLDT--FVTVTGRWRVHCVRPSK---------RFDC----------------  118
Chenopodium quinoa            91 GVEVEVDC-YLD-MAFVHL-SATWRLHCVDAAQ-------KCDC----------------  124
Tetrahymena thermophila       22 LVFSNYQCKIIN-NILQVELTQRFQDQNNFDSNLK-----IYDSNEA-------------   62
Physcomitrium patens V        23 LQHVSVTGHIRD-AVAQYTLEQTFLNPEKEA---------GIDA----------------   56
Chlamydomonas reinhardtii     28 EKPIPVPLKRRD-VSATVYAAASFSDTKETLSY-------VSDVECSA------------   67
Trichomonas vaginalis         16 YTSISIEGIQKD-TFVSFNIKQVFTNTTCE----------NTDL----------------   48
Paramecium tetraurelia        46 LQGVHQFLKVQQ-GFFIVELQQTYSTFGYQE---------PIEL----------------   79
Homo sapiens VB              127 LFVANLGTIAPM-ENVTIFISTSSELPTLP----------SGAV----------------  159
Mus musculus VB               15 LMESDVTSYVSG-YALGLTASLTYGNLEAQ----------PFQG----------------   47
Danio rerio VB                12 ALPLSISDITSC-VRG-YTLALTASLTYENIEDH------PIEG----------------   47
Heterostelium album P        687 LKAVHVRAKLLD-LIGEVTLFQHYNNATSK----------AIEA----------------  719
Dictyostelium discoideum P   776 LKSVHVKGKILD-LIGEVTIYQHYQNNSKN----------MIEA----------------  808
Acanthamoeba castellanii P   692 LKAVHIKASLLD-MIGRVVVMQHYRNDSEH----------AIEA----------------  724
Trichoplax adhaerens         274 LTDVHFRVKLLD-LASEVVVLQRYYNNSNN----------SIEA----------------  306
Crassostrea virginica P      632 LDEAHIRARLVD-LAGEVVVFQKYSNNSDQ----------ALEA----------------  664
Branchiostoma floridae P     550 LKSVHVRAKLLD-LAAQVVVLQSYKNENNV----------AIEA----------------  582
Entamoeba invadens            20 FDKVHFNLQVIG-VSAKVTVSHYLTNNSNA----------VISL----------------   52
Entamoeba dispar              16 LTSINFEIETIG-NNAKVKAIHSILNESNN----------DCNV----------------   48
Heterostelium album V         66 LTDYQVDAVVSD-ATVTTVMTQQYKNDSTT----------PVEA----------------   98
Mus musculus V                17 LKSIAVTLSIND-FVAGVSATLNYENEEKS----------PLEA----------------   49
Homo sapiens V                33 LKSISVSVNIYE-FVAGVSATLNYENEEKV----------PLEA----------------   65
Branchiostoma floridae V      15 LTEIDVNVDIIG-YVSNVTSSLQYHNEEDN----------PIEA----------------   47
Crassostrea virginica V       19 VKKIDIEVSIQG-FIANVRSDLHYINDSDE----------NLET----------------   51
Danio rerio V                 17 LKSISVQLQVRG-HVSSVRATLQYVNEEER----------PLEA----------------   49
Cavenderia fasciculata        70 LKDFHIKTEMTD-VSSITVFTQRYFNGYPS----------PVEA----------------  102
Tieghemostelium lacteum       38 LNSFSIVSDIQN-STLKSKYVQVYENNSSN----------PVEA----------------   70
D. discoideum Vwa2            79 IVDFSIDSKLND-TCLTCVWTQTYKNESKT----------PVEA----------------  111
D. discoideum Vwa1            60 LKEFSIETEIND-CSSTSIWTQSYSNDSNT----------PVEA----------------   92
Polysphondylium violaceum     76 LKEFNIESEMTD-SCISSVFTQSYTNSLTS----------PVEA----------------  108
Dictyostelium purpureum V     66 LENFDITSELND-TSIVSVWTQEYSNDSKS----------PVEA----------------   98
Acanthamoeba castellanii V    39 IKAVSVEAKVTD-FCSSVTVRQAFVNAEST----------PIEA----------------   71
Mus musculus P4              616 LDSVHIKGRVID-FVAQVIVFQTYTNQSHV----------PIEA----------------  648
Homo sapiens P4              623 LEDVHIKGRIID-TVAQVIVFQTYTNKSHV----------PIEA----------------  655
Danio rerio P4               694 LQAVNVKCKLMD-LMGEVIIFQTYTNQSAV----------PIEA----------------  726
Amphimedon queenslandica      22 LRSLAVEGDING-YVVGLSIKLIYDNDNTG----------PVEV----------------   54
Mus musculus I1               50 IKSLKVNCKVTS-RFAHYVITSQVVNNADK----------AREV----------------   82
Danio rerio I4                31 IYSFYINSTVSS-RYATTIITSRVANKLSE----------PQEI----------------   63
Crassostrea virginica I       28 IYFLHVTSEVDY-RFAKTVVTSKVVNKEPT----------ASEA----------------   60
Homo sapiens ITIH1            53 IRSLKVNCKVTS-RFAHYVVTSQVVNTANE----------AREV----------------   85
Folsomia candida              26 LKSVSITASVVH-AVAQVGIT-QFYENNENT---------PIEA----------------   58
Daphnia magna                 46 LTGVSVEVQIID-LGAKVTIEQRYVNRENQ----------PIEA----------------   78
Neurospora crassa             32 QLELSVSTHIVG-SSSRTTLTQTFVNPSAEKS--------IPEL----------------   65
Tuber magnatum                18 LISTTLRSHVVD-LTASHTLTQVYDNNDSSSRD-------IAEA----------------   53
Rhizophagus irregularis       18 LQNVVVEANVVD-MIAEVTISQTYKNVEKD----------TIEA----------------   50
Penicillium solitum           22 QVSLEAHATILS-SAARTTLTQTFVNPSDRA---------IEEI----------------   55
Vitrella brassicaformis       43 LVSVHAEANVLD-TVAETVLCQTYMNDRDK----------PIEA----------------   75


                                 .                            VIT                           .

Tepidicaulis marinus I       119 ---------VYVFPLPEDSAVDSLRMLIGD-RIIEGEIKKKEEARQIYEE------AKEA  162
Halopseudomonas pelagia       82 NSLNMTAEGVYAFPLSERSAVHGMQMQVG-QRRIVGAIHEKAEARQIYQQ------ALSS  134
Dunaliella salina             71 ---------TFKFPLPHRAVVHRFEATMGT-RELRTEVKPAAAAEAEYHE------AVAQ  114
Papaver somniferum I             ------------------------------------------------------------
Coffea arabica I             123 ---------RIVIPMGEQGSILGVEVEVP--KKSYSTQLISMDDERNAEKG---------  162
```

```
Populus alba                  128 ---------RIAVPMGEQGSLLGVEVGVPG--RSYHSQLIQAEDANDKEKK---------  167
Physcomitrium patens I        129 ---------LLAVPIDNQGTVSSVEIDMG-HDRLYTTIVVPTDEAASYCTK--GNTQAD-  175
Arabidopsis thaliana          119 ---------CVGVPMGEKGSFLGAEIDVLNNEKSYQTKLVTEDETSDFDN----------  159
Chenopodium quinoa            125 ---------LLAIPMGEQGSVLGFEIDASG-----GSYLSQLVKVEDATD-------LDG  163
Tetrahymena thermophila        63 ---------IYYLPVDQNICIESFQATING-KTAKGVVKKINEAKDLYLQGRQ-EGNIVG  111
Physcomitrium patens V         57 ---------VYTFPLYEGAAVVGFEAEVNG-RKIVGKVQEKAAAKKEYEE------AKSA  100
Chlamydomonas reinhardtii      68 ---------VFRFPLPPRAAVYRFRAVFGD-REVVTKVKPRAAAREEFDL------AVSQ  111
Trichomonas vaginalis          49 ---------TYYFPNESQFCTYETLFNIDG-KEIKPSLREKDEAKQVFNE------AREA   92
Paramecium tetraurelia         80 ---------EYLFSINQNAAVTKMVVELGD-KKVYGIIKEKEEAKQEYEE----GVKQGK  125
Homo sapiens VB               160 ---------RVLLPAVCAPTVPQFCTKSTG----TSNQQAQGKDRHCF-------GAWAP  199
Mus musculus VB                48 ---------LFVYPIDEYSTVVGFEAVIAD-RVVTIQLRDKAKLDRSHLDIQ---PATVT   94
Danio rerio VB                 48 ---------IFIYPLDECSVVVGFEAMIGS-QIITVQVKDKTKIDDCYFDC----CDANG   93
Heterostelium album P         720 ---------KYVFPLDEMGAVCGFEAFING-KHVVGEVKEKERAHREYKQ------AISE  763
Dictyostelium discoideum P    809 ---------KYVFPLDEMGAVCGFEAYVNG-KHIIGECKEKEKAQREYRE------AVAA  852
Acanthamoeba castellanii P    725 ---------KYVSPLDELGAVCGFEAFING-KHIIGEVKEKEKAHKEYQQ------AVSE  768
Trichoplax adhaerens          307 ---------KYVFPLDERAAVCGFEAFINN-KHIVGQVKEKERAHKEYKE------AVSR  350
Crassostrea virginica P       665 ---------KYVFPLDDMAAVCGFEAFING-KHIVGEVKEKETAHREYKE------AIRE  708
Branchiostoma floridae P      583 ---------KYVFPLDDMAAVVGFEAFING-KHVVGEVKEKEEAHREYRQ------AISE  626
Entamoeba invadens             53 ---------LVHFSYDSTCSITGYSFKTKS-KEMVSCLKEKESAKRAIAD------ARAS   96
Entamoeba dispar               49 ---------SIIMTLDDESAVTGYSIKTVN-GEIESELKEKEQAKIEQSD------ATTN   92
Heterostelium album V          99 ---------KYEMPLPPYAAVNKFVVTYDG-KVLFAKIKERQAAAEKYDD------AIAS  142
Mus musculus V                 50 ---------FFVFPMDEDSAVYSFEAFVDG-KKIVAELQDKYQAHKRYEE------ALSG   93
Homo sapiens V                 66 ---------FFVFPMDEDSAVYSFEALVDG-KKIVAELQDKMKARTNYEK------AISQ  109
Branchiostoma floridae V       48 ---------VFVFPLDETSAVYKFEALIEG-RRVIGKVKEKQKAREEYDD------AIAS   91
Crassostrea virginica V        52 ---------KFLFPLDSDSAVYKFEAEIDG-RTIVAEVQEKSQARRIYHD------AIND   95
Danio rerio V                  50 ---------LFVFPLPAEAALCSFSATVGG-QEIRGELRDKQTARDEYDDG------VSA   93
Cavenderia fasciculata        103 ---------KYQIPLAPYSAVSDFVVQYDD-KVLRGTVKGKQDAANDFSD------AIAS  146
Tieghemostelium lacteum        71 ---------FYELPLPLTSSICDLEVTYQD-QTFTAKIKEKQKAQEKYDD------AIAS  114
Dictyostelium discoideum V2   112 ---------VYRIPLSPLSTVSAFSVQFNG-KTLHGKIKDSTKAQEKYDD------AIAS  155
Dictyostelium discoideum V1    93 ---------KYQLPLHPTSVVSNFQIEYQG-KVIQGKIKEKEKALEKYND------AIAS  136
Polysphondylium violaceum     109 ---------KYVLPLPPYASVSNFVVEYDG-KVLKGKIKEKEKAANKYSD------AIAS  152
Dictyostelium purpureum V      99 ---------NYKIPLSPSSVVSRFVVEYKD-KILEAKIKEKSKAKEKYAD------AIAS  142
Acanthamoeba castellanii V     72 ---------IYQFELEEKATVSEFVALIDG-NKIKGTIQEKEDAKNTYDD------AIAS  115
Mus musculus P4               649 ---------KYIFPLDDKAAVCGFEAFING-KHIVGEIKEKEEARQEYRE------AVSQ  692
Homo sapiens P4               656 ---------KYIFPLDDKAAVCGFEAFING-KHIVGEIKEKEEAQQEYLE------AVTQ  699
Danio rerio P4                727 ---------KFVFPLEETAAVCGFEAFING-KHVIGKVKEKEQARKEYKQ------AIEK  770
Amphimedon queenslandica       54 ---------TFRMPLEEKYVLTDLTALIDG-RRIKADVREKEEAKVVYDD------AIAS   98
Mus musculus I1                83 ---------AFDVEIPKTAFISDFAITSDG-KAFIGDIKDKVTAWKQYRK------AAVL  126
Danio rerio I4                 64 ---------QFEVKIPKNAFISKFRMIIEG-KTYDGVVKKKEEAQQQYNK------AVSR  107
Crassostrea virginica I        61 ---------VFDLTLPNEAFITAFKLTING-KEIEGKVKEKEQAKQEFEA------AKAK  104
Homo sapiens I1                86 ---------AFDLEIPKTAFISDFAVTADG-NAFIGDIKDKVTAWKQYRK------AAIS  129
Folsomia candida               59 ---------SYIFPVDSNGAVTYFQAELDG-KVIKGQVKSSEDARKDYDE------EIMD  102
Daphnia magna                  79 ---------VYLFPLDEGAGVCQFTAEVDG-RIIQGVAKETEEAKTDYEH------AIQS  122
Neurospora crassa              66 ---------RYTFPLYDGVSVVGFVCTINEDRVIRGVVKERYEARKQYQE------AIDR  110
Tuber magnatum                 54 ---------RYTFPLYESSAVTDFHALVGE-RTIKGVVREKAEAKAIYQA------AKSA   97
Rhizophagus irregularis        51 ---------FYKFPIYEAAAICGFEAEIDGQRKIKGIVKESKEAAKEYTE------AVQK   95
Penicillium solitum            56 ---------SYHFPLYDGVSVVGFECKVGS-RLLHSKAKTKSQANADYKN-----AVAQH  100
Vitrella brassicaformis        76 ---------KYCFPLVESSAVTGFWAESEG-KEIMGIVKEKVQAKKEYDE------AISA  119


                                  .                         VIT                            .
                                  ____________________________________________________________

Tepidicaulis marinus I        163 GK-----------RASLIEQQRP--NIFTNSVANIGP-GETIVVQIEYQQTVRQDENIFS  208
Halopseudomonas pelagia       135 GQ-----------RAGLVEQRRP--NLFSISVANIPA-AESISVTLEYTELLVPDGNRF-  179
Dunaliella salina             115 GH-----------SAAKVKQDPES-SIYTVDLGNLTA-HEPCIVRLHYLCLLECFAGTLE  161
Papaver somniferum I            1 -------------------------MDTGKLVKSHRG-GFLTPEMFAITIPQVEGGSNIS   34
Coffea arabica I              163 ------------AKAEDVCFLKP--HIFTLTIPQVDG-GSNLSVKVKWSQKLSYHDGQFT  207
Populus alba                  168 -----------VSRGWNGRLIKG--SMYSFEIPEVGG-GSTFSIKLTWSQKLLYHEGQFS  213
Physcomitrium patens I        176 --------------VNGPGTYNP--ELFRLKIPQVEG-GAKLEVKVTWFQSMIFDNGMYS  218
Arabidopsis thaliana          160 -----------VHKDKDSRFFKS--HIYTFKIPHVAG-GSIFSVNVTWSQKLIYKDGKFH  205
Chenopodium quinoa            164 SD-----------KLEYGGFVKP--NIFTLKIPKVEG-GSKILVKAKWSQRLIYENGWFL  209
Tetrahymena thermophila       112 YC-----------EKKIFQREVG--ECLEIRLGNIPQ-KFCIDIKLKYSQQLQVILNTFY  157
Physcomitrium patens V        101 GK-----------LASLLEQESP--DVFQTSIGNIPA-STAIIIRITLWSEMKHDAEENQ  146
Chlamydomonas reinhardtii     112 GH-----------SAVHMSQHEAGASVFEVSLGNLEA-HTEVTVEFSYLRLLDAFGGTL-  158
Trichomonas vaginalis          93 GR-----------HAMFGEDTGN--GLNSFKLGNLPK-DKTVEIHLKVSFLADIDENGYF  138
Paramecium tetraurelia        126 TM------------AYSEQDEEFP-EIKRVKIGALAP-QKELKITFEYIQPLEVFLNKF-  170
Homo sapiens VB               200 GS------------WNKLCLATL--LNTEVSNPMEY--EFNFQLEIRGPCLLAGVESP--  241
Mus musculus VB                95 GNFPEEESPIAPGKVTLDEDLER--VLFVVNLGTIAP-MENVTVFISTSSELPTLPSGA-  150
Danio rerio VB                 94 TPQSV------GGHVVLDEDQER--TVLVVSLGVIPP-LETISILVSTSSELGTLPNGG-  143
Heterostelium album P         764 GH-----------GAYLMDEEKP--DVFTVSVGNIPP-YSEVLIKITYITELSLDGLDI-  808
Dictyostelium discoideum P    853 GH-----------GAYLMDEDKP--DVFTVSVGNLPP-NCDVLIKIVYVTELSIDGLDI-  897
Acanthamoeba castellanii P    769 GH-----------GAYLMDEEKP--EVFTVSVGNLPP-KCDALIKITYVTELEVDGDNI-  813
Trichoplax adhaerens          351 GD-----------GAYLMDEDSP--DVFTVSVGNLPA-KANVVIKITYVTELAIEDEYL-  395
Crassostrea virginica P       709 GH-----------GAYLMDQDEETPEVFTVSVGNLPP-QAVVVIKITYVTELQVEGELI-  755
Branchiostoma floridae P      627 GH-----------GAYLMDEETP--DVFTVSVGNLPP-RASVLIKITYVAELAVEGENI-  671
Entamoeba invadens             97 GY----------NTSSLVQTDE---TSFDLSLDILNP-QETVELKIEYITQLSVVADEL-  141
Entamoeba dispar               93 GY-----------SSSIMEQVDD--TTFSILLGLVNK-HSEVTFSIEYLTHMEIQEEEL-  137
Heterostelium album V         143 GN-----------QAFLVEKNSS--GTFSTVIGNIPP-GKEVTVSVTTVSEIGTHLEDL-  187
Mus musculus V                 94 GY-----------QAYLLEEDKCSRDVFCCNVGNLQP-GSKVSLTLRYVQELPLEDDGAL  141
```

```
Homo sapiens V               110 GH-----------QAFLLEGDSSSRDVFSCNVGNLQP-GSKAAVTLKYVQELPLEADGAL  175
Branchiostoma floridae V      92 GE-----------GAFLFEEDDRSGDVFKCSVGNLPP-KTSATIQLSYVAELPVEADSSL  139
Crassostrea virginica V       96 GH-----------TAMYLSESETAGDLFYMELGNLPA-RTAAKLSFSYVQELDLSRDKIG  143
Danio rerio V                 94 GR-----------QVFLLEESDQSADVFRLSVGCLPA-GGAATVSFTYSTELTVEEDGGL  141
Cavenderia fasciculata       147 GG-----------QAFLGEKNDK--GYFNLSIGNLPP-GKEVTIAITIVSEVGLHLQDL-  191
Tieghemostelium lacteum      115 GG-----------QGFMITRDDN--GIFKLSLGNIEP-KTPITVTFTIIGEVYSHLESI-  159
Dictyostelium discoideum V2  156 GG-----------QAFLAEKSKDDDNYFNFKLGNIPPTESSITIHITMISEIGSHLNSL-  203
Dictyostelium discoideum V1  137 GG-----------QAFMATKSDD--GYFNLTLGNLPP-KENVKVRVVISSELGTHTDGQL  182
Polysphondylium violaceum    153 GG-----------QAFLGEKTSD--GYFSLQIGNIPP-AKDVVVRITIISEIGAHLDSL-  197
Dictyostelium purpureum V    143 GG-----------QAFMGEKTDD--GLFNFMIGNLPP-GEKVTIRLTIVSEIGTHLNSL-  187
Acanthamoeba castellanii V   116 GH-----------GAYLMEEKEDEPNIFTVNVGNLPP-GKSVDIAITYVTELEFDEGQQL  163
Mus musculus P4              693 GH-----------GAYLMDQDTP--DVFTVSVGNLPP-RAKVLIKITYITELSIQSPVA-  737
Homo sapiens P4              700 GH-----------GAYLMSQDAP--DVFTVSVGNLPP-KAKVLIKITYITELSILGTVG-  744
Danio rerio P4               771 GH-----------GAYLMDQDAP--DVFTISVGNLPP-GASVLIKVTFITELVVRSGSI-  815
Amphimedon queenslandica      99 GL-----------PAALGEELSK--DIFSVSLGNLPG-GSKAEIELSLDWQLPIDAEGKL  144
Mus musculus I1              127 GE-----------SAGLVRASGR--NMEQFTIHITVGAQSKATFRLTYEEVLKRRLMQ--  171
Danio rerio I4               108 GE-----------SAGLIKSVGR--TLEDFKTSVTVAANSKVTFELTYEELLKRRLG---  151
Crassostrea virginica I      105 GQ-----------SAGHIVAKPR--ETNKFQIQVNVAAQEKVTFVLTYRELLKRTNGL--  149
Homo sapiens I1              130 GE-----------NAGLVRASGR--TMEQFTIHLTVNPQSKVTFQLTYEEVLKRNHMQ--  174
Folsomia candida             103 ND-----------TVFLGEETKP--DVF-------PKTRASAKVVIEYVTEVKNDPDCHA  142
Daphnia magna                123 GH-----------SAFLVEEKLP--DVFKAKIGNLAA-GSGAKIRLTYVTELKVEGGEI-  167
Neurospora crassa            111 GETA---------GLLEQLPNAS--DVFTTTVGNVPA-GASLKVEVTYLGELKNDAGVDG  158
Tuber magnatum                98 GK-----------VTALFSQDTS--EVFTTSLGNIPA-RSLVVVVIKYVHELKQDLEVDG  143
Rhizophagus irregularis       96 GH-----------GAYLLESESE--DVFQCSVGNITS-GQTVIIKITYVTELKHDSETEK  141
Penicillium solitum          101 QT-----------AAVMDHTSMN--DVFVIRLGNVPA-HGKINVDITFVGELKQDSQTDG  146
Vitrella brassicaformis      120 GH-----------GAYLMEQERP--DIFQASVGNILP-GKTVKVFVKYCHELSIFDKRI-  164


                                 .                            VIT                             .
Tepidicaulis marinus I       209 LRFP-----TVVAPRYNPAPSVHLVDFQPGENGWGSVSDPVP---------DRGKITPPV  254
Halopseudomonas pelagia      180 ---------SLRLPLTMTP-RFNPLAEPSDAEGP--------------------------  203
Dunaliella salina            162 WVHTSTWVPPYILPELDEG-SAEKAAKAVPT-----------------------------  191
Papaver somniferum I          35 VKINWSQKLSFTSGQFSLTIPFKFPEFVTPPVKGNSKIEKI-------------------   75
Coffea arabica I             208 L------NIPFSFPDYVTPAGKKISKKEKIH-----------------------------  232
Populus alba                 214 L------NVPFSFPSFVNPIGKKISKREKIL-----------------------------  238
Physcomitrium patens I       219 L------RVPFVFPEHILP-IATKLSSIIKV-----------------------------  242
Arabidopsis thaliana         206 L------NVPFRFPSYVNPIGKEIIKREKIVL----------------------------  231
Chenopodium quinoa           210 L------KLPFSFPVYVTP-GGKISKKQKIL-----------------------------  233
Tetrahymena thermophila      158 ---------SVDLSLVLKE-SHEQINQNSFILDL---------------------LCSQK  186
Physcomitrium patens V       147 V--------RFVLPTVIAP-RYGWPAPEVNLE----------------------------  179
Chlamydomonas reinhardtii    159 ---------EWSHTATWVP-PYVGSAGDVATGVD-----------------------KVA  185
Trichomonas vaginalis        139 F--------KFPLSTKYQK-GIMTNEYSDK------------------------------  159
Paramecium tetraurelia       171 ----------WKVEVYPMI-DENYFNLQQQQQVRLQSVYFQS---------LNRYIQKYV  210
Homo sapiens VB              242 ---------THEIRADAAPSARSAKSIIIT------------------------------  262
Mus musculus VB              151 --------VRVLLPAICAPTVPPSCTHRFGSSSPQPQGKDPH-CFGTQTKDSYNRLCLAT  201
Danio rerio VB               144 --------IRVVSPSVCSPRVQRSVKEEQAFSPSTARRRDQH-HCSHEQTA--SGVCLAA  192
Heterostelium album P        809 ---------SFILPSSISP-AQQAVSGAQVTQ---------------------------SS  832
Dictyostelium discoideum P   898 ---------SFVLPRSITP-KQRLQSSSSNTQS-------------------------VTS  923
Acanthamoeba castellanii P   814 ---------VFRLPASVSA-QQQKSAAAQTTQ----------------------------T  836
Trichoplax adhaerens         396 ---------DFFLPSNLAP-WKTGAALSNKTQD--------------------------TV  420
Crassostrea virginica P      756 ---------NFRLPGSVAP-WKQKYVDTPEFKD----------------------------  778
Branchiostoma floridae P     672 ---------CFRLPGSVAP-WQKDSLSEKIQKD----------------------------  694
Entamoeba invadens           142 ---------LFTLPKNSER-SKSTI------------------------------------  156
Entamoeba dispar             138 ---------IIKIPNLIES-KEK--------------------------------------  150
Heterostelium album V        188 ---------HFCLHRFMFP-DKKFD-----------------------------------  202
Mus musculus V               142 ---------RYVLPAILNP-RYHLSDGREDSN--------------------------LDM  166
Homo sapiens V               176 ---------RFVLPAVLNP-RYQFSGSSKDSC--------------------------LNV  182
Branchiostoma floridae V     140 ---------KFVLPGVLNP-RYSSDTGTAAYDD------------------------TYMA  166
Crassostrea virginica V      144 ---------TFMLPTVINP-RYMPEYTPTDEQHVDEDDENLS---------SQAQQTPMI  184
Danio rerio V                142 ---------RYCLPAVLNP-RYTPAAAGAGVPQ----------------------------  164
Cavenderia fasciculata       192 ---------HYCLHRYMFP-KNSLS-----------------------------------  206
Tieghemostelium lacteum      160 ---------HYFIHSASFP-TSYKFK----------------------------------  175
Dictyostelium discoideum V2  204 ---------HYLLHRYCFP-QSSNYN----------------------------------  219
Dictyostelium discoideum V1  183 ---------HYCLHRYMFP-SYAFN-----------------------------------  197
Polysphondylium violaceum    198 ---------HYCLHRYMFP-KTNFS-----------------------------------  212
Dictyostelium purpureum V    188 ---------HYCLHRFMFP-LNNFK-----------------------------------  202
Acanthamoeba castellanii V   164 ---------RFRLPSNNDNPSYKAAAASS-------------------------------  183
Mus musculus P4              738 ---------IFFIPGTVAP-WQQDKALNENLQD-------------------------TV  762
Homo sapiens P4              745 ---------VFFMPATVAP-WQQDKALNENLQD-------------------------TV  769
Danio rerio P4               816 ---------VFSLSGSVAP-WQESAALNQTTQ---------------------------T  838
Amphimedon queenslandica     145 ---------SFTLPAVLKP-RYTPATSQGIGAT----------------------PAPPS  172
Mus musculus I1              172 ----------YDITIKVRP-KQLVQH----------------------------------  186
Danio rerio I4               152 ---------KYELLINAQP-MQPVAD----------------------------------  167
Crassostrea virginica I      150 ----------YNHVIYINP-GQVVDD----------------------------------  164
Homo sapiens I1              175 ----------YEIVIKVKP-KQLVHH----------------------------------  189
Folsomia candida             143 I--------RFFIPTTVAP-RYVPPTDNDAKS---------------------------RD  167
Daphnia magna                168 ---------RFYLPTTIAP-RYVPSTDGSSAA---------------------------KD  191
Neurospora crassa            159 I--------RYTIPTSVAP-RYGDFPGTLLDA-----------------------------  181
Tuber magnatum               144 V--------KLLVPTIIAP-KYGIAPVTTDLVFGNRWRTCW-------------GAFSRE  181
```

```
Rhizophagus irregularis      142 I--------RFVLPTNIAP-RYGSSEYSSSSNDG-K-------------------ILNPD  172
Penicillium solitum          147 I--------RYTLPSTIAP-RYGTSTPYDSTQL--------------------------SL  172
Vitrella brassicaformis      165 ---------RFTLPTHIAP-RYTPEHQPASEVPQNVTYV-----------------TPGT  197


                                 .                            VIT                         .
                                 -----------------------------------------------------------
Tepidicaulis marinus I       255 ---AHPDTGKRNPLTLTLSLDAGFPLGDITSPHH---------------EIALVREDES  295
Halopseudomonas pelagia      204 -QSQQQDPSPEGRILANEHLPAQSHQVMISTYL-----------------DAGQQLQSL  244
Dunaliella salina            192 -----FAKEVSYTLSYVVHIYSSTGVRSVSSAEAI--------------NEAEGLPGSS  231
Papaver somniferum I          76 ------------TLNVESGAEAEVLGLKVSHPLK----------------IVRKQVGRL  106
Coffea arabica I             233 -------------LNVNCGPETEVLCKTTSHPLK----------------ELRRQAGQL  262
Populus alba                 239 -------------LSVNSGVGKEILCRCASHALK----------------ELRREVGKM  268
Physcomitrium patens I       243 ------------KCSINTGTNGGVELGAFGNPM---------------EEILREH-GKV  273
Arabidopsis thaliana         232 --------------NMNSCVSGGEIASSFTSHPLK---------------IIHRVAGEL  261
Chenopodium quinoa           234 -------------LNVNSGTNAEVQCKAATHPLK----------------ELKRQVGKF  263
Tetrahymena thermophila      187 ---ITFFKNFGISLLVQQVDENHTAFYTRQLDRSQTQICQEGGQIVQFKLIYQFENMHV  242
Physcomitrium patens V       180 ------LPPPFKLLSVNMSCAMSKAITSIKSPSHTIEVHLGN-----SSGETPTAESFE  217
Chlamydomonas reinhardtii    186 AALPTFAPKVTYVLSYEVTVRAEAGTVRAIESPEPVTVERPA-----AAAEAAAGAGAE  239
Trichomonas vaginalis        160 ----------PDSFHFSLKVNTQKELSDFKVSV--------------DGTKNVIDSH--  192
Paramecium tetraurelia       211 ---YLEKFEYNFKQNITASIDIGSPITYYKSPTHSI----LSGNAKIESAKQSMEEEHP  262
Homo sapiens VB              263 ---LANKHTFDRPVEILIHPSEPHMPHVLIEKGDMT-------------LGEFDQHLKG  305
Mus musculus VB              202 LLDTKVTNPMEYEFKFQLEIRGPCLLAGVESPTHE-------------IRADAAPSAHS  247
Danio rerio VB               193 LLEEETINSIDYEFNFHLEIRGPYLLAGVESPSHA-------------IRADADPSARS  238
Heterostelium album P        833 TSTVQVKEIGKSNFTVQIGIEMPYNIVKLNSPTH-----------------QIRSKKTH  874
Dictyostelium discoideum P   924 TVQVTELAQKQSDLSISVGIEMPYNIVKLISPTHD---------------VRIKRTHTK  967
Acanthamoeba castellanii P   837 TTETVSVEATNARFGLQIGIDMPYEILRIASPSHG-------------HLLRLKQTATK  882
Trichoplax adhaerens         421 G-VVKIDEAKAIDASIQVSVEMPFEIRAIMSPTH-----------------NIKQKCTA  461
Crassostrea virginica P      779 -KDWDTVQVTEGKCSVAICVDMPFDIRSLECPTH-----------------KIRVKKTL  819
Branchiostoma floridae P     695 LETVKVEKHAGKEFSLQVAMEMPFDIRTIQSPSH-----------------KIRVKRTA  736
Entamoeba invadens           157 ---------------FDVEYFTNNLKKTVN---------------------KIHIENPD  179
Entamoeba dispar             151 ---------------YQIPYSI-----KIIGSS------------------KFSFKGIL  171
Heterostelium album V        203 -------------FKLNLTVNLSSQIQSIAVDDWS----------------SADKQIT-  231
Mus musculus V               167 KTPIVPLEELPYTLSMVATISSQLGISTIQSNCP---------------LNPIEYTGDN  210
Homo sapiens V               183 KTPIVPVEDLPYTLSMVATIDSQHGIEKVQSNCP---------------LSPTEYLGED  226
Branchiostoma floridae V     167 PTGDVVTSDAPYKLKLKVNVSSPNSIDKIESPKSS-----------------IDVTYGG  208
Crassostrea virginica V      185 AADVETLYNSESKIHLTIHVSGGRNLKEFENK--------------------MELEILA  223
Danio rerio V                165 ---VCSASVIPYSLSLSADVRSSVPIARLDSSCA---------------LEPLQFLDPQ  205
Cavenderia fasciculata       207 -----------LKLDMEVDLSTDITDIQIDHYQ-----------------FKKEINQNR  237
Tieghemostelium lacteum      176 -------------FSLSITVTLSTGISNIESLYSD---------------YKIKVERTS  206
Dictyostelium discoideum V2  220 -------------FSLSLSVNLSNSIKSIFFDGDK---S-----------HSLQYENKE  251
Dictyostelium discoideum V1  198 -------FNYNVVLKFSIPIKS-IDCDGFDVN--------------------VNYKENS  228
Polysphondylium violaceum    213 -------------MNYKLNVALSVPIHDIEIENYS---------------PKIEYHDES  243
Dictyostelium purpureum V    203 -----------FNYNLNISLSS--PMKDIVIDSY---------------KPSIKYSDG-  232
Acanthamoeba castellanii V   184 ------NSAPNLKLRIDFDMTS--NIRSLSSPSHPI---------------TFEFGEEP  219
Mus musculus P4              763 ETIRIKEIGAEQSFSLAMSIEMPYMIEFISSDTH-----------------ELRQKSTD  804
Homo sapiens P4              770 EKICIKEIGTKQSFSLTMSIEMPYVIEFIFSDTH-----------------ELKQKRTD  811
Danio rerio P4               839 TVEKIGLSESKGEFSLSMSIEMPYEIINLNCSHR------------------IKTKMTD  879
Amphimedon queenslandica     173 EASVDGQAGVSQFLLRVHNATSIASVTSATHSI----------SVEEQADADIKVVTLS  221
Mus musculus I1              187 -----------FEIDVDIFEPQGISKLDAQASFLS-------EELAAQTIKKS--FSGK  225
Danio rerio I4               168 -----------FKIDVHIQENPGISFLEVKGDLN-------------TGDLASAVKTTR  202
Crassostrea virginica I      165 -----------LKINVFIKESSNITIIKTPQIRN---------------ELLPEGPEGT  197
Homo sapiens I1              190 -----------FEIDVDIFEPQGISKLDAQASFLP-------KELAAQTIKKS--FSGK  218
Folsomia candida             168 LANMSFSPRAPAPLDIKVIVSIQGDIKSVESLSS--------HKILVESRGPI-PDRPS  217
Daphnia magna                192 LASIKYSQESHYKVEFNITVQMASAIKEIRSPTH--------KITVSSDAANMPTRRT-  241
Neurospora crassa            182 -PQAVAKSGIQITVDVETPGGS--NIKSIQSPSH-----PISVTIGHTSSGAAAGTDMS  232
Tuber magnatum               182 IRDIGNAEDVGLSLSVTTTMSG--TIRGISSPSH-----PISIGLGSHSSDDDADFDPK  233
Rhizophagus irregularis      173 VVSYSNKADFYLDLAVTCRMTS--TIQNIESPSH---------------KISTEMNIDE  214
Penicillium solitum          173 FGLPATLKGMRITVDVQMEKGL--VIRELESPSH-----RVKVSLGRVSSTPATSSTFE  224
Vitrella brassicaformis      198 TPDMG-ANAAKLSLTAKMAMPS--KIDSIESPSH-----------------PIKVSKME  236


                                 .                             VIT                         .
                                 ------------------------------------------------------------
Tepidicaulis marinus I       296 --RAELTLSQGEVP----------------ADKDFELTWTPAKT-DAP--TAALFRE---   331
Halopseudomonas pelagia      245 --NSPSHTVKSEYD-----------------GRGYRLVLPAEQV-PMDR-DFVLDWQLQT   283
Dunaliella salina            232 --CQRVVSLASDVAD---------------PSWDFRLLVELAQP---DNPPLCTLNLQRC   271
Papaver somniferum I         107 --ECSYEADVSTWSQ--------------NKFRFSYAISCTAV---STSDILGGLLLY-   145
Coffea arabica I             263 --GFSYESEVLTWSS---------------CDFIFTYNISTSQ----MYGGALLQ-----   296
Populus alba                 269 --GFLYDAEVLTWSS---------------SDFSFSYNVRSKD----LFGGVLLQ-----   302
Physcomitrium patens I       274 --IFKKG--GNDWKN---------------QDFIASYKTWSDG----IFPNLIFQ-----   305
Arabidopsis thaliana         262 --SCEYEAEVPSWSR---------------VDFGVSFTVSSGDL---CG-NVLVK-----   295
Chenopodium quinoa           264 --GFLYEEEINTWSQ---------------TDFEFSYSVFFSD----IHGGFLLH-----   297
Tetrahymena thermophila      243 --PQVLYGACESQVHINKDDKSNKQIDVQNNSFMISFVPDFNFKFKSQINDAIVQ-----   295
Physcomitrium patens V       218 --PNRARVTLTSDSF-------------LEKDFVLVVQALGL-----NEPCALVER----   253
Chlamydomonas reinhardtii    240 --EVWRVRLSEQVAD-------------PSKDLSLAIELDPK---AARRSGLRVQRTPAS   281
Trichomonas vaginalis        193 ---NATFETNEA----------------PKKDAIFIETPIKD-----EDKSIAVS-----   223
Paramecium tetraurelia       263 --RKLILMLEDTPSNFI-------------PTKQFTLLFTSDEI---NLPRAVLSHTN--   302
Homo sapiens VB              306 --RTDFIKGMKKKSR---------------------------------------------   318
Mus musculus VB              248 A-KSIIITLAKKHT--------------FDRPVEILLHPSEP-----HMPHVLVEKGDMT   287
Danio rerio VB               239 A-TSIVVTLADKYT--------------YDCPVEILIYPSEP-----HLPHVLIEDGDMT   278
```

```
Heterostelium album P       875 --TKATVELDRVESL--------------GTNFQLLIGLEDP-----YSPRMWVEV----   909
Dictyostelium discoideum P  968 A--TIELNNQDNQYL--------------DKNFQLLIGLEEP-----YSPRMWVEV----  1002
Acanthamoeba castellanii P  883 A--TVQLK-PDASLQ--------------GKQFVLLVALEKA-----NEPRMWIEVN---   917
Trichoplax adhaerens        462 --TRAVVDTGKITSF--------------DKGFRLLIRLAEI-----HVPRMWAET----   496
Crassostrea virginica P     820 --TKAVVALDKDQMI--------------GDGFQLLIGLAEI-----HVPRMWVES----   854
Branchiostoma floridae P    737 --SKAVVELEKNCML--------------GAGFLLQVGLAEI-----HVPRMWAER----   771
Entamoeba invadens          180 L-KAFGYSLGKVQ---------------HGLYVTEDAETGE------KTGSAVFVS----   213
Entamoeba dispar            172 --SGEEEISVP-----------------LNGDLILGMKDED-------TDEVVISS----   201
Heterostelium album V       232 D-NKATVKLSSKEG--------------VKKNIIISIKPKADQ----EKPSYFIEKT---   269
Mus musculus V              211 K-TSAQVSLAEGHK--------------FDRDVELLIFYRKV-----HSPSVAVEMGM--   248
Homo sapiens V              227 K-TSAQVSLAAGHK--------------FDRDVELLIYYNEV-----HTPSVVLEMGM--   264
Branchiostoma floridae V    209 --TSAQVRLKDDHK--------------LDSDVELYVHYKDK-----HRPFAVTELGQ--   245
Crassostrea virginica V     224 --DSIGNVMTASKELN------------PGTEFSVSIHYTGF-----DKPRTVIEKG---   261
Danio rerio V               206 H-THAQVSLSAGHR-------------FDKDVELLLYYVDP------HQPSAVLEAGAA-   244
Cavenderia fasciculata      238 ----ATISLEHNGV--------------VNKNLIVVVKPKTE-----EKPGYFLEY----   270
Tieghemostelium lacteum     207 NNSSIITFKNDEFSAT------------YGRDLVFVIEPKQS-----KNMETMVEL----   245
Dictyostelium discoideum V2 252 KTKCIIQYKKSLGFN-------------TQPNILIVFELDDL-----NKPQSFIEKLSIN   293
Dictyostelium discoideum V1 229 SKKEAKITSKSQHTSG------------VKKNIILIIQPVEL-----NEPKSMIEYIG--   269
Polysphondylium violaceum   244 K-KTATITFNKSNG--------------IDKNIIAIVVPEPS-----EKPESFIEL----   279
Dictyostelium purpureum V   233 K-KKANVQFTSTG---------------VPKNVIAIIEPETS-----DKPQSLIEY----   267
Acanthamoeba castellanii V  220 N--QATVTLSSDSAQAQ-----------VAKDLIVLTKLAKP-----HQACGRVEVD---   258
Mus musculus P4             805 --CKAVVSTVEGSSL-------------DSGGFSLHIGLRDA-----YLPRMWVEK----   840
Homo sapiens P4             812 --CKAVISTMEGSSL-------------DSSGFSLHIGLSAA-----YLPRMWVEK----   847
Danio rerio P4              880 --CKAVISTVPGQTL-------------GPDGLQVSFSLSNI-----HMPRMWVEN----   915
Amphimedon queenslandica    222 E-EHALI---------------------LKTDLVIQIGLIEP-----HSPIAVVEKG---   251
Mus musculus I1             226 K-GHVLFRPTVSQQQ------SCPTCSTSLLNGEFKVTYDVNRD---KLCDLLVAN----   271
Danio rerio I4              203 A-DKDAWVTFYPTRDQQTKCTNC---AENGLNGDLIITYDVNRGN--PKGEVQISN----   252
Crassostrea virginica I     198 N--KLAVVDRISPTKAFVSYEPSPLEQESGISGQFIVQYDVDRSN--DAGDLLFMD----   249
Homo sapiens I1             219 K-GHVLFRPTVSQQQSCPTCSTS----LLNGHFKVTYDVSRD-----KICDLLVAN----   274
Folsomia candida            218 W-NKAVVTLSGNVTE-------------MDRDYVLLVTPMEPV----MKPRVYCESGHGP   259
Daphnia magna               242 --LRLDDEVTS-----------------MDRDLVVYVQIAEP-----HQPRIIQEK----   273
Neurospora crassa           233 L-QKASATLALGTAE-------------LAQDFILQVVATNT-----GNPIALLETH---   270
Tuber magnatum              234 K-AHATLALALPSLTG------------LDKDFTLLINANNI-----GDPRAFLAPH---   272
Rhizophagus irregularis     215 N-PKISKV-TLSEQITY-----------LEKDFILVVKSKDL-----NQPRAFVEYN---   253
Penicillium solitum         225 P-SQASAS-VISQQNNTSVI--------LEQDFVILIKADGL-----DTPCALLERH---   266
Vitrella brassicaformis     237 G-TSAEVTLALPE---------------VFLAADIIIQVHVVDL---HTPRVFWQLL---   274
```

```
                                                        .          Hyb-1a          .

Tepidicaulis marinus I       332 ------------------------ELNGED----------------------YVLMMLMP   345
Halopseudomonas pelagia      284 ELTS-TASVFSEI------------IDGDN----------------------HALVMLMP   308
Dunaliella salina            272 QRNT-HTKTVGLASFCLP-RTTPERSRGVE----------------------GRFKSVYT   307
Papaver somniferum I         146 -------------------SPSKQDLDKRE----------------------MFCFYLYP   164
Coffea arabica I             297 -------------------SPSLFDVDRRE----------------------AFCFYLFP   315
Populus alba                 303 -------------------SPFLRDFDDRQ----------------------MFCCYLFP   321
Physcomitrium patens I       306 -------------------DPEPGESDNRG----------------------SFCLSISP   314
Arabidopsis thaliana         296 -------------------SPSPWDSDDRG----------------------IFCLYLFP   314
Chenopodium quinoa           298 -------------------SPSSNNIDQRE----------------------MFAFYLFP   316
Tetrahymena thermophila      296 -----------------------SIAENQN----------------------IFSQEYLN   310
Physcomitrium patens V       254 ----------------------HPRDGTH-----------------------AITLTFLP   268
Chlamydomonas reinhardtii    282 RG---------------------GEQRT------------------------VALATFVP   296
Trichomonas vaginalis        224 -------------------------SDG------------------------YIAISTNP   234
Paramecium tetraurelia       303 ----------------------NDALFT----QKYCATISFIPKFNQTTLDDAYSQ-YLD   335
Homo sapiens VB              319 --------------------------------AERKTEIIRKRLHKDIPHHSVIMLNFCP   346
Mus musculus VB              288 LG---EYDQHLKGKADFIRGTKKDNSAE------RKTEVIRKRLHKDIPHHSVIMLNFCP   338
Danio rerio VB               279 AE---EYDEHLKGRSDYIKATKKDCSNE------KTVDIIRKRLHKDILHNPVVMLNFCP   329
Heterostelium album P        910 -----------------------DNSGH----------------------HASMLA-FYP   923
Dictyostelium discoideum P  1003 -----------------------DEKGH----------------------HASMLA-FYP  1016
Acanthamoeba castellanii P   918 -----------------------PQTHT-----QAAMLNNQS----PLPGAVHLKVAFYP   945
Trichoplax adhaerens         497 ----------------------HPEKGS----------------------QACMLT-FYP   511
Crassostrea virginica P      855 -----------------------NEGES----------------------QACMLT-FFP   868
Branchiostoma floridae P     772 ----------------------HPDKDS----------------------QACMLT-FYP   786
Entamoeba invadens           214 ------------------------KECDG-------------------------------   218
Entamoeba dispar             202 --------------------------------------------------------TFIN   205
Heterostelium album V        270 ----------------------ETTPAE-----------------------YAVGLNFYP   284
Mus musculus V               249 --------------------SGRPSDSLMGAPS-------------------AMVSFYP   268
Homo sapiens V               265 ---------------PNMKPGHLMGDPS-------------------------AMVSFYP   284
Branchiostoma floridae V     246 -----------------------GTDGFMADHT--------------------VMLTFVP   262
Crassostrea virginica V      262 ----------------------KNDPKS------------------AYLSSDILMVSFVP   281
Danio rerio V                245 ----------------TAPAGSLMADP-------------------------LLMLSLYP   263
Cavenderia fasciculata       271 ----------------------NKEDKT-----------------------AALALNFYP   285
Tieghemostelium lacteum      246 ------------------------DEKE---------------------QTLALSVTYYP   260
Dictyostelium discoideum V2  294 KEDIKNNPHSDSDSDSDDEENKKENEKSS----------------------YAIALNFFP   331
Dictyostelium discoideum V1  270 ----------------------GGDDKS-----------------------YATAINFYP   284
Polysphondylium violaceum    280 ----------------------DPSDKS-----------------------YAVGINFYP   294
Dictyostelium purpureum V    268 ----------------------DQKEKT-----------------------CALALNFYP   282
Acanthamoeba castellanii V   259 ---------------------EKGTSKT-------------------------VMVSLFP   272
Mus musculus P4              841 ----------------------HPEKES----------------------EACMLV-FQP   855
```

Homo sapiens P4	848	----------------------HPEKES----------------------EACMLV-FQP	862
Danio rerio P4	916	----------------------HPEKDS----------------------QACMLV-FYP	930
Amphimedon queenslandica	252	-------------------KGSDGTFKSDH----------------------LLMINFMP	270
Mus musculus I1	272	--NYFTHFFA	279
Danio rerio I4	253	--GYFVHYFA	260
Crassostrea virginica I	250	--GYFVHFLA	257
Homo sapiens I1	275	--NHFAHFFA	282
Folsomia candida	260	GSFE----------------DRRATSAL-RHSSLTIHNKSPRFYQTLESGSTAAMTHLIP	302
Daphnia magna	274	-----------------------SPEGS-----------------------AAMMLSLVP	287
Neurospora crassa	271	----------------------TDIPHQ-----------------------RALMATLVP	285
Tuber magnatum	273	-----------------------PTLTD----------------------TSTLMVTMVP	287
Rhizophagus irregularis	254	------------------------PETQT----------------------NCVMLTLVP	267
Penicillium solitum	267	----------------------PTIPNQ-----------------------RALMATLVP	281
Vitrella brassicaformis	275	PERY-FEEQREKRLALADQTEGGGKKDD-KMEVDKDKEGDKVDVKACCPYRGAAMMSIVP	332

end start vWFA .

Tepidicaulis marinus I	346	PQSKDASRKTP--------PREAIFVIDNSGSM---------AGASMPQAKASLELALQ	387
Halopseudomonas pelagia	309	GDLASDNERM---------PREVIMVVDTSGSM---------AGERMRQARESLIYALE	349
Dunaliella salina	308	PEQSSSQPEPTAEPRQAGPKKELVLVVDCSGSM---------SGDPIKFARDAALYFVR	357
Papaver somniferum I	165	GHNQSKKVFR----------KEVVFLIDISGSM---------QGRPLESVKDALFAALR	204
Coffea arabica I	316	G-NQSRKVF----------KKTLIFIIDISGSM---------RGKPLDDTKNAILSSLS	354
Populus alba	322	GDNQSMKAFR----------KEVVFLMDISGSM---------KGNPFESAKNGILSSLQ	361
Physcomitrium patens I	315	PDPNKIKVFQ----------RAVVFLLDRSGSM---------KGKPIEAARQALYFGLE	364
Arabidopsis thaliana	315	GTTKHTKLF----------KRRVVFVIDISASM---------KWKPLEDVKKALLECLA	354
Chenopodium quinoa	317	GETQNRKVF----------KKRVIFLVDISGSM---------KGAPIENVKKEVLAFLS	356
Tetrahymena thermophila	311	SFNQQIIDQTDSS------KCEFIFLLDRSGSM---------SGQSIQNAIEALILFIK	354
Physcomitrium patens V	269	RFALRPMSS-----------SELIFVVDRSGSM---------QGTPIKQAGQALELFLR	307
Chlamydomonas reinhardtii	297	PLPSPPAAGADGKQQL---RKEIWFVVDCSGSM---------DGSPINQAREAALFFVR	343
Trichomonas vaginalis	235	SFSGKIESN-----------SEFYFVVDCSGSM---------SGARIINAVKCMRLFIQ	273
Paramecium tetraurelia	336	GLNIAQNQVINR--------GNYLFFIDRSGSM---------TGARINKAKQSLLLFLK	377
Homo sapiens VB	347	DLQSVQPCLRKA-------HGEFIFLIDRSSSM---------SGISMHRVKDAMLVALK	389
Mus musculus VB	339	DLQSVQPNPRKA-------HGEFIFLIDRSNSM---------SKTNIQCIKEAMLVALK	381
Danio rerio VB	330	DLRSTTSDLRSIQ-------GEFVFLIDRSGSM---------SGVNINRVKDAMVVILK	372
Heterostelium album P	924	KLDIEHGDQP----------SIVTIVLDLSASM---------HGDPFEDMMRAVRLTIT	963
Dictyostelium discoideum P	1017	KLDIDNTMKDS--------HTMVTLLIDLSSSM---------AGDAFEDLLRAVRITIS	1058
Acanthamoeba castellanii P	946	RFEYKASRDEE---------REFILVVDCSSSM---------KGDTLEDMKRAVIASLD	986
Trichoplax adhaerens	512	EFETVETKE-----------PEIILVLDMSNSM----------KDCLLDVKKLALLLLN	549
Crassostrea virginica P	867	EFEADEESE-----------VEVVLMIDSSNSM---------KDSALQDAKKAALLTLH	907
Branchiostoma floridae P	787	EFQAEVMQG-----------HEVILLLDGSNSM---------RGSALEAAKKVALLCLC	825
Entamoeba invadens	219	-------------------PVDVIFVCDRSGSM---------DGEGITALKVALQLFLR	249
Entamoeba dispar	206	KEKEGDI--------------NIIFICDRSGSM---------YGEGINALRNMLQLFLR	241
Heterostelium album V	285	TLTVT-PEDVDQ-------HSEFIFLIDCSGSM---------SGSQIQKAKLALEILMR	326
Mus musculus V	269	DIPEVETTTKC---------GEFVFLMDRSRSMNSPMSSKDKSQLRIDAAKETLILLLK	318
Homo sapiens V	285	NIPEDQ-PSNTC--------GEFIFLMDRSGSMQSPMSSQDTSQLRIQAAKETLILLLK	334
Branchiostoma floridae V	263	DLSREDLVANC---------GEFIFILDRSGSM---------SGNKIKNARETLLLFLK	303
Crassostrea virginica V	282	EI-TVT-KDIP---------CEFIFVIDRSASM---------RGDRVEKAKETLLLLLK	320
Danio rerio V	264	EFPAAVMSSLTS-------HGEFIFLVDQSGSMDCPMHHGEGAQMRIESARDTLLLLLK	315
Cavenderia fasciculata	286	RFQIS-PDEVDQ-------KSEFVFLLDCSGSM---------SGGAITKAKRALEILMR	327
Tieghemostelium lacteum	261	QFSNIDPLDINQ-------KSEYIYILDCSGSM---------SGNLIESAKNTLNILMR	303
Dictyostelium discoideum V2	332	KFESINKEDIYQ-------KGEFIFLIDCSGSM---------SGNPIDSARRALEIIIR	374
Dictyostelium discoideum V1	285	SFKNVNPDEVYQ-------KSEFIFLIDCSGSM---------SGQSINKARRAMEIIIR	327
Polysphondylium violaceum	295	NFRIAA-DEVDQ-------KSEFIFLIDCSGSM---------SGGPIKKAKVALEIIMR	336
Dictyostelium purpureum V	282	VFDDVCVEDISQ-------KSEYIFVVDCSGSM---------SGTPITKAKRALEICVR	325
Acanthamoeba castellanii V	273	QLELADDEDIY---------TEMIFIVDRSGSM---------SGSRMNQVKDTLQIFLR	313
Mus musculus P4	856	ELADVLPDLRG--------KNEVIICLDCSSSM---------EGVTFTQAKQVALYALS	897
Homo sapiens P4	863	DLDVDLPDLAE---------SEVIICLDCSSSM---------EGVTFLQAKQIALHALS	904
Danio rerio P4	931	DFKSSGLSLSGGSSSV----SDVVILLDSSRSM---------QGEAMLNARRIALQVLK	976
Amphimedon queenslandica	271	QFPALSDSTQ----------CEFVFLVDRSGSM---------SGRYIKSASETLVLFLK	310
Mus musculus I1	280	PKNLTNMS-----------K-NLVFVIDISGSM---------EGQKVRQTKEALLKILE	317
Danio rerio I4	261	PSDVPRIP-----------K-NVVFIIDRSGSM---------HGRRIRQTRSALLTILK	298
Crassostrea virginica I	258	PEGIEPMP------------MDIIFVLDKSGSM---------GRTKMDQLKESMMKILG	295
Homo sapiens I1	283	PQNLTNMN------------KNVVFVIDISGSM---------RGQKVKQTKEALLKILG	320
Folsomia candida	303	SFALNEE------------KVELIILVDRSGSM---------GGYSIRMASAALQLFLH	340
Daphnia magna	288	SFKLAEQ------------KTELIFLVDRSGSM---------DGQGIEQAKQALKLFLH	325
Neurospora crassa	286	KFNLPSTR------------PEIVFVCDRSGSM---------GGARIEGLKSALRIFLK	323
Tuber magnatum	288	KFVLPPQG-----------H-EIVFVVDRSGSM----------YNKIATVRSALQLFLA	324
Rhizophagus irregularis	268	TFALNATM------------SELIFVVDRSGSM---------GIEPMKKAAQALELLLH	305
Penicillium solitum	282	KFNLRPAS------------PEVVFVIDRSGSM----------GQKIPTLRSALQVFLK	318
Vitrella brassicaformis	333	KFTLDEQ------------KCELVFVLDRSGSM---------GGSRIEQSKKAMTIFLK	370

. vWFA .

Tepidicaulis marinus I	388	RLTPQDR----FNVVRFNHTHETLFPQAVRA-DKENLDRALAFVRGLNAE-GGTEML	438
Halopseudomonas pelagia	359	RLQPEDR----FNVLEFNTHHRLLFRDLVAA-DEENIIQARQWVRELRGD-GGTNML	400
Dunaliella salina	358	DLPIAEGI--KFQIVLFGSSHKTMSTTSVDYDAQAEQHAVTWINTNVNPNFGGTEIY	412
Papaver somniferum I	205	NLNQEDS----FSLIALSGETFLFSSTLQLA-TTETVEKATLWIQTNFVAAGGTNIL	256

```
Coffea arabica I               355 KLDSEDL----FNVIAFNGETYIFSSTLVPA-TTEAIEKVTEWINMNLIAGGSTNIL   406
Populus alba                   362 KLNPEDS----FNIIAFNVETYLFSSLMEQA-TKEAILQATQWLNDKLTADGGTDIL   413
Physcomitrium patens I         365 SLKPEDS----FNIIAFDHDLTLFSPQMERS-TTTSIARACEWSMTNCTARGGTDIL   416
Arabidopsis thaliana           355 KLQAEDV----FNIIAFNDEILEFSTSMEFA-TDETISAVTEWLDSNLIANGGTNML   406
Chenopodium quinoa             357 TLNQDDL----FNIIAFNGDSHLFSSSMELA-THEVLKRVSEWISIKLLPEGETNLL   408
Tetrahymena thermophila        355 SLPLDSY----FNIYSFGTEFSKLFDQSQKY-SNENVELALNEIITYSANYGGTNIY   406
Physcomitrium patens V         308 SIPCEDH---YFNIIGFGDNHKTLFPKSTPY-NEETLTKGLRYAQALEADMGGTEMM   360
Chlamydomonas reinhardtii      344 DLPVDSGV--RFNMTVFGSSHNSLYSNCKPYDSKTEKEAVAWIQSKVHANLGGTEIL   398
Trichomonas vaginalis          274 SLPLGCR----FSIIKFGTSFETVLQPCDYS--DENVDKALNLLKSVNAKMGGTDIL   324
Paramecium tetraurelia         378 SLPEDCN----FNIISFGSTFRSLWSESKQY-SQNTLEDAIKHVNNMEANMNGTEIL   429
Homo sapiens VB                390 SLMPACL----FNIIGFGSTFKSLFPSSQTY-SEDSLAMACDDIQRMKADMGGTNIL   441
Mus musculus VB                382 SLMPACF----FNIIGFGSTFKAVFASSRIY-NEENLTMACDCIQRMQADMGGTNML   433
Danio rerio VB                 373 SLFPACL----FNIVGFGSKFKTLFSTSQSY-DEESLALACEYVKKIRADMGGTNIL   424
Heterostelium album P          964 NLRGMNIK---FNIVQFGDIFDWLFIEHVPP-TEANLQLAWSHINSLRPSYGGTALH  1016
Dictyostelium discoideum P    1059 NLRGMQKV--LFDVVCFGDTFDWLFGIGVPP-TESNLQIAWSHINHLKTSYGGTLLH  1112
Acanthamoeba castellanii P     987 LLPKGTAHNYRFNIYVFGSGFESLFA----------------------ADLGGTDLF  1021
Trichoplax adhaerens           550 NLPSNCL----FNIAVFGTSHNELFPCSQQV-AKDTINIAQQFIMTLSATWGDSQFF   601
Crassostrea virginica P        908 HINPSWR----VNVVSFGTAFSELFPSSQPA-NKFNKQAARQFIQKLQATQGNTELC   959
Branchiostoma floridae P       826 HLPKECS----FNVVAFGTGYEELFAVSQSR-TKSNVSKAETFIQNLKASKGNTDAW   877
Entamoeba invadens             250 QLPENST----FDVVSFGSDFKELFGKLEVY-GEKSFKLASEKVAQFEADFGGTNIL   301
Entamoeba dispar               242 QLPLKSK----FEIISFGSKYDFMFKEMVEY-NEDTLKIASDTISEFEANYGGTSMD   293
Heterostelium album V          327 SLTEKSK----FNIWLFGSSHKSLFPTSQIY-SDATLESASAYISKIDANLGGTELY   378
Mus musculus V                 319 SLPMGCY----FNIYGFGATHEEFFPDSVMY-NQETMQIAVKKVKRLLADLGGTELL   370
Homo sapiens V                 335 SLPIGCY----FNIYGFGSSYEACFPESVKY-TQQTMEEALGRVKLMQADLGGTEIL   386
Branchiostoma floridae V       304 SLPIGCY----FNIVGFGSTHESLFKGSEKY-DNKSLKTACKALGKMEADLGGTEIL   355
Crassostrea virginica V        321 SLPVNCI----FNIVSFGSTYKSLFSKSKQY-NEENLMKALKLQKSMSANMGGTEIF   372
Danio rerio V                  316 SLPLGCY----FNIYGFGSSFQAFFPQSVLY-SEQTLQEALQRVKLMRADLGGTEIL   367
Cavenderia fasciculata         328 SLTENSK----FNIVLFGSNFKSLFPESMPY-DDANLEIASTYIQKIQADLGGTELL   379
Tieghemostelium lacteum        304 SIPENSK----FNIYRFGSTFKKLYETSKLY-NNLTLQEATNLLNNTSADLGGTDLL   355
Dictyostelium discoideum V2    375 SLNEQCK----FNIYCFGSGFNKAFQEGSRKYDDDSLAVVNRYVSNISANLGGTELL   427
Dictyostelium discoideum V1    328 SLNEQHK----VNIYCFGSSFNKVFDKSRVY-NDETLEIAGSFVEKISANLGGTELL   379
Polysphondylium violaceum      337 SLTESCK----FNIYCFGSSFKKLFDRSRIY-DDESLETASSYINSIDANLGGTELF   388
Dictyostelium purpureum V      326 SLSEQNK----FNIYCFGSGFNKVFPESLLY-NDESLAMASAYIDNISANLGGTELL   377
Acanthamoeba castellanii V     314 SLGEGTM----FNIIGFGTSTQHLFREGSVEYNDKNLEIATKHVKEMSANLGGTNIL   366
Mus musculus P4                898 LLGEEQK----VNIMQFGTGYKELFSYPKCI---TDSKMATEFIMSAAPSMGNTDFW   947
Homo sapiens P4                905 LVGEKQK----VNIIQFGTGYKELFSYPKHI---TSNTMAAEFIMSATPTMGNTDFW   954
Danio rerio P4                 977 SLARSLK----INIISFSTDYKEAFPAPVPL--NEASEAARKFIMSCSGAGGSTDLW  1027
Amphimedon queenslandica       311 SLPEGCY----FNIYGFGSRYVSLFSTSVPY-NQKNLEKAIDHAQSLKADLGGTEIL   362
Mus musculus I1                318 DMRPVDN----FDLVLFGSKVQSWKGSLVPV-SNANLQAAQDFVRRFSLA-GATNLN   368
Danio rerio I4                 299 DLDEDDH----FGLITFDAEIDFWKRELLQA--TANRENAESFVKRIQDR-GATNIN   349
Crassostrea virginica I        296 DVKAEDK----IFIMAFESSLHNWKPEFVQA-TPENIIASKNYVNNIKSG-GGTNID   346
Homo sapiens I1                321 DMQPGDY----FDLVLFGTRVQSWKGSLVQA-SEANLQAAQDFVRGFSLDE-ATNLN   371
Folsomia candida               341 SLPTDCY----FSVIGFGSSYELLFKEGSLKYGPESLHETVNYAKSISANLGGTEIL   393
Daphnia magna                  326 SLPLDCY----VNIAGFGFSYEELYPSSKKY-DEGVLNTAKAYAEQLRANLGGTEIL   377
Neurospora crassa              324 SIPVGAK----FNICSFGSTFEFLFSDGSRSYDHESLRLAMDYVSRMDADLGGTEMY   376
Tuber magnatum                 325 SLPVGSY----FNMCSFGSNYSSLWPQSRKY-SEESLSVAKAHVMGFDSNMGGTEIL   376
Rhizophagus irregularis        306 SLSEDCY----FNVVSFGSRYDPLFPKSQLY-SETSLSKALNLAQTMTANYGGTEVF   357
Penicillium solitum            319 SLPVGIC----FNICSFGSHYSFMWPTSKVY-DASSLKQALDFVDTVEANMGGTEMR   370
Vitrella brassicaformis        371 SLPMDCY----FSFISFGSSFSVWKPSSVKS-SEATVAEALAFVSTVDADMGGTEIL   422
```

```
                                   .                        vWFA                        .
                                   ______________________________________________________

Tepidicaulis marinus I         439 GALQQALIAQ-------PADSQYLRQVVFLTDGAVGN-----EEQLFNTISQQLG   481
Halopseudomonas pelagia        401 PVLHDAMAFP--------HDSDYLRQIIFITDGSVSN-----EAAILDMIDRQIH   442
Dunaliella salina              413 KTMRAVYDTP--------IPEGYERQIIFLTDGGAMGDE-NFTEKLLQKHEQAAK   458
Papaver somniferum I           257 LPLSQAMEML-----SNIFDSDSISHIFLITDGSVENEKHICELIRNQVAAKGSR   306
Coffea arabica I               407 LPLTQAMEML-------SDTKSSVPIIFLITDGAVEDERRICDVMKHRLMDKRKM   454
Populus alba                   414 APLKQALKLL-------AETTDSIPLIFLITDGAVEDERDICNFVKGSLTSGGSI   461
Physcomitrium patens I         417 SPLQQAFQLL-------ENFPGAIPYVFLITDGAVSAEQNICLTMQSRIVALGAR   464
Arabidopsis thaliana           407 LPLKQAMKLL-------EGSNIGVPLVYLVTDGSVENEREICHAMKESCSRNGKS   454
Chenopodium quinoa             409 LPMKQALEMV-------SHDSSSMAHIFLITDGAVENERDICNVVQDYCATKEKS   456
Tetrahymena thermophila        407 QPLSEIFNQP--------YVKGYGRQIYILTDGQIEN-----KENVMHLIQSNNI   448
Physcomitrium patens V         361 SAFEEIFEHR---------RRDVPTQIFLLTDGEIWDVDS--LIECIRDAKKEEK   404
Chlamydomonas reinhardtii      399 GTMQHIYNSP--------IAAGYTREIIFLTDGGISGH---EEQAVYDLVNPKAK   442
Trichomonas vaginalis          325 SPLQHIAGLK--------PQPGFVKQIFLLTDGEVNN----PDITCATALKNRNE   367
Paramecium tetraurelia         430 KPLSQVVYSK-----YYGKSKSTTLNVFLLTDGEVE------AQPIIDLVKKNNQ   473
Homo sapiens VB                442 SPLKWVIRQP--------VHRGHPRLLFVITDGAVNN-----TGKVLELVRNHAF   483
Mus musculus VB                434 SPLKWVLRQP--------LRRGHPRLLFLITDGSVNN-----TGKVLELVRNHAS   475
Danio rerio VB                 425 APLNWILRQP--------MHRGHPRLLFLLTDGAVSN-----TGKVIELLRSHAR   466
Heterostelium album P         1017 LPLQSLILMS------DQTPSNRPHNIVLFTDGQIAN------PPLVQALIRRSS  1059
Dictyostelium discoideum P    1113 QPLQSLYLLA------EKAKPTNPHNILLFTDGNVAN------EELVQMLVKKAS  1155
Acanthamoeba castellanii P    1022 APLRALHLLSGAGA-SATAHTKPPRNVFLFSDGQIND-----EEHVAHAIAQHRQ  1070
Trichoplax adhaerens           602 NILDNYHHIA------KGLKSNGAYNLFVISDGHFP-----AENSIIDITRKEIN   645
Crassostrea virginica P        960 RPLNSYYLLK---------PESSTGNIFLLSDGHINN----EETTLAKISQNFQH  1001
Branchiostoma floridae P       878 RPLRGFFLLP---------PSGKTRNVFLISDGHVNN----PDQTLRDIHQHYQE   919
Entamoeba invadens             302 KPLCWAMKRK--------------CQVILLTDGQISE---TEETQILNLLEEEKG   339
Entamoeba dispar               294 APLKALID-----------NNTEKCHIILLTDGYVDN-----KINTIEYIHNLSK   332
Heterostelium album V          379 APLKAIFAQA--------YDPQYPRQLFILTDGEISD-----RDRTIDLAGKDSL   420
```

```
Mus musculus V                 371 TPLRKIFRKP--------PIPGHPLQVFVFTDGEVVE-----TFSVIREVMFQSK   412
Homo sapiens V                 387 APLQNIYRGP--------SIPGHPLQLFVFTDGEVTD-----TFSVIKEVRINRQ   428
Branchiostoma floridae V       356 QPLQYVYKQP--------PIAGHPRQLFLLTDGEVWD-----TQACVREVAKHAD   397
Crassostrea virginica V        373 KPLKNVFKNK--------PSASYSRQIFLLTDGMVY-----TVPKIVDLVRKQKN   414
Danio rerio V                  368 QPLQHIYRQA--------CIPEHPRQLFIFTDGEVWN-----TRELLDLVRAHSS   409
Cavenderia fasciculata         380 PPIKSILSKP--------YDPQYPRQVFILTDGEVSE-----RDQLIDFVGKEAN   421
Tieghemostelium lacteum        356 PSVRDIFQQP--------YDPEYPRQIFVLTDGGVSD-----RASLISFVRNEPH   397
Dictyostelium discoideum V2    428 QPIKDILSKEI--------DPEYPRQIFILTDGAVSD-----RSKLIEFVSKESK   469
Dictyostelium discoideum V1    380 PPMVDILSSP--------NDPEYPRQVFILTDGEISE-----RDKLIDYVAKEAN   421
Polysphondylium violaceum      389 PPVRDILSAQ--------ADSEYPRQVFILTDGEISE-----RDKLIDYVGKEAD   430
Dictyostelium purpureum V      378 PPIKDILGKD--------SDAEYPRQVFLLTDGAVSA-----RDQLIDYVGKEAN   419
Acanthamoeba castellanii V     367 RPLQEVLRAQ--------TKEGYPRQLFILTDGEVGN-----TQECVDFVRKHAE   408
Mus musculus P4                948 KVLRYLSLLY---------PSEGFRNILLISDGHLQS-----ESLTLQLVKRNIQ   988
Homo sapiens P4                955 KTLRYLSLLY---------PARGSRNILLVSDGHLQD-----ESLTLQLVKRSRP   995
Danio rerio P4                1028 RPLRSLSLLP---------PCQGMRNVLLLSDGHVQN-----QPVTLQLVRENSC  1068
Amphimedon queenslandica       363 PPLRDIYKKD--------PIKDFTRQIFLLTDGSVSN-----TTECIDEVKRNVN   404
Mus musculus I1                369 GGLLRGIEILNKAQGSHPELSSPASILIMLTDGEPTEGETDRSQILKNVRNAIRG   423
Danio rerio I4                 350 DAVLAGVDMINRNP-----RKGTASILILLTDGDPTAGETNIEKIMANVKEAIGS   399
Crassostrea virginica I        347 LALKEGIKRLT----QISGNKGRAPVLVFLTDGEATVGETNTERILENLKRENEA   388
Homo sapiens I1                372 GGLLRGIEILNQVQESLPELSNHASILIMLTDGDPTEGVTDRSQILKNVRNAIRG   426
Folsomia candida               394 APLKDIYSRP--------HIRGYLRQIFVITDGAISN-----TDQVISLVKQNAH   435
Daphnia magna                  378 QPLERIFKKP--------PIDGYLRQVFVLTDGEVSN-----APQVLSLVRQHSG   419
Neurospora crassa              377 QPLEAAFEKR---------YNDMDLEVFLLTDGEIWN-----QEHLFTMINKKVS   417
Tuber magnatum                 377 APVMDTMRRR--------RPDMRTEILILTDGEVWD-----TERMFEFINGSSK   417
Rhizophagus irregularis        358 SALKWTFENS---------RDDMPTSVFFLTDGGVWNVD-----QIVELVRENEE   398
Penicillium solitum            371 GAVMATVQNR---------LNFKYLDVLLLTDGQIHD-----QDRLFDFVREKAA   411
Vitrella brassicaformis        423 KPLEHLFKKM-------KPTQGYARQVFVLTDGQVSN-----EAEVVQLVKTEAN   465

                                   .                         vWFA                         .
Tepidicaulis marinus I         482 AS----------RLFTVGIGSAPNSFFMSRAAEMGRGTFTHIGEPEQVK--ERMAQLF   527
Halopseudomonas pelagia        443 RA----------RLFTVGIGAAPNSYLLRKAAEMGRGGYSYIAHSHEVE--QQMARLF   488
Dunaliella salina              459 T-----------TVQCLGIGHGVHRKLVDTMASTTGGLSQFAMSAQDIVPACSFLKKC   505
Papaver somniferum I           307 FP----------RISTFGIGIHCNHYFLQMLALISKGHYGAALHPDHIE--IQFQRLF   352
Coffea arabica I               455 FP----------RIYTFGIGSFCNHYFLRMLAMIGRGHHDASYDADSIDV--RIEGLF   500
Populus alba                   462 SL----------RISTFGIGTYCNHYFLRMLAQIGRGHFDTAYDADSVD--FRMQRLF   507
Physcomitrium patens I         465 AP----------RISTFGIGHYCNFYFLKMLAVIGRGMNEVAFKSDKIR--GQMERML   510
Arabidopsis thaliana           455 ISP---------RISTFGIGSFCNHYFLQMLARIGNGYYDGTNNTDSFEH--QMSRLF   501
Chenopodium quinoa             457 PFL---------RVSTFGIGSYCNHFFLQTLAQIAKGHYDAAYDAGSIAP--QLQRLC   503
Tetrahymena thermophila        449 SN----------RVHAIGIGLYVDKDLIIQSAKSGKGCHAHVTDQSLIQE--SIINIL   494
Physcomitrium patens V         405 SDNFV-------RVFSLGIGSNVSHHLVESVGRAADGYAQLIVEGERME--KKVINML   453
Chlamydomonas reinhardtii      443 APVPAAART---HVLSLGIGHGVHRSLLDGMSTRSDGAVVYVVDDEAIAAKTAFLKKA   497
Trichomonas vaginalis          368 N-----------RIFSIGLGSGADPGLIKGLAKKSGGNYIMIADEDNMNE--KVITLL   412
Paramecium tetraurelia         474 AET---------RVYTLGIGEGCSQFLIKRLAEVGNGKFQFVSDNEDIN--AKVIDLL   520
Homo sapiens VB               484 ST----------RCYSFGIGPNVCHRLVKGLASVSEGSAELLMEGERLQ--PKMVKSL   529
Mus musculus VB                476 ST----------RCYSFGIGPTVCYRLVKGLASVSKGSAEFLMEGERLQP--KMVKSL   521
Danio rerio VB                 467 FT----------RCFTFGIGQAACRRLVSGLSAVSRGTAEFLAEGERLQP--KMIKSL   512
Heterostelium album P         1060 SKC---------RLFCFGVGPDVSRHLIKSLTRLGAGFAEFITPNKRPST-KKIIAQL  1107
Dictyostelium discoideum P    1156 PYC---------RMFAFGIGEHCSRHFVKSICRLGGGYPEFIQTNKRPNP-KKIIDQL  1203
Acanthamoeba castellanii P    1071 RL----------RLFTFGFGANASRHTLRTLARVGAGAHEFM-EAGALPNRSKIERQF  1117
Trichoplax adhaerens           646 CL----------RIFTFSIGNVCNRYFMRLLAKVGAGYHEHFDSNLKSKWNSKIKKQI   693
Crassostrea virginica P       1002 T-----------RVFTMGISAVANRHLLKALARVGAGSFEFLDSKFKSKWEDKVKSQL  1048
Branchiostoma floridae P       920 T-----------RVFSCGVGSTSNKHLLRALARVGGGAFEYFDDKTKSKWEKKVKSQL   966
Entamoeba invadens             340 KS----------IVHCIGLGSDVDGNLIRKIGTIGGGVFDIVRNTENLT--KSLSEIT   385
Entamoeba dispar               333 KN----------SLHGVGLGRSCDIELIRNIGRIGNGISVISKNVNLLK--KEVSKIT   378
Heterostelium album V          421 TT----------RIFTLGIGSGVDRNLVVGLSKSCKGYYDFIDSNTEME--NRVMKLM   466
Mus musculus V                 413 KH----------RCFSFGIGEGASTSLIKNLARVSGGTAEFITGNDRMQ--SKALRSL   458
Homo sapiens V                 429 KH----------RCFSFGIGEGTSTSLIKGIARASGGTSEFITGKDRMQ--SKALRTL   474
Branchiostoma floridae V       398 SA----------RCFSVGIGEGASTALVKGVARAGRGKAEFVSGTDRLQA--KVMRLL   443
Crassostrea virginica V        415 T-----------RIFTFGIGDGCSTQLIRDVAKAGNGKPTFVKDNDRLQS--KVMSVL   459
Danio rerio V                  410 SH----------RCFSFGIGEGASTALITGMAKEGSGHAQFITGSDRMQP--KVMQSL   455
Cavenderia fasciculata         422 TT----------RIFTLGIGSGVDRELVIGLSKSCKGYYEFIEENSMMET--QVVKLM   467
Tieghemostelium lacteum        398 V-----------RMFTFGIGSGVDIDLVTGLSQASRGHYELITDINTMEA--KVLKLL   442
Dictyostelium discoideum V2    470 TT----------RIFTYGIGSSVDVELVVGLSKACKGYYTLIRNSSDME--TEVMKLL   515
Dictyostelium discoideum V1    422 TT----------RIFTYGIGASVDQELVIGLSKACKGYYEMIKETTNMEK--QVMKLL   467
Polysphondylium violaceum      431 TT----------RIFTLGIGSGVDRELVVGLSKSCKGFFELIDDNKDME--AKVMSLV   476
Dictyostelium purpureum V      420 TT----------RMFTFGIGTSVDKELVIGLSKACKGFYEFILDNGDMED--KVMKLL   465
Acanthamoeba castellanii V     409 TT----------RVFTFGVGNEASQDLVKGLAKAGEGFFEFVRSGEAMEE--KVMRQL   454
Mus musculus P4                989 HT----------RVFTCAVGSTANRHILRTLSQCGAGVFEYFNSKSKHSWKKQIEAQM  1036
Homo sapiens P4                996 HT----------RLFACGIGSTANRHVLRILSQCGAGVFEYFNAKSKHSWRKQIEDQM  1043
Danio rerio P4                1069 HT----------RLFTCGLSLTANRHMLRALAQAGGGTYEFFDTKMKHTWAEKVRAQV  1116
Amphimedon queenslandica       405 IA----------KCFTFGIGSGASSALVEGMASAGDGTAEFVKEGERLQP--KVIRSL   450
Mus musculus I1                424 RF----------PLYNLGFGHDLDFSFLEVMSTENNGWAQRIYEDHDAT--QQLQGFY   469
Danio rerio I4                 400 KF----------PLYCLGFGYDVNFDFLTKMSLENNAVARRIYEDSDADI--QLQGFY   445
Crassostrea virginica I        389 EI----------PIFSLAFGQGADFDIVKRVAAQNNGFGRKIYEDSDAAL--QIAGFY   434
Homo sapiens I1                427 RF----------PLYNLGFGHNVDFNFLEVMSMENNGRAQRIYEDHDAT--QQLQGFY   472
Folsomia candida               436 NS----------RLFALGIGPSASHYLVQGIATAGGGTCAFVEGDDSIQ--NATLSQL   481
Daphnia magna                  420 RT----------RVFALGLGSSASHHLVEGMARAGNGTALFASLEERLEK--KVMQQL   465
```

```
Neurospora crassa             418 ESQGAI------RLFTLGIGNDVSHALIEGAARAGNGFAQSVTDSEKMN--AKVVRML   467
Tuber magnatum                418 DGNV--------RFFSLGVGDAVSHSLVEGISRAGRGYSQIVSTNSNLQK--KVMRML   465
Rhizophagus irregularis       399 KKKDDL------RLFSLGIGDSVSHNLVESIARAGKGYSQFVTNDERID--KKVIGML   448
Penicillium solitum           412 DNTA--------RFFSLGIGKAASHSLIEGIARAGNGFCQSVTEYEELD--RKVVRML   459
Vitrella brassicaformis       466 PSSPSAGEHQPPRVFSLGIGSGVSTYLVKGIARNGNGDAQFVTDTEDLRE--AVIGQL   521


                                  .vWFA..                    Hyb-1b                      .

Tepidicaulis marinus I        528 TKLENPALTDLTAVWPDGQE-VEAWPNPLP--DLYLGEPIILAARMA-------DA   573
Halopseudomonas pelagia       489 EKLERPVLTDLRIELPDGIT-ADYWPEQLP--DLYAGQPLIVAMRLN---------   532
Dunaliella salina             506 ALSEDVLLQPRLKPKLCMLRSVPAVLPPR----LFAGEPLLVLTEIVK--------   549
Papaver somniferum I          353 TTASSTVLANIVVDELNHLDAIEVYPRYIP--DLSSGNPLIVSGRYK---------   397
Coffea arabica I              501 TRASSIILANIALENVGDLDEFEVYPSRIP--DLSSVSPLIVSGRYQ---------   545
Populus alba                  508 ATASSIILANITVDALESLDSLELLPFCIP--DLSCGCPLIISGRYS---------   552
Physcomitrium patens I        511 VATAAPVLTNIGLARLPDNC--EVYPFPIP--DLFCGNPLIISGKFY---------   553
Arabidopsis thaliana          502 EIASSTIVANTTFDALKLLRSVELFPCQVP--DITLGDPLILSGRYK---------   546
Chenopodium quinoa            504 IVSSQVILSNVEVDGLSYVQSLTLYPSMIQ--DLSYGCPIIISGRYE---------   548
Tetrahymena thermophila       495 QNSISPILEDVKLSY------------------NKEIFNSQYPKED-------SLY   515
Physcomitrium patens V        454 KSALVPAVTNVAVQWSE----------------ANGYGDVDIASGD-----------   483
Chlamydomonas reinhardtii     498 ATAAGAALRPRLVARVLPQR-------------VFAGEPLHVLMEVVS--SEPDAA   547
Trichomonas vaginalis         413 SSAIAPAATNISIQTDK--PATEVWPSPCPSVYSNNPQSFLIKAPHS------ENV   460
Paramecium tetraurelia        521 EDSLTPYLKEFNLET------------------NVTNIAQIIPNPE-------SVV   551
Homo sapiens VB               530 KKAMAPVLSDVTVEW------------------IFPETTEVLVSPVSAS--------   560
Mus musculus VB               522 KKAMAPVLSDVTVEW------------------VFPETTEALISPVSTS--------   552
Danio rerio VB                513 KKCMTSVLTDISIEW------------------LYPETKEILLSPVGAT--------   543
Heterostelium album P        1108 QRTVAPAMSNVRVVF------------------DSSDKITQTPATIT---------  1136
Dictyostelium discoideum P   1204 QRLTQPAMSNISVKF-----------------DSSDTNSIVQSPATIT---------  1234
Acanthamoeba castellanii P   1118 KRALQPSLSNISVKW-----------------ITEHAERIQQAPKETS---------  1148
Trichoplax adhaerens          694 EKAFQPTLTSVNVNWQQF---------------GQSYQSNMQAPADIV---------   726
Crassostrea virginica P      1049 QKAAQPVLTSVSVDWR----------------HDDSHAAPIQAPQQIT---------  1080
Branchiostoma floridae P      967 WKAAQPGLTSVSVDWQQW---------------DDDAPPPVQAPNQIV---------   999
Entamoeba invadens            386 AKILRPTISEGKISVENGAN-------------YEEKVPLFAGSFQ----------   418
Entamoeba dispar              379 ERILIPTINKCQIEW---NIKGEIIPKEINN--FYGMITCYIQCKEEIKEGQKIES   429
Heterostelium album V         467 SIAMEPIISNIKVDW-------------------ADLDVIQAPKVVR---------   494
Mus musculus V                459 RRSLQSVVEDVSLSWNL-----------------PPKMFAPMLSPEQT---------   489
Homo sapiens V                475 KRSLQPVVEDVSLSWHL-----------------PPGLSAKMLSPEQT---------   505
Branchiostoma floridae V      444 SCALQPTVTGVGLTWQL-----------------PDGVTAVPIPSTPP---------   474
Crassostrea virginica V       460 RSSMTCGITNVRLDW-----------------NMPEDCSLINVPEEVN---------   490
Danio rerio V                 456 RFALQPAVEELCVDWRL-----------------PEGVSVELLSPPVR---------   486
Cavenderia fasciculata        468 SIAMEPTISNIRVDW-------------------DGLQATQAPSIVR---------   495
Tieghemostelium lacteum       443 RKAMEPMLAHW-----------------------PEGVEVRQAPTLIR---------   471
Dictyostelium discoideum V2   516 SIAFEPTLSNVSFDWSQLLDL-----------SNGKSTTIIQSPTQIR---------   552
Dictyostelium discoideum V1   468 NVAFEPMLSNIKLDW-----------------SSCGLVDVIQAPSHIR---------   498
Polysphondylium violaceum     477 NIAMEPTLSHIKVNW-------------------GELKVKQAPETIR---------   504
Dictyostelium purpureum V     466 SIANEPTLANIKIEW-------------------GDLPVTQSPSTIR---------   493
Acanthamoeba castellanii V    455 HRAMQPALTDITVTW-----------------KGAAASHVQPAPFRLP---------   485
Mus musculus P4              1037 TRIRSPSCHSVSVKWQQL---------------SRDAPEPLQAPAWVP---------  1069
Homo sapiens P4              1044 TRLCSPSCHSVSVKWQQL---------------NPDVPEALQAPAQVP---------  1076
Danio rerio P4               1117 QRMESPGCRSVAVKWQQF---------------NPRAPPPVQAPSQLH---------  1149
Amphimedon queenslandica      451 KHALQPLLSNVKVSFKF---------------SASDDIHVKQVPKTLP---------   483
Mus musculus I1               470 NQVANPLLTDVELQY----------------------PQDAVLALTQHRH------K   498
Danio rerio I4                446 DEVAVPLLTDIQLSY----------------------AGG--KHLTKSS------FG   472
Crassostrea virginica I       435 KEISTVLMKNVSFNY---------------------IDGT--LYDTEVTN----TFS   465
Homo sapiens I1               473 SQVAKPLLVDVDLQY----------------------PQDAVLALTQNHH------K   501
Folsomia candida              482 KNSLQPSLTEINLEWIG-----------------LSSATSVELNPEK----------   511
Daphnia magna                 466 QDALQPALTDIKIKWEG------(52)----SDKPEKKLRQAPCDAM-------IP   533
Neurospora crassa             468 KAGLTPHIKDYTLEIKY------(75)----PPVPEPTILQAPFTIP---------   575
Tuber magnatum                466 KAALTPHVNDWDIEWVG---------------STPSAPSIIQAPTNVP---------   528
Rhizophagus irregularis       449 KNALKSPIKDYNVTWAN------(40)----DIKVQQAPYFIP-------------   517
Penicillium solitum           460 KGALTPHIHDFQLEVEY------(74)----PTITPPRAIRAPYNIP---------   566
Vitrella brassicaformis       522 KRALQPALANLTIEWEG------(67)----LSTSDLPVWRAPFHNP---------   621


                                  .                            Hyb-1b                  .

Tepidicaulis marinus I        574 GTFKLKGTFD-GQPW---------ELRLPLAKAAERPGVAKLWAR-KKIASLE   616
Halopseudomonas pelagia -32-  565 -TLWAKQKIEVLMDR------LSRGESEAQVREDVL-----EVALKHRLLSRY   605
Dunaliella salina             550 --AEKGAALEFSAVR--------ITGEKVSATVPIPDAS-EELPN--VAARA-   588
Papaver somniferum I          398 GEFPDSLKIS-GFLS---------DVSNFTINLKPQRAK-GIIAL-ERVVAKK   438
Coffea arabica I              546 GTFPDALAVS-GIFA---------DISNFSADLKVHHAK--DIPL-HKVQAKQ   585
Populus alba                  553 GNFPDSVKLS-GILA---------DMRKFTIDIKAQKAK--DLPV-DRVVARR   592
Physcomitrium patens I        554 GKFPDSLIVM-GLMP---------DQSTWQIEVPSRNSS--KLPL-NRVFAKQ   593
Arabidopsis thaliana          547 GEFPDEVELR-GTLA---------DMSCFTIELTVQKAK--DIPLDKVLARR-   586
Chenopodium quinoa            549 GKLPESLMVR-GTLA---------DMSNFVTDLKIWKEK--DIPL-DKVFAKA   588
Tetrahymena thermophila       516 CLFKNDLF-TFTLFL-----KSGIDFDSLNDENKLVKI--QYFD--TTINQKV   568
Physcomitrium patens V        484 -HVDGFVIVE-EIAE-----------DEPSQPVAPDSNETDGNTPPINLF---   520
Chlamydomonas reinhardtii     548 LELTADWAEPESAAG------AAPLTLSLPLGPALASAE-EGEAL--PVLHA-   590
Trichomonas vaginalis         461 LISGTCSDETVDIVI---------PVSKCDDNLGMKQLF-ARYII-EDYET--   500
```

```
Paramecium tetraurelia       552 CLKKNQEL-TIQVLF-------SIDQYIDNLQFTLSCFDPQDQKP--IKYE--   592
Homo sapiens VB              561 SLFPGERLVGYGIVC--DASLHISNPRSDK-RRRYSMLHSQESGS-SVFYHS-   608
Mus musculus VB              553 SLFPGERLMGYGIVC--DASLYISNSRSDK-RRKYGMLHTQESSS-SVFYPS-   600
Danio rerio VB               544 CLFPGDHLIGYSVVC--DTSRYHSNPKSDK-RRRYSMMRSNESAS-SVFYHSL   592
Heterostelium album P       1137 SIFRSERQVVYAFSG-------ICTRATLTAQAPGGGLISNAVHT-PEIGF--  1179
Dictyostelium discoideum P  1235 SIFKKERQVMYAFSG-------ICTRATLICQAPGGGLITNVVHT-PEIGF--  1277
Acanthamoeba castellanii P  1149 SLFNGERKMVYSFID-------NCTQAFLHAHNGEQEIET-RVST-SELCF--  1190
Trichoplax adhaerens         727 ALFNGSRQVIYGFIP-------NCLQATLEAEIGGTQIST-VVST-SELSK--   768
Crassostrea virginica P     1081 SLFSGSRQVIYGFVD-------NCYMATLKAEIGGQEIST-VVST-SDLSV--  1122
Branchiostoma floridae P    1000 SLFNGSRQVVYGYVP-------HCTQATLKAVINNREIST-MVST-TELSI--  1041
Entamoeba invadens           419 CYFDVKNDQKC-IAT-------LHGKNG-KKDVKLIEKEVNRIKG--ELLGQM   460
Entamoeba dispar             430 EIKGICREK--EIIY------KNTKNIKITKGIILHQLM-AFNQI-RKLEV--   470
Heterostelium album V        495 PIYSKERMIISGLVN------SIPSNSSLIKNIVITADGPTGEKL-TYEIA--   538
Mus musculus V               490 SIFRGQRLIIYSLLI------GKIPKEEATGEVCLSYKLH-GKSY-EEKVT--   532
Homo sapiens V               506 VIFRGQRLISYAQLT-----GRMPAAE-TTGEVCLKYTL-QGKTF-EDKVT--   548
Branchiostoma floridae V     475 PIFSGDRFIMYAQLQGQVGTHSYLLTAEAILLPELEVNLEKDDNDDSETVS--   525
Crassostrea virginica V      491 TIFPGEKNILYAIVT-------ENVSKINQEKVG-KYSLKVSGEAHYVP----   531
Danio rerio V                487 SLFQGQRALLYGRLR-------GQSSSESDGAVMIKYKLNNTPVTNQLQ----   528
Cavenderia fasciculata       496 PIFNNERMIIYSLID--------NLVEGETPQKTIKLTCDGPTGQ-ELVYE--   537
Tieghemostelium lacteum      472 PIYDQERMMLYGLID-------LKKSSGVKFDETINLKI-TADGPKGDVLT--   514
Dictyostelium discoideum V2  553 PIFNNERMMVYATIE---LDNDISNNIENHGQPVIVTMNA-DGPLG-------   594
Dictyostelium discoideum V1  499 PLFNQERMMIYSMIP---------SNQTNQDIINASIETSKPLII-TLTG---   538
Polysphondylium violaceum    505 PIFFNERMMIYGLLE-------SEPNTEKPHSITITGNGPSGREL-SYTLD--   547
Dictyostelium purpureum V    494 PVFNRERMMVYGILN-------KEPSSISSP-VTIQMVGDGPLGE-QLRFP--   535
Acanthamoeba castellanii V   486 PLFCGGRLVVYGIID-------DSAAAAGGNEAGGGEVEVVIGAK-TAVKP--   528
Mus musculus P4             1070 SLFHNDRLLVYGFIP-------HCTQATLQAFIQEKEFCT-MVST-TELQK--  1111
Homo sapiens P4             1077 SLFLNDRLLVYGFIP-------HCTQATLCALIQEKEFRT-MVST-TELQK--  1118
Danio rerio P4              1150 SLFSDWHTLVYGFVP-------HCTQATLFGDLSGQEIKT-MVST-TELQK--  1191
Amphimedon queenslandica     484 RIFEGERITIYGIVK-------SSDISSPLEGEVTLSGQIITSED-PINVT--   526
Mus musculus I1              499 QYYDGSEIVVAGRIA-------NHKLNTFKADVRARGEK-QEFRA-TCLVD--   540
Danio rerio I4               473 LYFNGSEIVVSGQIT-------DNSVETFTTEVIAVSKG-NNVMYQDTVM---   514
Crassostrea virginica I      466 TYFKGSEMVVAGKVR-------DLNKLQSGLTVNGTGVG-NREIE-IPVPR--   507
Homo sapiens I1              502 QYYEGSEIVVAGRIA-------DNKQSSFKADVQAHGEG-QEFSI-TCLVD--   543
Folsomia candida             512 TLFGYNKPLQPEIIK-------EKTFSQSPKNIPPIFDG-QQMVV-FGIFHP-   554
Daphnia magna                534 PVFDGKHLIVYALLA-------RDAHVPKWAEIIASSPIGPLSLK-ISLNE--   578
Neurospora crassa            576 PLYPFIRTSVYLLLS-----PSTAQKTPKSVILRATSAHGPLELE-IPV----   618
Tuber magnatum               529 ALFPYSRATVYFLIS-------NGDRPEKVFLKGSAHDTNSNTICPLQIE---   571
Rhizophagus irregularis      518 PIYSGVRFIVYCILE-------KNIEPCKVISLKATSQDGPMKLD-IPLDP--   560
Penicillium solitum          567 PLYPFIRTNVFLLMD-------PHSPEKIPKSLKFTATSNDSPLE-LRIPI--   609
Vitrella brassicaformis      622 PVFSGSHYVAYALFH-------DTTKPTHVLLK-ASSPEGPIELR-LPLDD--   663
```

B. Identifiers

Tepidicaulis marinus I	GAK45867
Halopseudomonas pelagia	WP_022961706
Dunaliella salina	KAF5835661
Papaver somniferum I	XP_026450281
Coffea arabica I	XP_027067154
Populus alba	XP_034896162
Physcomitrium patens I	XP_024367213
Arabidopsis thaliana	NP_001320782
Chenopodium quinoa	XP_021720436
Tetrahymena thermophila	XP_001018540
Physcomitrium patens V	XP_024382394
Chlamydomonas reinhardt	XP_042917706
Trichomonas vaginalis	XP_001317686
Paramecium tetraurelia	XP_001451152
Homo sapiens VB	NP_001034589
Mus musculus VB	BAB30227
Danio rerio VB	XP_017209274
Heterostelium album P	XP_020434976
Dictyostelium discoideum P	DDB_G0286613
Acanthamoeba castellanii P	XP_004352905
Trichoplax adhaerens	GFSG01008360
Crassostrea virginica P	XP_022287749
Branchiostoma floridae P	XP_002608764
Entamoeba invadens	XP_004258518
Entamoeba dispar	XP_001738586
Heterostelium album V	XP_020438895
Mus musculus V	EDL25491
Homo sapiens V	XP_011541130
Branchiostoma floridae V	XP_002592482
Crassostrea virginica V	XP_022322840
Danio rerio V	NP_001004604
Cavenderia fasciculata	DFA1591502
Tieghemostelium lacteum	KYQ92222
D. discoideum Vwa2	DDB_G0292740
D. discoideum Vwa1	DDB_G0292016
Polysphondylium violaceum	KAF2069392

Dictyostelium purpureum V	DPU1266017
Acanthamoeba castellanii V	XP_004338797
Mus musculus P4	NP_001139450
Homo sapiens P4	NP_006428
Danio rerio P4	XP_017213366
Amphimedon queenslandica	XP_003383022
Mus musculus I1	CAA49841
Danio rerio I4	NP_001020335
Crassostrea virginica I	XP_022330399
Homo sapiens ITIH1	NP_002206
Folsomia candida	OXA39519
Daphnia magna	XP_032779940
Neurospora crassa	XP_011392876
Tuber magnatum	PWW77351
Rhizophagus irregularis	CAB4480325
Penicillium solitum	XP_040817197
Vitrella brassicaformis	CEM17663

Figure S6. Epitope tagging of Dd*vwa1* and Dd*vwa2* loci and Western blot validation.
Vwa1 **(A)** and Vwa2 **(B)** editing plasmids were designed to insert sequences encoding a $FLAG_3His_6$-epitope tag, a stop codon, and blasticidin S deaminase cassette upstream of their native stop codons, using double cross-over homologous recombination. The editing plasmids were constructed by replacing *culE*-targeting sequences with *vwa1* or *vwa2* targeting sequences (see Methods). See **Table S1** for primer sequences. The plasmids were linearized with BssH1 and electroporated into cells, where the expected insertion is illustrated for Vwa1 (A) and Vwa2 (B). **(C, D)** Western blot screening of transformed clones with anti-FLAG mAb M2. Positive clones used for experimentation are denoted with an asterisk.

A. Vwa1-FLAG3 endogenous tagging strategy

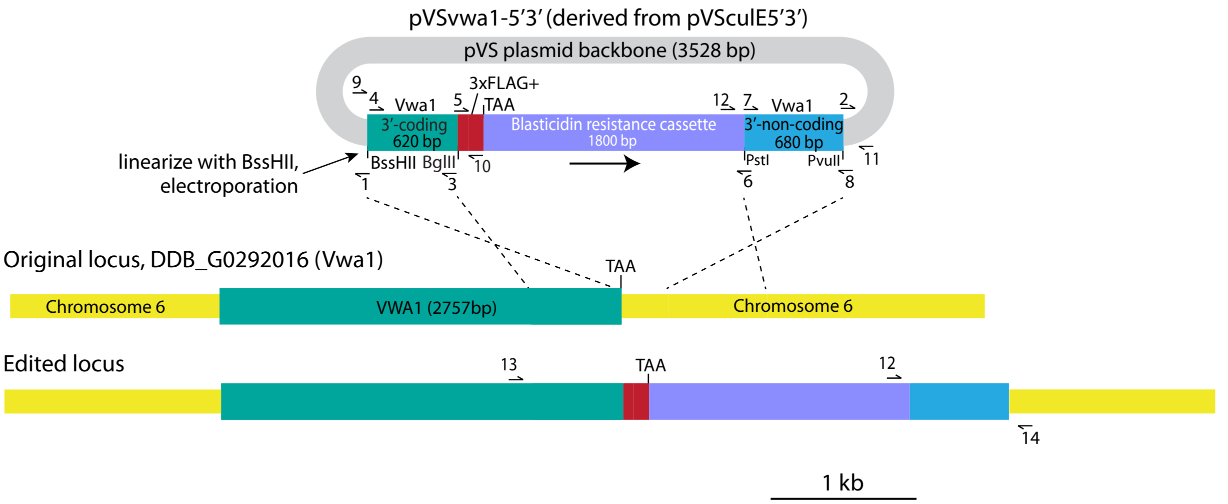

B. Vwa2-FLAG3 endogenous tagging strategy

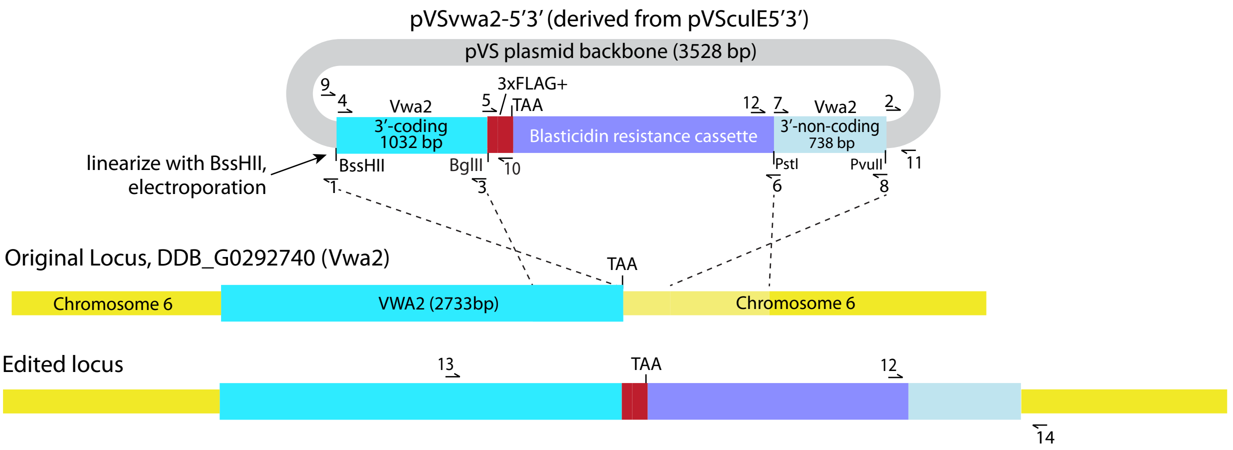

C. Vwa1-FLAG3 clone screen

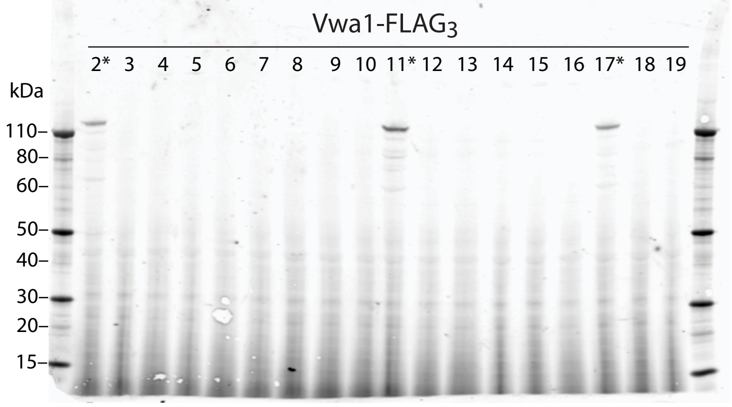

D. Vwa2-FLAG3 clone screen

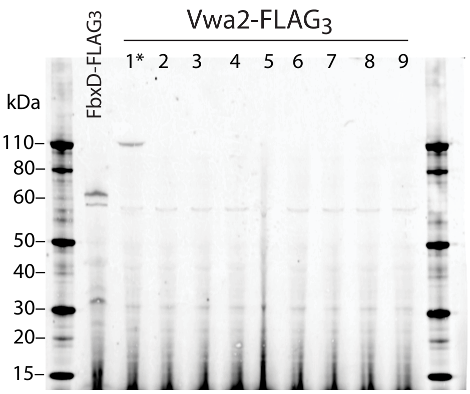

Figure S7. Complementation of the GWDI *vwa1*-disruption strain.
(A) Western blot screening of prespore *cotB*::FLAG$_3$Vwa1 and prestalk *ecmA*::FLAG$_3$Vwa1 expression in slugs of strain GWDI_133_C_2, which contains an insertion in codon-15 of the Vwa1 ORF and referred to here as Vwa1-N2. Clones used for subsequent studies are marked with an asterisk. **(B)** Developmental analysis of Vwa1-N2 cells, complemented derivatives, and the parental normal strain Ax4. Representative spore counts from 3 trials are reported in the boxes in the lower right-hand corner at ×10^7.

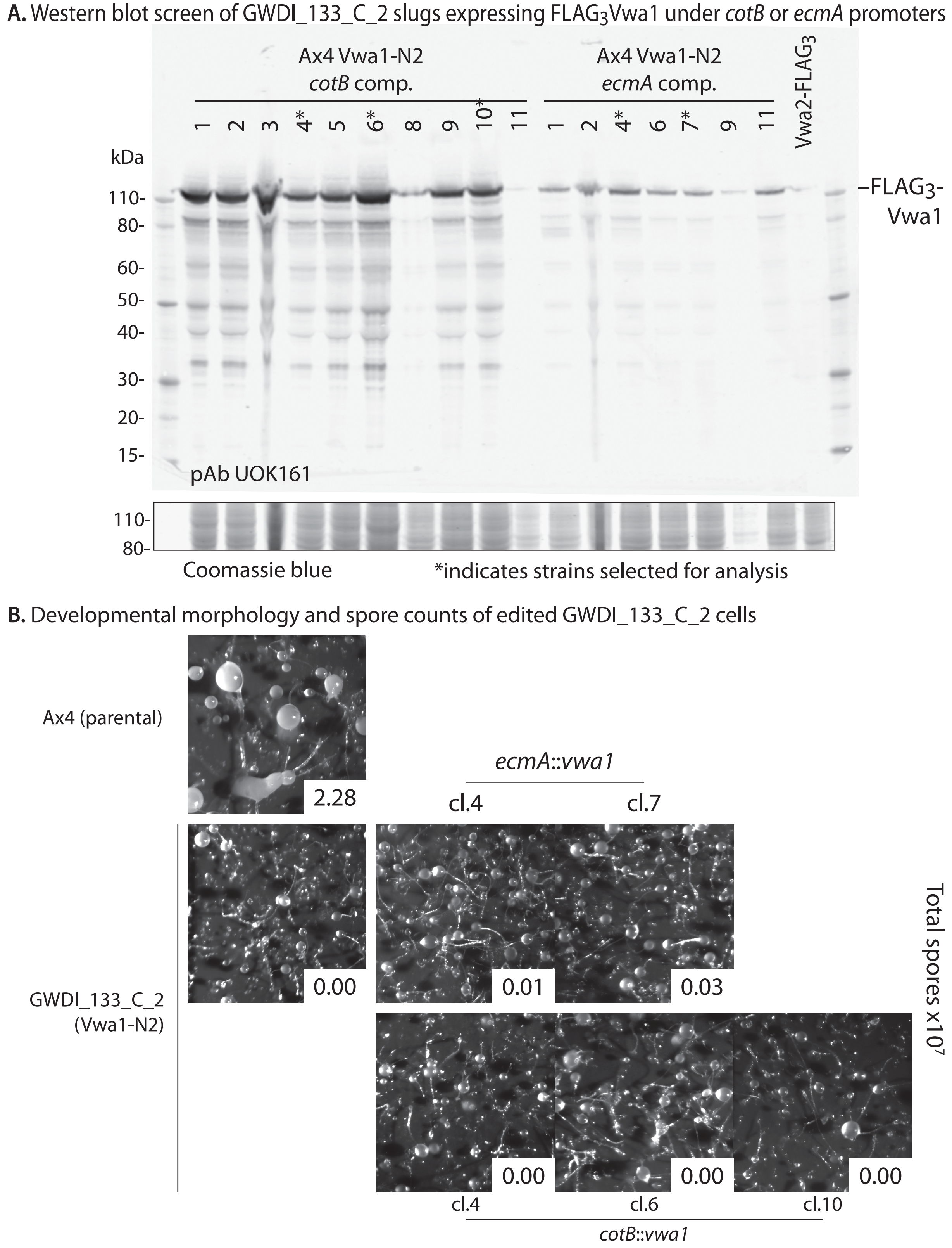

Figure S8. Dd*vwa1* disruption strategy and validation.
(A) Workflow for the stepwise ligation independent strategy for assembling the double homologous recombination construct for truncating the Vwa1 coding region. Insertion of a blasticidin resistance cassette and artificial stop codons in the VIT domain served to truncate the locus. See **Table S1** for oligonucleotide primer sequences. **(B)** DNA gel of PCR validation of desired insertion into the *Dictyostelium* genome. **(C)** Western blot validation of loss of full-length Vwa1 expression in vegetative cells of clones 5 and 11, using anti-Vwa1 pAb UOK162. Asterisks indicate non-specific cross-reacting bands.

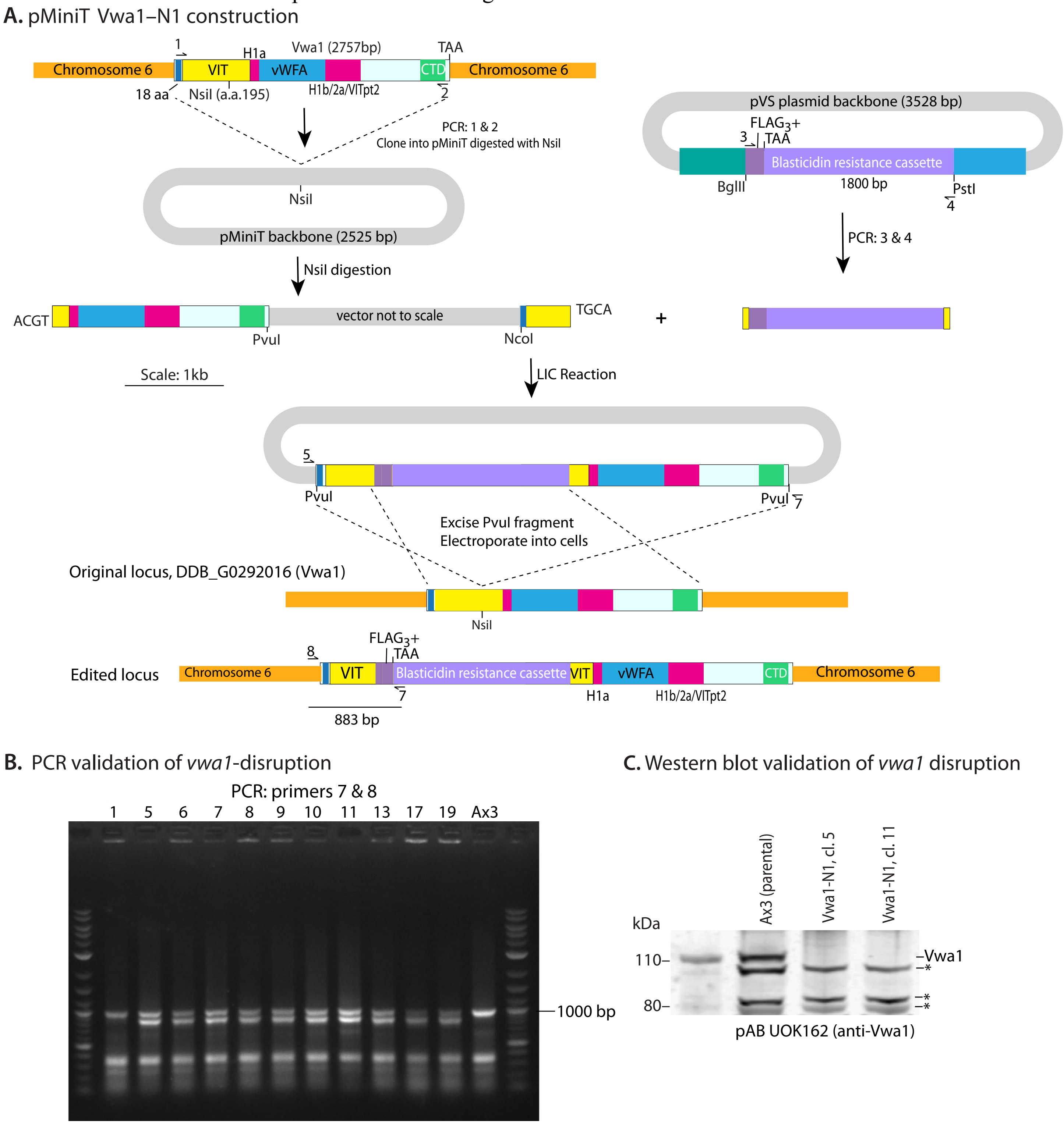

Figure S9. Expression of FbxwD constructs.
(A) The putative RING domain sequence of FbxwD is aligned with highest scoring homologs retrieved using BLASTp in the NCBI nr database, and *D. discoideum* Rbx1 and murine Cbl E3 Ub ligase. Contacts between Cbl and E2 as indicated by a crystal structure of the complex (Zheng et al., 2000) are bold underlined, and informed the choice of point mutations in the FbxwD-RING domain. **(B)** Western blot validation of expression of FLAG$_3$FbxwD-RING mutants. **(C)** Western blot validation of FbxwD expression in the Vwa1-N1 strain. Blots were probed with αFLAG mAb M2 or anti-FLAG mAb 12C6c as indicated. Clones selected for experimentation are asterisked.

A. Alignment of FbxwD RING-like sequences

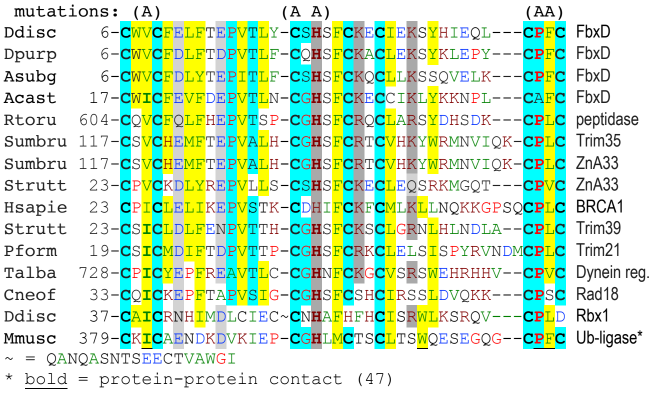

D disc	XP_629697.1	FbxwD, DDB_G0292312, *Dictyostelium discoideum* AX4
D purp	XP_003294190.1	FbxwD, DICPUDRAFT_95983, *D. purpureum*
A subg	XP_012754951.1	FbxwD, SAMD00019534_050260, *Acytostelium subglobosum* LB1
A cast	XP_004356532.1	FbxwD-like, *Acanthamoeba castellanii* str. Neff
R toru	XP_016271080.1	ATP-dependent peptidase, *Rhodotorula toruloides* NP11
S umbru	XP_037611087.1	tripartite motif-containing protein 35-like isoform X1, *Sebastes umbro*
S umbru	XP_037611095.1	zinc-binding protein A33-like isoform X6, *Sebastes umbrosus*
S trutt	XP_029600693.1	zinc-binding protein A33-like, *Salmo trutta*
H sapi	NM_007294	BRCA1 breast cancer DNA repair associated, *Homo sapiens*
S trutt	XP_029616364.1	E3 ubiquitin-protein ligase TRIM39-like, *Salmo trutta*
P form	XP_007574031.1	pred. E3 ubiquitin-protein ligase TRIM21-like isoform X1, *Poecilia formosa*
T alba	XP_032860955.1	dynein regulatory complex protein 1 isoform X1, *Tyto alba alba*
C neof	XP_012053642.1	DNA repair protein Rad18, *Cryptococcus neoformans* var. grubii H99
D disc	XP_637131.1	Rbx1 DDB_G0287629, *D. discoideum* AX4
M musc	NP_031645.2	CBL E3 ubiquitin-protein ligase, *Mus musculus*

B. Western blot analysis of slug FLAG$_3$FbxwD(RING mutant) expression

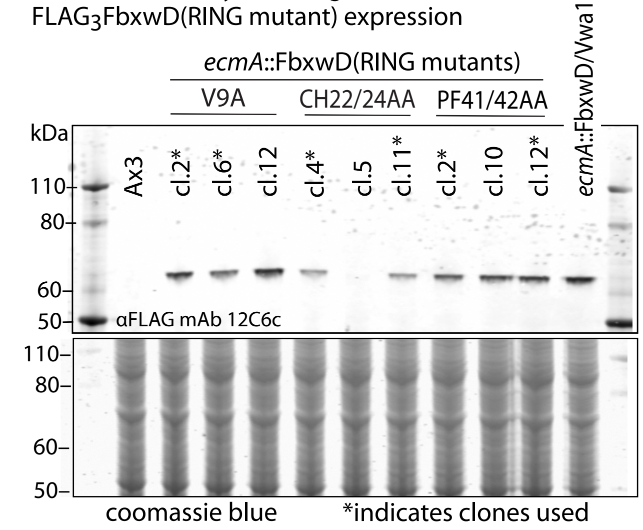

C. Western blot analysis of slug FLAG$_3$FbxwD expression in Vwa1-N1 cells

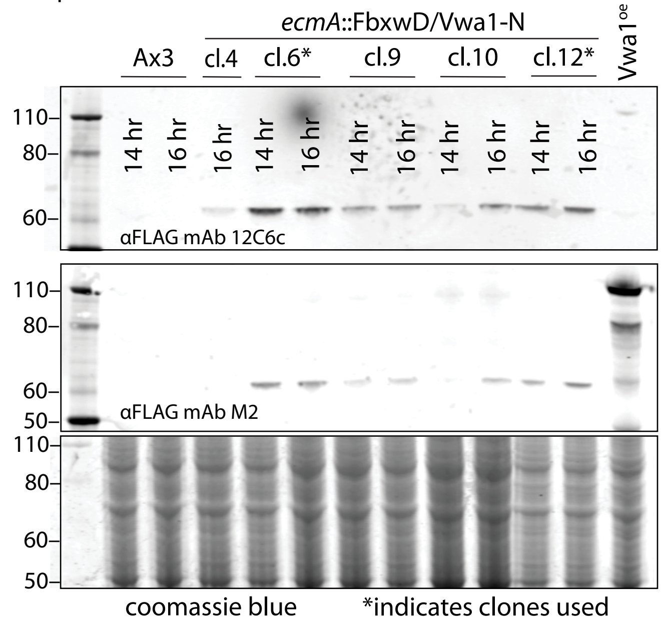

Figure S10. Vwa1 and Vwa2 stability.
Vegetative cells whose Vwa1 or Vwa2 genes were FLAG$_3$-tagged were shaken in HL-5 in the presence of 1 µM Bortezomib, 1 mM cycloheximide, or the DMSO carrier. **(A, C)** Representative Western blots of cells treated for the indicated number of hours. **(B, D)** Densitometry of FLAG$_3$Vwa1 or FLAG$_3$Vwa2 protein level relative to total protein (n=3, ±S.D.).

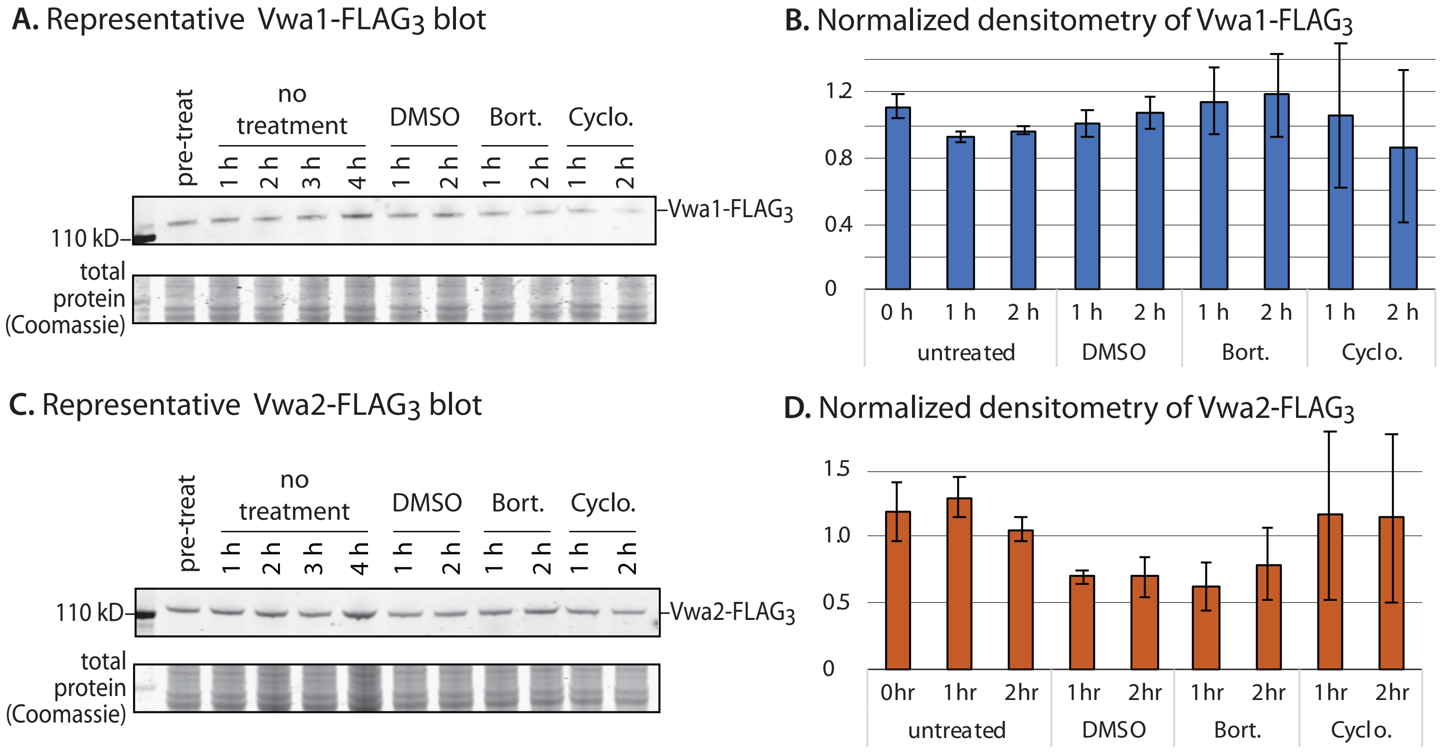

Figure S11. Interactomes of over-expressed Vwa1 domains.
Volcano plots of data from co-IPs from FLAG$_3$VIT, FLAG$_3$-vWFA, and FLAG$_3$C-T expressing strains. Data are plotted as confidence of protein ID (inferred from FDR, ordinate) vs. ratio of presence in the FLAG$_3$-tagged relative to parental (Ax3) strain (spectral counting method, abscissa) from vegetative **(A)** or slug **(B)** co-IPs. Proteins in the upper right quadrant, with abundances 4-fold higher than control (vertical red lines) and with a p-value of <0.01 (horizontal red lines) were considered significant.

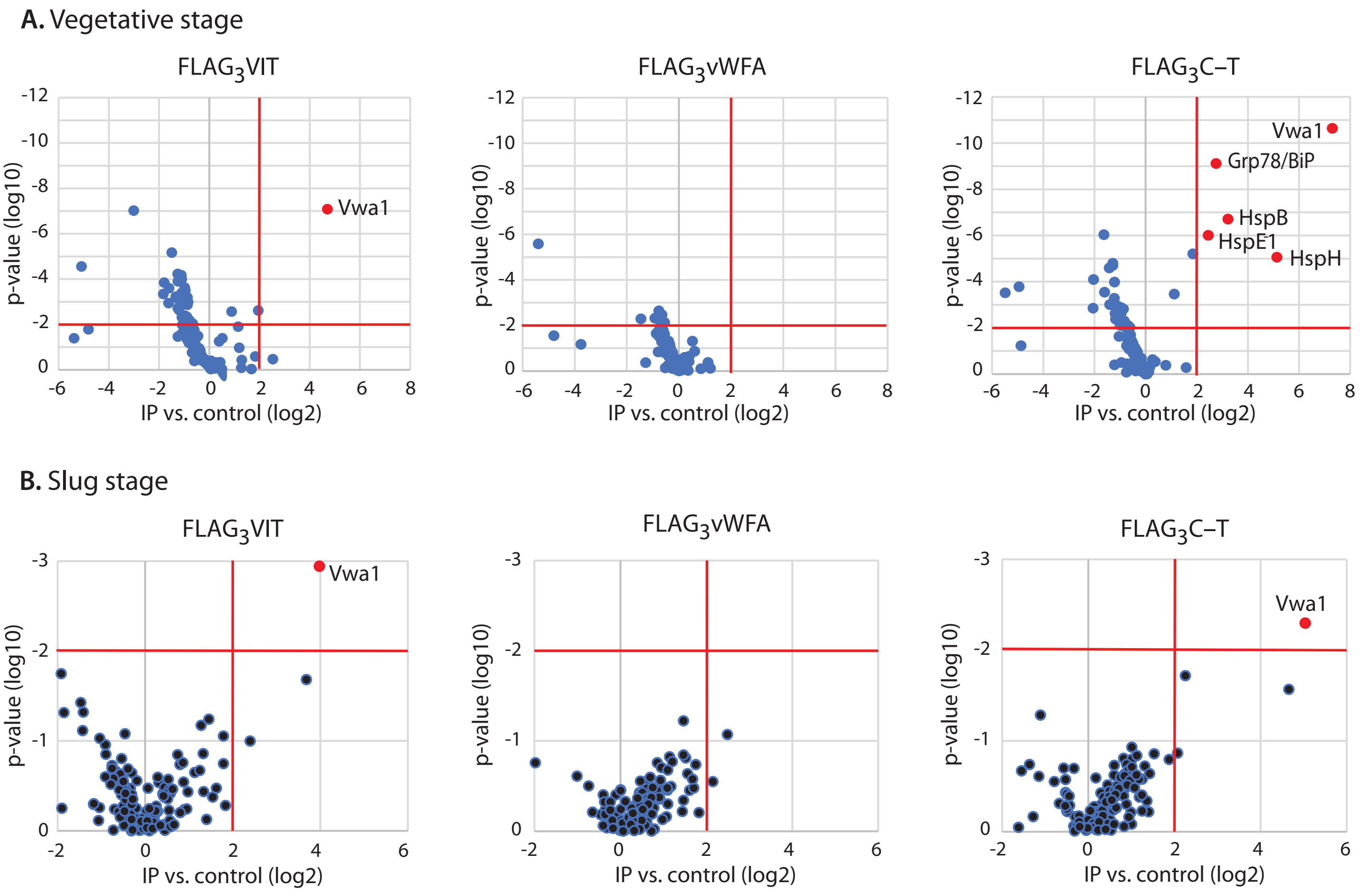

Fig. S12. Development of DdVwa1 mutant strains. (A) Western blot analysis of whole cells for prespore cell biomarkers SP75 and SP96, as detected using mAb 83.5 (1:1000) directed against a fucose-epitope. The Vwa1 constructs were expressed in Ax3 (normal strain) cells and harvested at 10-12 h of development on filters. **(B)** Representative images of strains developing in plaques formed after clonal clearing of bacterial lawns. F.B.=fruiting body.

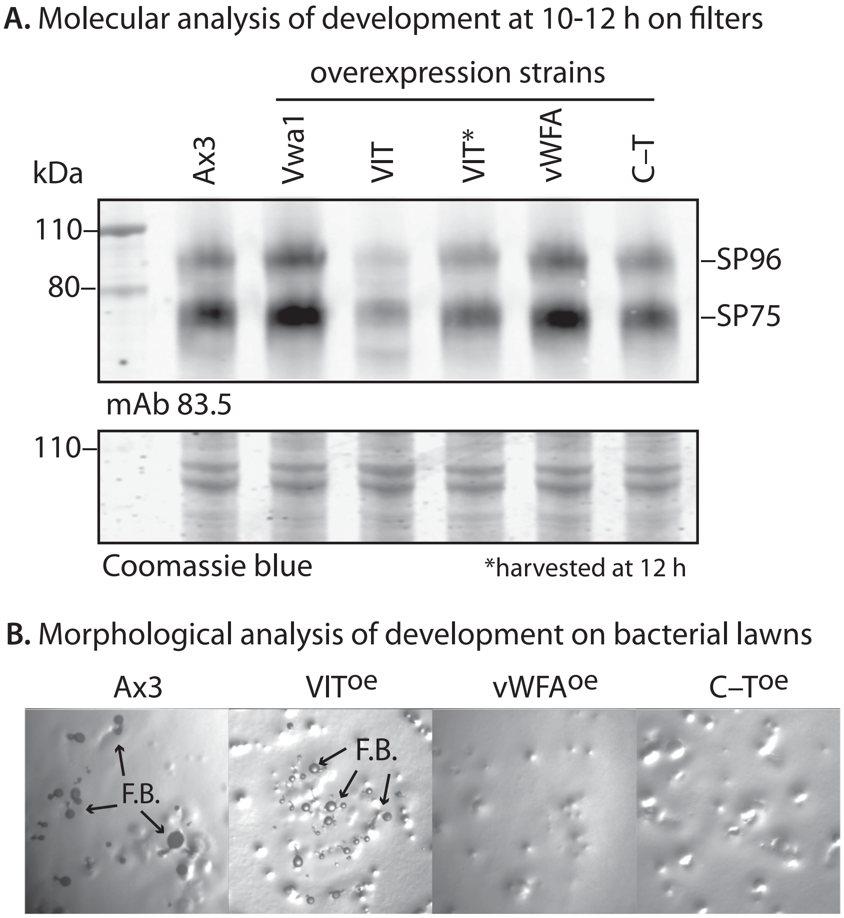

Fig. S13. Sequence of the $FLAG_3UBA_2$ cassette. The nt sequence encodes, in order from the 5'-end, a GS-rich linker, a $FLAG_3$ epitope, a GS-linker, a BirA biotinylation site, a GS-linker, and 2 copies of a ubiquitin-associated (UBA) domain, and is flanked by the indicated restriction enzyme sites. The $FLAG_3$ epitope and biotinylation site sequences were previously described in pVS3 (Sheikh et al., 2015), and the predicted UBA_2 sequence was derived from DDB_G0286357, which encodes RcbA, the predicted ortholog of Rad23 from *Saccharomyces cerevisiae* (Mark et al., 2016).

```
R  S  A  M  E  N  S  G  S  T  G  A  S  G  S  D  Y  K  D  D  D  D  K  D  Y  K  D  D  D  D
AGATCTGCCATGGAGAATTCAGGTTCAACTGGTGCTTCTGGATCAGATTATAAAGATGACGATGACAAGGATTATAAGGATGACGATGAC
BglII   NcoI   EcoRI          linker                                3xFLAG

K  D  Y  K  D  D  D  D  K  G  S  G  G  G  L  N  D  I  F  E  A  Q  K  I  E  W  H  G  S  F
AAAGATTATAAGGATGACGATGACAAAGGTTCAGGTGGTGGTTTAAATGATATTTTTGAAGCACAAAAAATTGAATGGCATGGTTCTTTC
                           linker                    BirA                        linker

A  T  G  T  E  L  E  A  T  I  K  N  I  T  D  M  G  F  A  R  D  Q  V  L  R  A  L  R  L  T
GCCACCGGCACTGAATTAGAAGCCACAATTAAAAATATTACAGATATGGGTTTTGCAAGAGATCAAGTTTTAAGAGCACTCAGACTTACC
Remainder is 2xUBA from C-terminus of D. discoideum RcbA

F  N  N  A  E  R  A  I  E  Y  L  V  S  G  N  I  P  A  A  N  D  P  E  D  E  E  E  M  E  G
TTTAACAATGCTGAACGTGCTATTGAATATTTAGTTAGTGGAAATATTCCAGCAGCAAATGATCCAGAAGATGAAGAAGAAATGGAAGGT
                                                GCT->GCA removes PvuII site

G  G  G  S  G  D  N  P  F  E  A  L  R  N  H  P  H  F  N  L  L  R  E  A  I  S  K  N  P  S
GGTGGTGGTAGTGGTGATAATCCATTCGAAGCTTTAAGAAATCATCCACACTTCAACTTACTTCGTGAAGCCATCTCCAAAAATCCAAGC

I  I  P  G  I  L  Q  Q  L  A  Q  T  N  P  A  L  V  R  Q  I  Q  E  N  P  N  E  F  I  R  L
ATTATCCCAGGAATCTTACAACAATTAGCTCAAACCAATCCAGCCCTCGTTAGACAAATCCAAGAAAATCCAAATGAATTTATTCGTCTC

F  Q  G  D  G  N  P  G  G  N  P  G  Q  F  T  L  Q  V  T  Q  E  E  S  E  A  I  Q  R  L  Q
TTCCAAGGTGATGGTAACCCAGGTGGCAATCCAGGTCAATTTACTCTTCAAGTCACTCAAGAAGAATCTGAAGCAATTCAAAGATTACAA

A  L  T  G  M  D  K  S  T  V  I  E  A  Y  F  A  C  D  K  N  E  E  L  T  A  S  Y  L  F  E
GCCTTAACAGGTATGGATAAATCAACAGTTATTGAAGCTTATTTTGCATGTGACAAGAATGAAGAACTCACAGCTTCTTATCTTTTTGAA

T  A  D  D  E  *
ACTGCTGACGATGAATAAGGATCC (744 nucl)
                   BamHI
```

>DDB0191177|DDB_G0286357 |Protein|gene: rcbA on chromosome: 4 position 4412389 to 4414128

```
MKVTIKNINKEIYVFEVNGDLTVAELKNLISEKHNQTPSWQTLIYSGKILEDKRTLESYNITDSGFIVMMIKKPREAPATTPAPSTTPAPSTTSAPTTT
TTAEPTPTTSSTNNTSTTTPTSVPTPTNNTPATPNPTPTTSSTPGSTSTTSPQQSSDFATGTELEATIKNITDMGFARDQVLRALRLTFNNAERAIEYL
VSGNIPAANDPEDEEEMEGGGGSGDNPFEALRNHPHFNLLREAISKNPSIIPGILQQLAQTNPALVRQIQENPNEFIRLFQGDGNPGGNPGQFTLQVTQ
EESEAIQRLQALTGMDKSTVIEAYFACDKNEELTASYLFETADDE*
Note: A and A are the boundaries for the 2xUBA region based on alignment with yeast Rad23.
```

Alignment:

```
Sc MVSLTFKNFKKEKVPLDLEPSNTILETKTKLAQSISCEESQIKLIYSGKVLQDSKTVSECGLKDGDQVVFMVSQKKSTKTKVTEPPI
Dd MKV-TIKNINKEIYVFEVNGDLTVAELKNLISEKHNQTPSWQTLIYSGKILEDKRTLESYNITDSGFIVMMI--KKPREAPATTPAP

Sc --APESATTPGRENSTEASPS-TDAS-AAPAATAPEG-SQPQEEQTATTERT-ESASTPG------------FVVGTE
Dd STTPAPSTTSAPTTTTTAEPTPTTSSTNNTSTTTPTSVPTPTNNTPATPNPTPTTSSTPGSTSTTSPQQSSDFATGTE

Sc RNETIERIMEMGYQREEVERALRAAFNNPDRAVEYLLMG-IPENLRQPEPQQQTAAAAEQPSTAATTAEQPAEDDL
Dd LEATIKNITDMGFARDQVLRALRLTFNNAERAIEYLVSGNIP-AANDPEDEEEME---------------------

Sc FAQAAQGGNASSGALGTTGGATDAAQGGPPGSIGLTVEDLL------SLRQVVSGNPEALRPLLENISARYPQLREHIMA
Dd ---------------------------GGGGSGDNPFEALRNHPHFNLLREAISKNPSIIPGILQQLAQTNPALVRQIQE

Sc NPEVFVSMLLEAVGDNMQDVMEGADDMVEGEDIEVTGEAAAAGLGQGEGEGSFQVDYTPEDDQAISRLCEL-GFE
Dd NPNEFIR-------------------------------LFQGDGNPGGNPGQFTLQVTQEESEAIQRLQALTGMD

Sc RDLVIQVYFACDKNEEAAANILFSDHAD*
Dd KSTVIEAYFACDKNEELTASYLF-ETADDE*
```
